# An Unexpected Formation of Spiro Isoxazoline-Dihydrofurane Compounds from Substituted Ketofurfuryl Alcohols

**DOI:** 10.3390/molecules29225474

**Published:** 2024-11-20

**Authors:** Claire Cuyamendous, Mathieu Yves Laurent, Christine Saluzzo

**Affiliations:** MSO, Institut des Molécules et Matériaux du Mans (IMMM), UMR CNRS 6283, Le Mans Université, Avenue O. Messiaen, 72085 Le Mans, CEDEX 9, France; claire.cuyamendous@u-paris.fr (C.C.); mathieu.laurent@univ-lemans.fr (M.Y.L.)

**Keywords:** spiro compound, dihydroxazole, isoxazoline, furfural, oxime, cascade reaction, rearrangement, bis-heterocycle

## Abstract

Oximation of substituted ketofurfuryl alcohols in the presence of hydroxylamine hydrochloride and pyridine in ethanol as solvent led to a new class of spiro derivatives presenting a 7-methylene-1,6-dioxa-2-azaspiro [4.4] nona-2,8-diene skeleton along with, in some cases, the predictable oxime. The structures of such spiro compounds were determined by 2D NMR spectroscopy. The suggested formation of this skeleton involves an in situ oximation/dehydration/S_N_’ cascade reaction.

## 1. Introduction

**Scheme 1:**
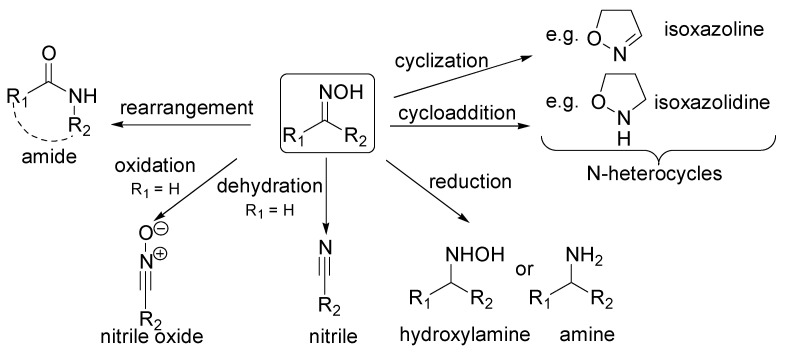
Overview of oximes transformations.

Oxime, a versatile functional group in organic chemistry, permits the introduction of a nitrogen atom into a chemical structure [1,2]. Derived from carbonyl groups, oximes can be transformed into amines [3,4], hydroxylamines [4,5], nitriles [6], nitrile oxides [7], amides [8], enamides [9], imines [9] and nitrones [10], but it can also be involved in the preparation of aza-heterocycles [1,2,11,12,13,14,15,16,17,18] (Scheme 1).

**Figure 1:**
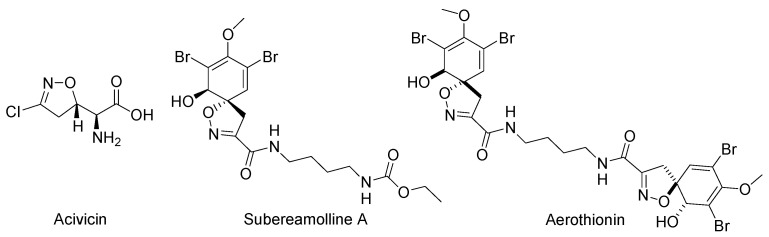
Some natural dihydroisoxazole derivatives.

Among nitrogen-containing heterocycles, 4,5–dihydroisoxazoles derivatives (five-membered ring bearing N-O bond) have attracted increasing attention due to their biological activities [19]. Some of them are natural products such as acivicin, produced by *Streptomyces sviceus* [20], and subereamolline A and aerothionin, isolated from marine sponges [21] (Figure 1). The latter two present an interesting spiroisoxazoline backbone for which Nishiyama developed a new electrochemical synthesis involving anodic oxidation [22].

**Scheme 2:**
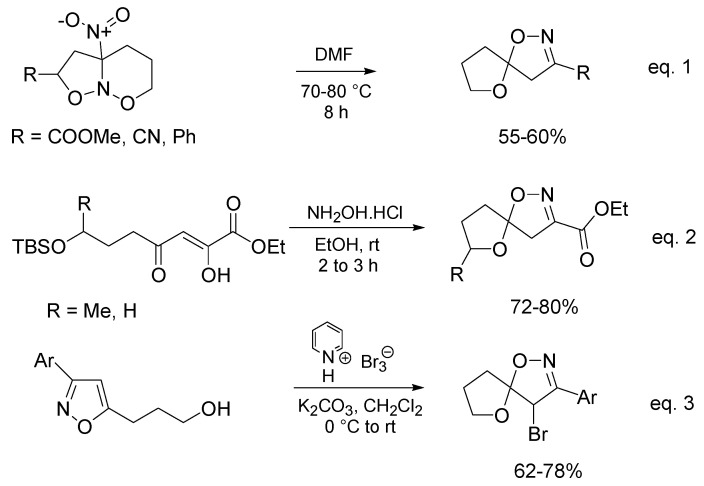
Syntheses of spiroisoxazolines presenting a THF unit.

The most common synthetic methods for isoxazolines typically involve 1,3-dipolar cycloaddition of nitrile oxide [23,24,25,26,27], nitronate [28,29] or oxime [30] with alkenes, including functionalized ones. On the other hand, the synthesis of spiroisoxazolines is more challenging. Moreover, to the best of our knowledge, isoxazolines are scarcely engaged in a spiro linked ring with a THF unit [31,32,33,34]. In 1978, Tarmakovskii et al. [31] synthesize for the first time a spiroisoxazoline with a THF unit using a thermal rearrangement of a nitrocyclic nitronate in dimethylformamide (DMF) (Scheme 2, Equation (1) (eq.1)). Twenty years later, Smietana et al. [32] have reported a spiro ketalization of an α-hydroxyiminoester (Scheme 2, Equation (2) (eq. 2)). The last methodolody reported by Das et al. [33] is based on the bromination with pyridinium tribromide of aromatic isoxazoles that contain a pendant alcohol (Scheme 2, Equation (3) (eq. 3)).

**Figure 2:**
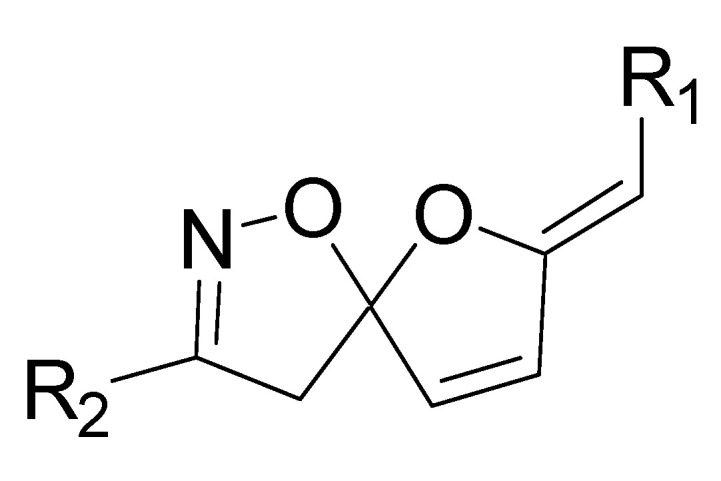
Structure of the new class of spiroisoxazoline.

We report herein the formation of a new class of spiro compounds presenting an isoxazoline unit along with a dihydrofurane moiety including a methylene group as shown in Figure 2.

## 2. Results and Discussion

**Scheme 3:**
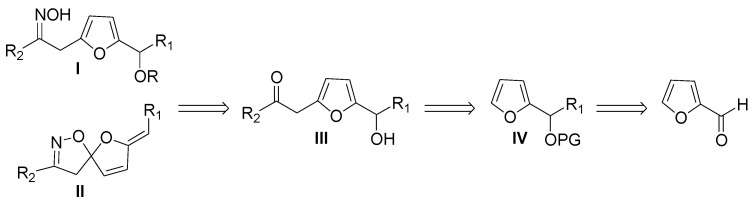
Retrosynthetic analysis of oxime/isoxazoline formation.

As indicated in Scheme 3, in our retrosynthetic analysis, ketoalcohol **III** would be a key intermediate to form the corresponding oxime **I** and/or the spiroderivative **II**. This ketoalcohol **III** could be generated by the functionalization in C-5 position of a 2-substituted furfurylalcohol **IV** easily obtained from furfuraldehyde, a biomass derivative.

### 2.1. Preparation of Ketoalcohol Key Intermediates

**Scheme 4:**
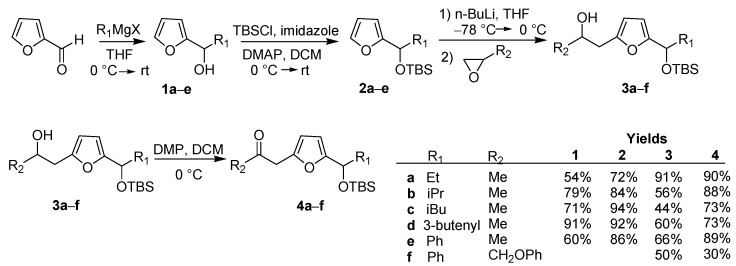
Synthesis of protected ketofurfuryl alcohols **4**.

A series of *tert*-butyldimethylsilyl ketofurfuryl alcohols **4a**–**e** presenting alkyl, alkenyl or aromatic groups were prepared from furfuraldehyde according to the four steps reaction sequence depicted in Scheme 4.

The first step involved the nucleophilic addition of a Grignard reagent to furfural giving the expected alcohols **1a**–**e** in 54 to 91% yields. After protection of the alcohol as *tert*-butyldimethylsilyloxy ether (OTBS), the C-5 functionalization of the THF ring was performed by lithiation reaction. The resulting anions reacted with methyl oxirane or with methylglycidyl ether providing the corresponding 2,5-disubtituted furans **3a**–**f** in good to excellent yields (44–91%). As already reported in the literature, the yield depends on the nature of both furan and epoxide derivatives involved in this reaction [35,36]. Subsequent oxidation of the free alcohol function of compounds **3a**–**f** was carried out with Dess–Martin periodinane reagent (DMP) to form compounds **4a**–**f** in 30 to 90% yields.

**Scheme 5:**
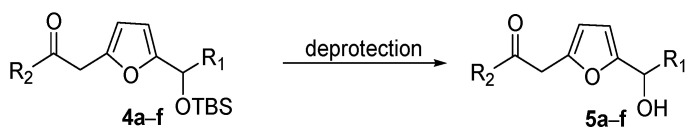
Deprotection of *tert*-butyldimethylsilyl.

Deprotection of *tert*-butyldimethylsilyl (OTBS) ethers **4a**–**f** was first performed with tetrabutylammonium fluoride (TBAF) (Scheme 5). Unfortunately, yields varied considerably from degradation to 87% (Table 1) and did not seem to be correlated with the nature of R_1_ and R_2_ substituents. The use of Et_3_N·3HF (Scheme 5) significantly improved the yields of products **5b**, **5d** and **5f** (entries 3, 6 and 9, Table 1). These differences in yield can be explained by the presence of moisture in the TBAF reagent, which decreases its reactivity compared to Et_3_N·3HF [37].

### 2.2. Oximation of Ketoalcohol 5

**Scheme 6:**
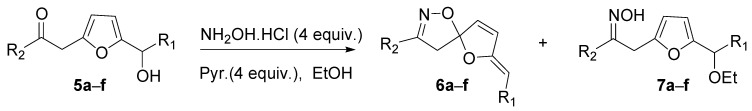
Oximation of ketones **5a**–**f**.

The oximation of ketones **5a**–**f** was then performed in ethanol according to the Han procedure [38]. However, in these conditions (i.e., hydroxylamine hydrochloride in the presence of pyridine), ketone **5a**–**f** led, in some cases, to spiroisoxazoline derivatives **6a**–**f** along with or without the expected oxime **7a**–**f** being protected as ethyl ether, the starting material having been completely consumed (Scheme 6 and Table 2). 

When the substituent R_1_ is an alkyl group, the obtained yield was very low and a mixture of expected oxime (12–34%) and spiroisoxazoline derivatives (1–16%) was observed (entries 1–4, Table 2). When the substituent R_1_ is a phenyl group, only the spiroisoxazoline derivatives **6e** and **6f** were isolated in good yield (entries 5 and 6, Table 2). This could be due to the mesomeric stabilization effect of the phenyl group on the conjugated exocyclic alkene.

**Figure 3:**
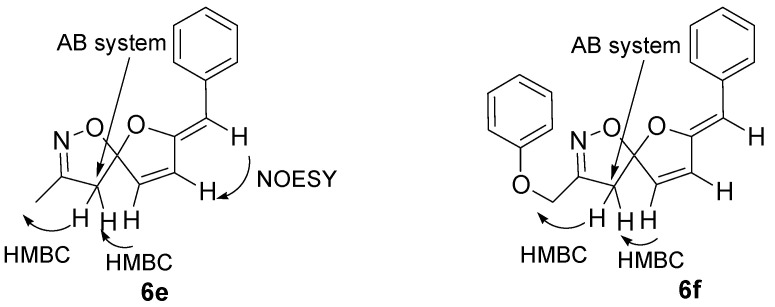
NOESY (S62 for **6e**) and HMBC (S61 for **6e**, S67 for **6f**) experiments for structure determination of compounds **6e** and **6f**.

Compared with the starting ketones **5e–f**, compounds **6e–f** were characterized from HRMS spectra by a lack of 3 units, compatible with the formation of the bicyclic structure (Scheme 6). Moreover, an AB system correlated to both R_2_ and vinylic proton (HMBC spectra S61 and S67) confirmed the cyclic isoxazoline backbone (Figure 3). Quaternary carbon of the spiro junction was observed at 119.9/120.2 ppm, suggesting a ketal carbon.

The NOESY experiment (S62) for compound **6e** was in accordance with *Z* stereochemistry of the exocyclic double bond as it showed an interaction between the two vinylic hydrogens (Figure 3). We assumed the same stereochemistry for compound **6f** according to the similarity between both spectra.

**Scheme 7:**
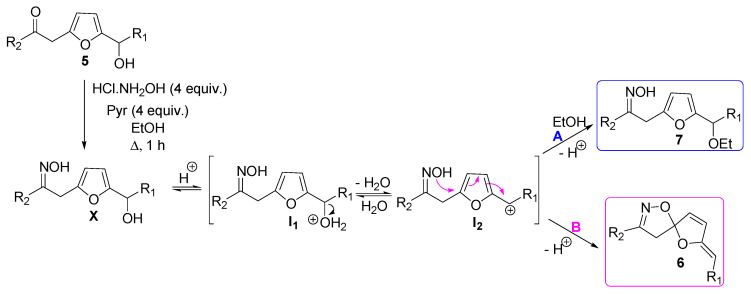
Suggested mechanism for the formation of spiranic isoxazole **6** and oxime **7**.

Based on these results, we proposed the following mechanism to explain the formation of these spiroheterocycles **6** and oximes **7** (Scheme 7). First of all, the free hydroxylamine formed in situ the expected oxime **X**. The hydroxyl group of the furfuryl alcohol moiety was then protonated leading to intermediate **I_1_**. Dehydration led to the carbocation **I_2_** which can evolved in two routes. Route A: a nucleophilic substitution with ethanol leading to oxime **7**; route B: an S_N_1′ involving the oxygen atom of the oxime resulting in a dearomatization of the furan ring by migration of the double bond, and then formation of the bicyclic compound **6** with no control of the stereochemistry of the chiral spiro center.

## 3. Materials and Methods

### 3.1. General

Reagents were purchased from commercial suppliers and used without additional purification. Furfuryl alcohols **1a** [39,40], **1b** [39,41], **1c** [39,42], **1d** [43,44,45] and **1e** [39,46] were synthesized according to the literature procedures, with Grignard reagents prepared. Dichloromethane, diethyl ether and THF were dried over activated alumina column in DRY STATION Glass Technology (Geneva, Switzerland) GTS 100 glassware. All reactions were conducted in oven-dried glassware, under inert atmosphere and were monitored by TLC. Column chromatography was performed using 60 μm silica gel. Thin-layer chromatography was performed with G/UV254 plates and revealed under UV light, then it was performed with phosphomolybdic acid or vanillin stains. All melting points were recorded on a ThermoFisher IA9300 apparatus and were uncorrected. The NMR spectra were acquired on a Bruker AVANCE 400 spectrometer operating at 400 and 100 MHz for ^1^H and ^13^C nuclei, respectively, and were recorded in CDCl_3_. Chemical shifts and coupling constants were presented in parts per million relative to Me_4_Si and Hertz, respectively. Abbreviations are as follows: s, singlet; d, doublet; t, triplet; q, quartet; m, multiplet; br s, broad signal. Proton and carbon assignments were established using COSY, HSQC, HMBC and DEPT experiments. Infrared spectra were recorded on an FT spectrophotometer from neat compounds using an ATR (Attenuated Total Reflexion) module. The wavenumbers of maximum absorption peaks of IR spectroscopy are presented in cm^−1^. High-resolution mass spectra were performed on a micrOTOF QIII Bruker mass spectrometer; the method used for HRMS was mentioned for each spectrum.

### 3.2. Synthesis and Characterization

#### 3.2.1. General Protocol for Alcohol Protection

To a solution of alcohol **1** (1 equivalent) in DCM (2 mL/mmol of alcohol), TBSCl (1.55 equivalent), imidazole (1.5 equivalent) and DMAP (0.01 eq.) were added at 0 °C. The reaction mixture was stirred overnight. Saturated aqueous solution of NH_4_Cl (2 mL/mmol of alcohol) was added and extracted with diethyl ether (3 × 1.5 mL/mmol of alcohol). The organic phases were dried over MgSO_4_, filtrated and concentrated under reduced pressure. The crude product was purified by column chromatography to afford protected product 2.

***tert*-Butyl-(1-furan-2-yl-propoxy)-dimethyl-silane (2a)**.

The crude product obtained from the alcohol **1a** (10.43 g, 82.7 mmol), TBSCl (18.87 g, 125 mmol, 1.51 equivalent), imidazole (8.25 g, 121 mmol, 1.46 equivalent) and DMAP (99 mg, 0.81 mmol, 0.01 equivalent) was purified by column chromatography on silica gel (cyclohexane/EtOAc 90/10) to give a colorless oil (14.40 g, 59.9 mmol, 72% yield). *Rf* 0.70 (cyclohexane/EtOAc 8/2); IR (neat) ν 2959, 2933, 2858, 1465, 1257, 1231, 1156, 1108, 1082, 1063, 1007, 840 cm^−1^; ^1^H NMR (400 MHz, CDCl_3_) δ 7.31 (1H, dd, *J* = 1.8, 0.9 Hz), 6.28 (1H, dd, *J* = 3.2, 1.8 Hz), 6.14 (1H, d, *J* = 3.2 Hz), 4.59 (1H, d, *J* = 6.5 Hz), 1.74–1.85 (2H, m), 0.87 (3H, t, *J* = 7.4 Hz), 0.86 (9H, s), 0.04 (3H, s), −0.07 (3H, s); ^13^C NMR (100 MHz, CDCl_3_) δ 157.6 (C_q_), 141.3 (CH), 110.1 (CH), 105.8 (CH), 70.1 (CH), 30.2 (CH_2_), 26.0 (3CH_3_), 18.5 (C_q_), 10.0 (CH_3_), −4.7 (CH_3_), −4.9 (CH_3_); HRMS (ESI^+^) *m*/*z* calcd for C_13_H_24_NaO_2_Si [M+Na]^+^: 263.1438, found 263.1435.

***tert*-Butyl-(1-furan-2-yl-2-methyl-propoxy)-dimethyl-silane (2b)**.

The crude product obtained from the alcohol **1b** (7.59 g, 54.1 mmol), TBSCl (12.73 g, 84.5 mmol, 1.56 equivalent), imidazole (5.57 g, 81.8 mmol, 1.5 equivalent) and DMAP (67 mg, 0.55 mmol, 0.01 equivalent) was purified by column chromatography on silica gel (cyclohexane/EtOAc 95/5) to give a colorless oil (11.64 g, 45.7 mmol, 84% yield). *Rf* 0.44 (cyclohexane/EtOAc 9/1); IR (neat) ν 2959, 1473, 1067, 1011, 840, 780, 732 cm^−1^; ^1^H NMR (400 MHz, CDCl_3_) δ 7.33 (1H, dd, *J* = 1.8, 0.8 Hz), 6.29 (1H, dd, *J* = 3.2, 1.8 Hz), 6.14 (1H, d, *J* = 3.2 Hz), 4.33 (1H, d, *J* = 6.9 Hz), 2.02 (1H, octuplet, *J* = 6.7 Hz), 0.95 (3H, d, *J* = 6.7 Hz), 0.88 (9H, s), 0.80 (3H, d, *J* = 6.8 Hz), 0.03 (3H, s), −0.14 (3H, s); ^13^C NMR (100 MHz, CDCl_3_) δ 156.9 (C_q_), 140.9 (CH), 109.8 (CH), 106.4 (CH), 74.2 (CH), 34.4 (CH), 25.8 (3CH_3_), 18.7 (CH_3_), 18.4 (CH_3_), 18.2 (C_q_), −5.0 (CH_3_), −5.2 (CH_3_); HRMS (ESI^+^) *m*/*z* calcd for C_14_H_26_NaO_2_Si [M+Na]^+^: 277.1600, found 277.1594.

***tert*-Butyl-(1-furan-2-yl-3-methyl-butoxy)-dimethyl-silane (2c)**.

The crude product obtained from the alcohol **1c** (8.89 g, 57.7 mmol), TBSCl (13.56 g, 90.0 mmol, 1.56 equivalent), imidazole (5.90 g, 86.7 mmol, 1.5 equivalent) and DMAP (71 mg, 0.58 mmol, 0.01 equivalent) was purified by column chromatography on silica gel (cyclohexane/EtOAc 95/5) to give a colorless oil (14.53 g, 54.1 mmol, 94% yield). *Rf* 0.71 (cyclohexane/EtOAc 9/1); IR (neat) ν 2959, 2933, 2858, 1473, 1257, 1086, 1007, 840, 780 cm^−1^; ^1^H NMR (400 MHz, CDCl_3_) δ 7.32 (1H, dd, *J* = 1.9, 0.8 Hz), 6.28 (1H, dd, *J* = 3.2, 1.9 Hz), 6.13 (1H, d, *J* = 3.2 Hz), 4.71 (1H, dd, *J* = 8.2, 5.8 Hz), 1.74–1.82 (1H, m), 1.69 (1H, sept., *J* = 6.7 Hz), 1.52–1.60 (1H, m), 0.87 (3H, d, *J* = 6.0 Hz), 0.86 (3H, d, *J* = 6.0 Hz), 0.85 (9H, s), 0.03 (3H, s), −0.12 (3H, s); ^13^C NMR (100 MHz, CDCl_3_) δ 157.8 (C_q_), 141.4 (CH), 110.2 (CH), 105.8 (CH), 66.9 (CH), 46.2 (CH_2_), 26.0 (3CH_3_), 24.5 (CH), 23.3 (CH_3_), 22.3 (CH_3_), 18.4 (C_q_), −4.6 (CH_3_), −4.9 (CH_3_); HRMS (ESI^+^) *m*/*z* calcd for C_15_H_28_NaO_2_Si [M+Na]^+^: 291.1751, found 291.1753.

***tert*-Butyl-(1-furan-2-yl-pent-4-enyloxy)-dimethyl-silane (2d)** [44,45].

The crude product obtained from the alcohol **1d** (14.11 g, 92.7 mmol), TBSCl (21.80 g, 144.6 mmol, 1.56 equivalent), imidazole (9.53 g, 140.0 mmol, 1.51 equivalent) and DMAP (114 mg, 93.3 mmol, 0.01 equivalent) was purified by column chromatography on silica gel (cyclohexane/EtOAc 90/10) to give a colorless oil (21.33 g, 80.1 mmol, 86% yield). *Rf* 0.69 (cyclohexane/EtOAc 9/1); IR (neat) ν 2933, 2858, 1644, 1477, 1351, 1257, 1098, 1011, 918, 840, 780 cm^−1^; ^1^H NMR (400 MHz, CDCl_3_) δ 7.32 (1H, dd, *J* = 1.8, 0.8 Hz), 6.28 (1H, dd, *J* = 3.2, 1.8 Hz), 6.15 (1H, ddd, *J* = 3.2, 0.8, 0.8 Hz), 5.79 (1H, ddt, *J* = 17.1, 10.2, 6.6 Hz), 5.02 (1H, ddt, *J* = 17.1, 1.7, 1.7 Hz), 4.98 (1H, ddt, *J* = 10.2, 1.7, 1.3 Hz), 4.68 (1H, dd, *J* = 7.2, 5.6 Hz), 2.00–2.14 (2H, m), 1.78–1.97 (2H, m), 0.87 (9H, s), 0.04 (3H, s), −0.09 (3H, s); ^13^C NMR (100 MHz, CDCl_3_) δ 157.4 (C_q_), 141.4 (CH), 138.5 (CH), 114.9 (CH_2_), 110.2 (CH), 105.9 (CH), 68.1 (CH), 36.4 (CH_2_), 29.8 (CH_2_), 26.0 (3CH_3_), 18.4 (C_q_), −4.7 (CH_3_), −4.9 (CH_3_); HRMS (ESI^+^) *m*/*z* calcd for C_15_H_26_NaO_2_Si [M+Na]^+^: 289.1594, found 289.1590.

***tert*-Butyl-(furan-2-yl-phenyl-methoxy)-dimethyl-silane (2e)** [47].

The crude product obtained from the alcohol **1e** (22.91 g, 132 mmol), TBSCl (30.9 g, 205 mmol, 1.55 equivalent), imidazole (13.51 g, 198 mmol, 1.5 equivalent) and DMAP (162 mg, 0.11 mmol, 0.01 equivalent) was purified by column chromatography on silica gel (cyclohexane/EtOAc 95/5) to give a colorless oil (35.03 g, 121 mmol, 92% yield). *Rf* 0.32 (cyclohexane/EtOAc 6/4); IR (neat) ν 2933, 1477, 1067, 1011 cm^−1^; ^1^H NMR (400 MHz, CDCl_3_) δ 7.40–7.44 (2H, m), 7.30–7.37 (3H, m), 7.27–7.30 (1H, m), 6,29 (1H, dd, *J* = 3.0, 1.7 Hz), 6.07 (1H, dd, *J* = 3.1, 0.7 Hz), 5.80 (1H, s), 0.93 (9H, s), 0.07 (3H, s), 0.04 (3H, s); ^13^C NMR (100 MHz, CDCl_3_) δ 157.0 (C_q_), 142.1 (C_q_), 142.0 (CH), 128.1 (2CH), 127.4 (CH), 126.3 (2CH), 110.0 (CH), 106.7 (CH), 70.6 (CH), 25.8 (3CH_3_), 18.3 (C_q_), −5.0 (2CH_3_); HRMS (ESI^+^) *m*/*z* calcd for C_17_H_24_NaO_2_Si [M+Na]^+^: 311.1443, found 311.1438; *m*/*z* calcd for C_17_H_24_KO_2_Si [M+K]^+^: 327.1183, found 327.1177.

#### 3.2.2. General Protocol for Epoxide Opening

At −78 °C, nBuLi solution (2 M in hexane,1 equivalent) was added dropwise to a solution of silylated alcohol **2** (1 equivalent) in THF (1.4 mL/mmol of silylated alcohol). The reaction mixture was slightly warmed to room temperature during 2h 30min, and epoxide (2 eq.) was added dropwise during 10 to 15 min. The reaction was stirred at room temperature overnight. Saturated aqueous solution of NH_4_Cl (1.4 mL/mmol of silylated alcohol) was added and extracted with diethyl ether (3 × 1 mL/mmol of silylated alcohol). The organic phases were dried over MgSO_4_, filtrated and concentrated under reduced pressure. The crude product 3 was purified by column chromatography.

**1-[5-[1-(*tert*-Butyl-dimethyl-silanyloxy)-propyl]-furan-2-yl]-propan-2-ol (3a)**.

The crude product obtained from the silylated alcohol **2a** (8.8 g, 36.6 mmol), *n*-BuLi (20.4 mL, 1.8 M 36.7 mmol, 1 equivalent) and methyl oxirane (5.1 mL, 73.3 mmol, 2 equivalent) was purified by column chromatography on silica gel (cyclohexane/EtOAc 90/10) to give a colorless oil (9.99 g, 33.5 mmol, 91% yield) as an inseparable 1:1 mixture of diastereoisomers. *Rf* 0.39 (cyclohexane/EtOAc 8/2); IR (neat) ν 3350, 2962, 2933, 2858, 1465, 1257, 1063, 1011, 840, 776 cm^−1^; ^1^H NMR (400 MHz, CDCl_3_) δ 6.06 (1H, d, *J* = 3.1 Hz), 6.01 (1H, d, *J* = 3.1 Hz), 4.54 (1H, t, *J* = 6.5 Hz), 4.00–4.08 (1H, m), 2.78 (1H, ddd, *J* = 14.9, 4.6, 1.8 Hz), 2.70 (1H, dd, *J* = 14.9, 7.6 Hz), 1.72–1.82 (3H, m), 1.23 (3H, d, *J* = 6.2 Hz), 0.89 (3H, d, *J* = 7.4 Hz), 0.87 (9H, s), 0.05 (3H, s), −0.051 and −0.055 (3H, 2s); ^13^C NMR (100 MHz, CDCl_3_) δ 156.53, 156.52, 151.42, 151.39 (2C_q_), 107.47, 107.46 (CH), 106.36, 106.35 (CH), 69.87, 69.86 (CH), 66.8, 66.7 (CH), 38.0 (CH_2_), 29.85, 29.84 (CH_2_), 25.8 (3CH_3_), 22.6 (CH_3_), 18.2 (C_q_), 10.0 (CH_3_), −4.8 (CH_3_), −5.0 (CH_3_); HRMS (ESI^+^) *m*/*z* calcd for C_16_H_30_NaO_3_Si [M+Na]^+^: 321.1859, found 321.1852.

**1-[5-[1-(*tert*-Butyl-dimethyl-silanyloxy)-2-methyl-propyl]-furan-2-yl]-propan-2-ol (3b)**.

The crude product obtained from the silylated alcohol **2b** (3,04 g, 11.9 mmol), *n*-BuLi (9.0 mL, 17.9 mmol, 1.5 equivalent) and methyl oxirane (1.67 mL, 23.9 mmol, 2 equivalent) was purified by column chromatography on silica gel (cyclohexane/EtOAc 98/2) to give a yellow oil (2.08 g, 6.66 mmol, 56% yield) as an inseparable 1:1 mixture of diastereoisomers. *Rf* 0.40 (cyclohexane/EtOAc 8/2); IR (neat) ν 3383, 2933, 2858, 1562, 1473, 1387, 1257, 1063, 940, 840, 776 cm^−1^; ^1^H NMR (400 MHz, CDCl_3_) δ 6.05 (1H, d, *J* = 3.0 Hz), 6.01 (1H, m), 4.26 and 4.25 (1H, 2d, *J* = 6.9 Hz), 4.00–4.11 (1H, m), 2.78 (1H, dt, *J* = 14.9, 4.7 Hz), 2.70 (1H, ddd, *J* = 14.8, 7.5, 5.0 Hz), 1.99 (1H, octuplet, *J =* 6.7 Hz), 1.77 and 1.75 (1H, 2d, *J* = 4.0 Hz), 1.22 (3H, d, *J* = 6.3 Hz), 0.95 and 0.94 (3H, 2d, *J* = 6.7 Hz), 0.86 (9H, s), 0.79 and 0.78 (3H, 2d, *J* = 6.7 Hz), 0.02 (3H, s), −0.136 and −0.138 (3H, 2s); ^13^C NMR (100 MHz, CDCl_3_) δ 156.02, 156.00, 151.24, 151.19 (2C_q_), 107.41, 107.35 (CH), 107.18, 107.13 (CH), 74.19, 74.15 (CH), 66.9, 66.7 (CH), 37.96, 37.95 (CH_2_), 34.3 (CH), 25.8 (3CH_3_), 22.5 (CH_3_), 18.7, 18.5, 18.4, 18.2 (CH_3_+C_q_), −4.9 (CH_3_), −5.2 (CH_3_); HRMS (ESI^+^) *m*/*z* calcd for C_17_H_33_O_3_Si [M+H]^+^: 313.2193, found 313.2198; calcd for C_17_H_32_NaO_3_Si [M+Na]^+^: 335.2015, found 335.2013; *m*/*z* calcd for C_17_H_32_KO_3_Si [M+K]^+^: 351.1748, found 351.1752.

**1-[5-[1-(*tert*-Butyl-dimethyl-silanyloxy)-3-methyl-butyl]-furan-2-yl]-propan-2-ol (3c)**.

The crude product obtained from the silylated alcohol **2c** (8.5 g, 31.7 mmol), *n*-BuLi (17.6 mL, 1.8 M, 31.7 mmol, 1 equivalent) and methyl oxirane (4.4 mL, 63.3 mmol, 2 equivalent) was purified by column chromatography on silica gel (cyclohexane/EtOAc 95/5) to give a colorless oil (4.56 g, 14.0 mmol, 44% yield) as an inseparable 1:1 mixture of diastereoisomers. *Rf* 0.31 (cyclohexane/EtOAc 8/2); IR (neat) ν 3330, 2959, 2933, 2858, 1562, 1469, 1365, 1257, 1082, 1011, 840, 780 cm^−1^; ^1^H NMR (400 MHz, CDCl_3_) δ 6.06 (1H, d, *J* = 3.0 Hz), 6.01 (1H, d, *J* = 3.0 Hz), 4.67 (1H, dd, *J* = 8.0, 5.7 Hz), 4.00–4.08 (1H, m), 2.78 (1H, ddd, *J* = 15.0, 4.3, 3.5 Hz), 2.70 (1H, ddd, *J* = 15.0, 7.6, 2.5 Hz), 1.71–1.84 (2H, m), 1.62–1.67 (1H, m), 1.45–1.54 (1H, m), 1.23 (3H, d, *J* = 6.2 Hz), 0.90 (3H, d, *J* = 6.6 Hz), 0.88 (3H, d, *J* = 6.6 Hz), 0.85 (9H, s), 0.04 (3H, s), −0.103 and −0.106 (3H, 2s); ^13^C NMR (100 MHz, CDCl_3_) δ 156.71, 156.68 (C_q_), 151.50, 151.45 (C_q_), 107.51, 107.46 (CH), 106.38, 106.37 (CH), 66.79, 66.65 (2CH), 45.8 (CH_2_), 38.0 (CH_2_), 25.8 (3CH_3_), 24.3 (CH), 23.1, 22.6, 22.0 (3CH_3_), 18.2 (C_q_), −4.8 (CH_3_), −5.1 (CH_3_); HRMS (ESI^+^) *m*/*z* calcd for C_18_H_34_NaO_3_Si [M+Na]^+^: 349.2169, found 349.2170.

**1-[5-[1-(*tert*-Butyl-dimethyl-silanyloxy)-pent-4-enyl]-furan-2-yl]-propan-2-ol (3d)**.

The crude product obtained from the silylated alcohol **2d** (8.53 g, 32.0 mmol), *n*-BuLi (17.8 mL, 1.8 M, 31.9 mmol, 1 equivalent) and methyl oxirane (4.5 mL, 63.9 mmol, 2 equivalent) was purified by column chromatography on silica gel (cyclohexane/EtOAc 90/10) to give a yellow oil (6.27 g, 19.3 mmol, 60% yield) as an inseparable 1:1 mixture of diastereoisomers. *Rf* 0.29 (cyclohexane/EtOAc 8/2); IR (neat) ν 3352, 2955, 2933, 2858, 1644, 1562, 1465, 1365, 1257, 1089, 1011, 840, 780 cm^−1^; ^1^H NMR (400 MHz, CDCl_3_) δ 6.07 (1H, d, *J* = 3.0 Hz), 6.02 (1H, d, *J* = 3.0 Hz), 5.80 (1H, ddt, *J* = 17.3, 10.3, 6.3 Hz), 5.01 (1H, dq, *J* = 17.3, 1.7 Hz), 4.95 (1H, dm, *J* = 10.4 Hz), 4.63 (1H, dd, *J* = 7.2, 5.6 Hz), 4.00–4.09 (1H, m), 2.78 (1H, ddd, *J* = 15.0, 4.8, 2.9 Hz), 2.71 (1H, ddd, *J* = 15.0, 7.6, 2.3 Hz), 1.97–2.18 (2H, m), 1.70–1.97 (3H, m), 1.23 (3H, d, *J* = 6.2 Hz), 0.87 (9H, s), 0.04 (3H, s), −0.078 and −0.083 (3H, 2s); ^13^C NMR (100 MHz, CDCl_3_) δ 156.29, 156.28 (C_q_), 151.57, 151.53 (C_q_), 138.2 (CH), 114.7 (CH_2_), 107.52, 107.48 (CH), 106.53, 106.51 (CH), 67.8 (CH), 66.8, 66.7 (CH), 38.0 (CH_2_), 36.0 (CH_2_), 29.7 (CH_2_), 25.8 (3CH_3_), 22.6 (CH_3_), 18.2 (C_q_), −4.8 (CH_3_), −5.0 (CH_3_); HRMS (ESI^+^) *m*/*z* calcd for C_18_H_32_NaO_3_Si [M+Na]^+^: 347.2013, found 347.2006.

**1-[5-[(*tert*-Butyl-dimethyl-silanyloxy)-phenyl-methyl]-furan-2-yl]-propan-2-ol (3e)**.

The crude product obtained from the silylated alcohol **2e** (5.0 g, 17.3 mmol), *n*-BuLi (8.66 mL, 17.33 mmol, 1 equivalent) and methyl oxirane (2.43 mL, 34.66 mmol, 2 equivalent) was purified by column chromatography on silica gel (cyclohexane/EtOAc 95/5) to give a colorless oil (3.94 g, 11.4 mmol, 66% yield) as an inseparable 1:1 mixture of diastereoisomers. *Rf* 0.58 (cyclohexane/EtOAc 8/2); IR (neat) ν 3406, 2858, 1722, 1689, 1454, 1067, 1015, 1086, 840 cm^−1^; ^1^H NMR (400 MHz, CDCl_3_) δ 7.35–7.42 (2H, m), 7.30–7.35 (2H, m), 7.20–7.29 (1H, m), 5.99 (1H, d, *J* = 3.1 Hz), 5.91 (1H, d, *J* = 3.1 Hz), 5.74 (1H, s), 3.95–4.08 (1H, m), 2.75 (1H, dd, *J* = 14.9, 4.6 Hz), 2.68 (1H, dd, *J* = 14.9, 7.5 Hz), 1.77 (1H, bs), 1.20 (3H, dd, *J* = 6.1, 0.6 Hz), 0.91 (9H, s), 0.05 and 0.034 (3H, 2s), 0.029 and 0.00 (3H, 2s); ^13^C NMR (100 MHz, CDCl_3_) δ 156.2, 156.1 (C_q_), 152.3 (C_q_), 141.9 (C_q_), 128.1 (2CH), 127.4 (CH), 126.3 (2CH), 107.7, 107.59 (CH), 107.56 (CH), 70.53, 70.48 (CH), 66.7 (CH), 37.9 (CH_2_), 25.8 (3CH_3_), 22.6 (CH_3_), 18.3 (C_q_), −5.0 (2CH_3_); HRMS (ESI^+^) *m*/*z* calcd for C_20_H_30_NaO_3_Si [M+Na]^+^: 369.1862, found 369.1856; *m*/*z* calcd for C_20_H_30_KO_3_Si [M+K]^+^: 385.1601, found 385.1596.

**1-[5-[(*tert*-Butyl-dimethyl-silanyloxy)-phenyl-methyl]-furan-2-yl]-3-phenoxy-propan-2-ol (3f)**.

The crude product obtained from the silylated alcohol **2f** (2.0 g, 6.93 mmol), *n*-BuLi (6.1 mL, 1.14 M, 6.94 mmol, 1 equivalent) and 2-phenoxymethyl-oxirane (1.9 mL, 13.88 mmol, 2 equivalent) was purified by column chromatography on silica gel (cyclohexane/EtOAc 90/10) to give a colorless oil (1.52 g, 3.47 mmol, 50% yield). *Rf* 0.35 (cyclohexane/EtOAc 8/2); IR (neat) ν 2955, 2929, 2858, 1603, 1499, 1249, 1086, 840, 754 cm^−1^; ^1^H NMR (400 MHz, CDCl_3_) δ 7.38–7.41 (2H, m), 7.23–7.35 (4H, m), 6.82–6.99 (4H, m), 6.03 (1H, d, *J* = 3.1 Hz), 5.94 (1H, d, *J* = 3.1 Hz), 5.73 (1H, s), 4.19–4.25 (1H, m), 3.93 (1H, ddd, *J* = 9.4, 3.6, 3.6 Hz), 3.86 (1H, ddd, *J* = 9.4, 6.5, 3.8 Hz), 2.93 (2H, d, *J* = 6.4 Hz), 2.38 (1H, bs), 0.90 (9H, s), 0.04 (3H, s), 0.02 (3H, s); ^13^C NMR (100 MHz, CDCl_3_) δ 158.5 (C_q_), 156.2 (C_q_), 151.2 (C_q_), 141.9 (C_q_), 129.5 (2CH), 128.1 (2CH), 127.4 (CH), 126.3 (2CH), 121.1 (CH), 114.6 (2CH), 107.9 (CH), 107.7 (CH), 70.9 (CH_2_), 70.5 (CH), 69.0 (CH), 32.4 (CH_2_), 25.8 (3CH_3_), 18.3 (C_q_), −5.0 (2CH_3_); HRMS (ESI^+^) *m*/*z* calcd for C_26_H_34_NaO_4_Si [M+Na]^+^: 461.2119, found 461.2110.

#### 3.2.3. General Protocol for Alcohol Oxidation to Ketone

A solution of alcohol **3** (1 eq.) in DCM (2 mL/mmol of alcohol) was degassed by bubbling a flush of argon. This solution was cooled to 0 °C and DMP (1.2 equivalent) was added. The reaction mixture was stirred at 0 °C during 1h and 2h 30min at room temperature. The reaction was quenched by adding a saturated solution of Na_2_S_2_O_3_ (2 mL/mmol of alcohol) and a saturated aqueous solution of NaHCO_3_ (1 mL/mmol of alcohol). The reaction mixture was then extracted with DCM (3 × 1 mL/mmol of alcohol). The organic phase was dried over MgSO_4_, filtrated and concentrated under reduced pressure. The crude product was purified by column chromatography to afford ketone **4**.

**1-[5-[1-(*tert*-Butyl-dimethyl-silanyloxy)-propyl]-furan-2-yl]-propan-2-one (4a)**.

The crude product obtained from the alcohol **3a** (0.371 g, 1.24 mmol) and DMP (0.764 g, 1.80 mmol, 1.45 equivalent) was purified by column chromatography on silica gel (cyclohexane/EtOAc 90/10) to give a colorless oil (0.332 g, 1.12 mmol, 90% yield). *Rf* 0.48 (cyclohexane/EtOAc 8/2); IR (neat) ν 2959, 2933, 2858, 1723, 1465, 1361, 1127, 1104, 1063, 1019, 911, 840, 780 cm^−1^; ^1^H NMR (400 MHz, CDCl_3_) δ 6.11 (2H, s), 4.55 (1H, dd, *J* = 6.5, 6.5 Hz), 3.66 (2H, s), 2.14 (3H, s), 1.73–1.80 (2H, m), 0.86 (3H, t, *J* = 7.3 Hz), 0.85 (9H, s), 0.03 (3H, s), −0.06 (3H, s); ^13^C NMR (100 MHz, CDCl_3_) δ 204.4 (C_q_), 157.2 (C_q_), 146.9 (C_q_), 108.7 (CH), 106.7 (CH), 69.8 (CH), 43.4 (CH_2_), 29.9 (CH_2_), 29.0 (CH_3_), 25.8 (3CH_3_), 18.2 (C_q_), 9.8 (CH_3_), −4.8 (CH_3_), −5.0 (CH_3_); HRMS (ESI^+^) *m*/*z* calcd for C_16_H_28_NaO_3_Si [M+Na]^+^: 319.1700, found 319.1700.

**1-[5-[1-(tert-Butyl-dimethyl-silanyloxy)-2-methyl-propyl]-furan-2-yl]-propan-2-one (4b)**.

The crude product obtained from the alcohol **3b** (1.16 g, 3.71 mmol) and DMP (2.36 g, 5.58 mmol, 1.5 equivalent) was purified by column chromatography on silica gel (cyclohexane/EtOAc 85/15) to give a yellow oil (1.02 g, 3.28 mmol, 88% yield). *Rf* 0.55 (cyclohexane/EtOAc 85/15); IR (neat) ν 3473, 2959, 2933, 2858, 1722, 1596, 1514, 1473, 1391, 1365, 1257, 1071, 1026, 1011 cm^−1^; ^1^H NMR (400 MHz, CDCl_3_) δ 6.11 (1H, d, *J* = 3.2 Hz), 6.09 (1H, d, *J* = 3.2 Hz), 4.27 (1H, d, *J* = 6.9 Hz), 3.66 (2H, s), 2.14 (3H, s), 2.00 (1H, octuplet, *J =* 6.7 Hz), 0.94 (3H, d, *J* = 6.7 Hz), 0.87 (9H, s), 0.79 (3H, d, *J* = 6.7 Hz), 0.03 (3H, s), −0.12 (3H, s); ^13^C NMR (100 MHz, CDCl_3_) δ 204.4 (C_q_), 156.7 (C_q_), 146.8 (C_q_), 108.6 (CH), 107.5 (CH), 74.1 (CH), 43.5 (CH_2_), 34.3 (CH), 28.9 (CH_3_), 25.8 (3CH_3_), 18.7 (CH_3_), 18.3 (CH_3_), 18.2 (C_q_), −4.9 (CH_3_), −5.2 (CH_3_); HRMS (ESI^+^) *m*/*z* calcd for C_17_H_30_NaO_3_Si [M+Na]^+^: 333.1856, found 333.1862.

**1-[5-[1-(*tert*-Butyl-dimethyl-silanyloxy)-3-methyl-butyl]-furan-2-yl]-propan-2-one (4c)**.

The crude product obtained from the alcohol **3c** (1.89 g, 5.79 mmol) and DMP (3.69 g, 8.70 mmol, 1.5 equivalent) was purified by column chromatography on silica gel (cyclohexane/EtOAc 90/10) to give a yellow oil (1.38 g, 4.25 mmol, 73% yield). *Rf* 0.63 (cyclohexane/EtOAc 8/2); IR (neat) ν 2959, 2933, 2858, 1722, 1689, 1469, 1365, 1253, 1086, 1007, 840, 780 cm^−1^; ^1^H NMR (400 MHz, CDCl_3_) δ 6.11 (2H, s), 4.68 (1H, dd, *J* = 8.1, 5.9 Hz), 3.67 (2H, s), 2.15 (3H, s), 1.48–1.79 (3H, m), 0.90 (3H, d, *J* = 6.1 Hz), 0.89 (3H, d, *J* = 6.1 Hz), 0.85 (9H, s), 0.05 (3H, s), −0.09 (3H, s); ^13^C NMR (100 MHz, CDCl_3_) δ 204.3 (C_q_), 157.4 (C_q_), 147.0 (C_q_), 108.7 (CH), 106.7 (CH), 66.7 (CH), 46.0 (CH_2_), 43.4 (CH_2_), 29.0 (CH_3_), 25.8 (3CH_3_), 24.3 (CH), 23.1 (CH_3_), 22.1 (CH_3_), 18.2 (C_q_), −4.7 (CH_3_), −5.1 (CH_3_); HRMS (ESI^+^) *m*/*z* calcd for C_18_H_32_NaO_3_Si [M+Na]^+^: 347.2013, found 347.2014.

**1-[5-[1-(*tert*-Butyl-dimethyl-silanyloxy)-pent-4-enyl]-furan-2-yl]-propan-2-one (4d)**.

The crude product obtained from the alcohol **3d** (1.58 g, 4.87 mmol) and DMP (3.09 g, 7.29 mmol, 1.5 equivalent) was purified by column chromatography on silica gel (cyclohexane/EtOAc 95/5) to give a yellow oil (1.15 g, 3.57 mmol, 73% yield). *Rf* 0.53 (cyclohexane/EtOAc 8/2); IR (neat) ν 3078, 2955, 2933, 2858, 1722, 1477, 1361, 1257, 1093, 1011, 914, 840, 780 cm^−1^; ^1^H NMR (400 MHz, CDCl_3_) δ 6.11 (2H, s), 5.80 (1H, ddt, *J* = 17.1, 10.3, 6.7 Hz), 5.01 (1H, dm, *J* = 17.1 Hz), 4.95 (1H, dm, *J* = 10.3 Hz), 4.64 (1H, dd, *J* = 7.3, 5.6 Hz), 3.67 (2H, s), 2.15 (3H, s), 1.97–2.13 (2H, m), 1.71–1.95 (2H, m), 0.87 (9H, s), 0.05 (3H, s), −0.07 (3H, s); ^13^C NMR (100 MHz, CDCl_3_) δ 204.3 (C_q_), 157.0 (C_q_), 147.1 (C_q_), 138.2 (CH), 114.8 (CH_2_), 108.7 (CH), 106.9 (CH), 67.8 (CH), 43.4 (CH_2_), 36.1 (CH_2_), 29.6 (CH_2_), 29.0 (CH_3_), 25.8 (3CH_3_), 18.2 (C_q_), −4.8 (CH_3_), −5.0 (CH_3_); HRMS (ESI^+^) *m*/*z* calcd for C_18_H_30_NaO_3_Si [M+Na]^+^: 345.1856, found 345.1861.

**1-[5-[(*tert*-Butyl-dimethyl-silanyloxy)-phenyl-methyl]-furan-2-yl]-propan-2-one (4e)**.

The crude product obtained from the alcohol **3e** (3.9 g, 11.3 mmol) and DMP (5.73 g, 13.5 mmol, 1.2 equivalent) was purified by column chromatography on silica gel (cyclohexane/EtOAc 90/10) to give a colorless oil (3.46 g, 10.0 mmol, 89% yield). *Rf* 0.50 (cyclohexane/EtOAc 7/3); IR (neat) ν 2955, 2933, 2858, 1715, 1603, 1257, 1182, 840, 780 cm^−1^; ^1^H NMR (400 MHz, CDCl_3_) δ 7.25–7.41 (5H, m), 6.09 (1H, d, *J* = 3.2 Hz), 5.99 (1H, d, *J* = 3.2 Hz), 5.74 (1H, s), 3.63 (2H, s), 2.10 (3H, s), 0.91 (9H, s), 0.05 (3H, s), 0.04 (3H, s); ^13^C NMR (100 MHz, CDCl_3_) δ 204.3 (C_q_), 156.8 (C_q_), 147.8 (C_q_), 141.9 (C_q_), 128.1 (2CH), 127.4 (CH), 126.3 (2CH), 108.8 (CH), 107.9 (CH), 70.5 (CH), 43.4 (CH_2_), 29.0 (3CH_3_), 25.8 (CH_3_), 18.3 (C_q_), −5.0 (2CH_3_); HRMS (ESI^+^) *m*/*z* calcd for C_20_H_28_NaO_3_Si [M+Na]^+^: 367.1705, found 367.1697.

**1-[5-[(*tert*-Butyl-dimethyl-silanyloxy)-phenyl-methyl]-furan-2-yl]-3-phenoxy-propan-2-one (4f)**.

The crude product obtained from the alcohol **3f** (0.345 g, 0.79 mmol) and DMP (0.501 g, 1.18 mmol, 1.45 equivalent) was purified by column chromatography on silica gel (cyclohexane/EtOAc 95/5) to give a colorless oil (103 mg, 0.24 mmol, 30% yield). *Rf* 0.69 (cyclohexane/EtOAc 8/2); IR (neat) ν 2955, 2933, 2858, 1737, 1603, 1499, 1250, 1063, 1022, 840, 758 cm^−1^; ^1^H NMR (400 MHz, CDCl_3_) δ 7.39 (2H, d, *J* = 8.0 Hz), 7.23–7.35 (5H, m), 6.99 (1H, t, *J* = 8.0 Hz), 6.81 (2H, d, *J* = 8.7 Hz), 6.14 (1H, d, *J* = 3.2 Hz), 6.01 (1H, d, *J* = 3.2 Hz), 5.73 (1H, s), 4.56 (2H, s), 3.85 (1H, s), 0.90 (9H, s), 0.04 (3H, s), 0.03 (3H, s); ^13^C NMR (100 MHz, CDCl_3_) δ 202.0 (C_q_), 157.6 (C_q_), 157.0 (C_q_), 146.3 (C_q_), 141.9 (C_q_), 129.6, 128.1, 127.4, 126.3, 121.7, 114.6 (10CH), 109.2 (CH), 107.9 (CH), 72.0 (CH_2_), 70.5 (CH), 39.3 (CH_2_), 25.8 (3CH_3_), 18.3 (C_q_), −5.0 (2CH_3_); HRMS (ESI^+^) *m*/*z* calcd for C_26_H_32_NaO_4_Si [M+Na]^+^: 459.1962, found 459.1957.

#### 3.2.4. General Protocol for Silylated Alcohol Deprotection

Procedure with TBAF reagent: A solution of TBAF (1.0 M in THF, 1.5 equivalent) at room temperature was added to a solution of silylated alcohol **4** (1 equivalent) in anhydrous THF (2 mL/mmol of silylated alcohol). The reaction mixture was followed by TLC and stopped when starting material completely disappeared (1 or 2 h). A saturated solution of NH_4_Cl (2 mL/mmol of silylated alcohol) was added. The reaction mixture was then extracted with EtOAc (3 × 1 mL/mmol of silylated alcohol). The organic phases were dried over MgSO_4_, filtrated and concentrated under reduced pressure. The crude product was purified by column chromatography to afford product **5**.

Procedure with Et_3_N.3HF reagent: A solution of Et_3_N.3HF (1.5 equivalent) at room temperature was added to a solution of the silylated alcohol **4** (1 equivalent) in anhydrous acetonitrile (1.25 mL/mmol of silylated alcohol). The reaction mixture was followed by TLC and stopped when starting material completely disappeared (1 or 2 h). A saturated solution of NaHCO_3_ (2 mL/mmol of silylated alcohol) was added. The reaction mixture was then extracted with CH_2_Cl_2_ (3 × 1 mL/mmol of silylated alcohol). The organic phases were dried over MgSO_4_, filtrated and concentrated under reduced pressure. The crude product was purified by column chromatography to afford product **5**.

**1-[5-(1-Hydroxy-propyl)-furan-2-yl]-propan-2-one (5a)**.

The crude product obtained from the silylated alcohol **4a** (0.332 g, 1.12 mmol) and TBAF (1.65 mL, 1.65 mmol, 1.5 equivalent) was purified by column chromatography on silica gel (cyclohexane/EtOAc 95/5) to give a colorless oil (81 mg, 0.44 mmol, 40% yield). *Rf* 0.39 (cyclohexane/EtOAc 8/2); IR (neat) ν 3413, 2970, 2936, 2880, 1719, 1421, 1361, 1242, 1026, 1048, 966, 795 cm^−1^; ^1^H NMR (400 MHz, CDCl_3_) δ 6.19 (1H, d, *J* = 3.1 Hz), 6.14 (1H, d, *J* = 3.1 Hz), 4.56 (1H, dd, *J* = 6.8, 6.8 Hz), 3.70 (2H, s), 2.17 (3H, s), 1.70–1.90 (2H, m), 0.95 (3H, t, *J* = 7.3 Hz); ^13^C NMR (100 MHz, CDCl_3_) δ 204.2 (C_q_), 156.4 (C_q_), 147.6 (C_q_), 108.8 (CH), 107.0 (CH), 69.1 (CH), 43.3 (CH_2_), 29.2 (CH_3_), 28.8 (CH_2_), 9.9 (CH_3_); HRMS (ESI^+^) *m*/*z* calcd for C_10_H_14_NaO_3_ [M+Na]^+^: 205.0835, found 205.0834.

**1-[5-(1-Hydroxy-2-methyl-propyl)-furan-2-yl]-propan-2-one (5b)**.

The crude product obtained from the silylated alcohol **4b** (1.28 g, 4.12 mmol) and Et_3_N.3HF (1.0 mL, 6.18 mmol, 1.5 equivalent) was purified by column chromatography on silica gel (cyclohexane/EtOAc 95/5) to give a yellow oil (521 mg, 2.65 mmol, 64 % yield). *Rf* 0.38 (cyclohexane/EtOAc 6/4); IR (neat) ν 3435, 2962, 2929, 2877, 1715, 1562, 1473, 1387, 1361, 1298, 1242, 1212, 1067, 1022 cm^−1^; ^1^H NMR (400 MHz, CDCl_3_) δ 6.19 (1H, d, *J* = 3.1 Hz), 6.14 (1H, d, *J* = 3.1 Hz), 4.33 (1H, d, *J* = 7.3 Hz), 3.69 (2H, s), 2.16 (3H, s), 2.08 (1H, octuplet, *J =* 6,8 Hz), 1.88–2.02 (1H, bs), 1.01 (3H, d, *J* = 6.8 Hz), 0.86 (3H, d, *J* = 6.8 Hz); ^13^C NMR (100 MHz, CDCl_3_) δ 204.1 (C_q_), 155.9 (C_q_), 147.4 (C_q_), 108.8 (CH), 107.6 (CH), 73.5 (CH), 43.3 (CH_2_), 33.2 (CH), 29.1 (CH_3_), 18.7 (CH_3_), 18.2 (CH_3_); HRMS (ESI^+^) *m*/*z* calcd for C_11_H_16_NaO_3_ [M+Na]^+^: 219.0988, found 219.0992.

**1-[5-(1-Hydroxy-3-methyl-butyl)-furan-2-yl]-propan-2-one (5c)**.

The crude product obtained from the silylated alcohol **4c** (0.695 g, 2.14 mmol) and TBAF (3.22 mL, 3.21 mmol, 1.5 equivalent) was purified by column chromatography on silica gel (cyclohexane/EtOAc 95/5) to give a yellow oil (273 mg, 1.30 mmol, 61% yield). *Rf* 0.43 (PE/EtOAc 6/4); IR (neat) ν 3435, 2959, 2925, 2873, 2854, 1719, 1599, 1514, 1469, 1391, 1361, 1223, 1160, 1071, 1022 cm^−1^; ^1^H NMR (400 MHz, CDCl_3_) δ 6.16 (1H, d, *J* = 3.1 Hz), 6.11 (1H, d, *J* = 3.1 Hz), 4.69 (1H, dd, *J* = 5.7, 5.7 Hz), 3.68 (2H, s), 2.16 (3H, s), 1.60–1.80 (4H, m), 0.93 (3H, d, *J* = 6.2 Hz), 0.91 (3H, d, *J* = 6.2 Hz); ^13^C NMR (100 MHz, CDCl_3_) δ 204.2 (C_q_), 156.9 (C_q_), 147.5 (C_q_), 108.8 (CH), 106.8 (CH), 65.9 (CH), 44.4 (CH_2_), 43.2 (CH_2_), 29.1 (CH_3_), 24.6 (CH), 23.0 (CH), 22.1 (2CH_3_); HRMS (ESI^+^) *m*/*z* calcd for C_12_H_18_NaO_3_ [M+Na]^+^: 233.1148, found 233.1140.

**1-[5-(1-Hydroxy-pent-4-enyl)-furan-2-yl]-propan-2-one (5d)**.

The crude product obtained from the silylated alcohol **4d** (1.16 g, 3.60 mmol) and Et_3_N.3HF (0.89 mL, 5.39 mmol, 1.5 equivalent) was purified by column chromatography on silica gel (cyclohexane/EtOAc 90/10) to give a yellow oil (440 mg, 2.11 mmol, 59% yield). *Rf* 0.20 (cyclohexane/EtOAc 7/3); IR (neat) ν 3406, 3078, 2977, 2925, 2858, 1715, 1644, 1562, 1361, 1242, 1212, 1164, 1067, 1015 cm^−1^; ^1^H NMR (400 MHz, CDCl_3_) δ 6.20 (1H, d, *J* = 3.2 Hz), 6.14 (1H, d, *J* = 3.2 Hz), 5.83 (1H, ddt, *J* = 17.3, 10.3, 6.6 Hz), 5.05 (1H, dm, *J* = 17.2 Hz), 4.99 (1H, dm, *J* = 10.4 Hz), 4.67 (1H, t, *J* = 6.8 Hz), 3.70 (2H, s), 2.07–2.28 (2H, m), 2.18 (3H, s), 1.93 (3H, td, *J* = 7.1, 7.1 Hz); ^13^C NMR (100 MHz, CDCl_3_) δ 204.0 (C_q_), 156.3 (C_q_), 147.7 (C_q_), 137.8 (CH), 115.2 (CH_2_), 108.9 (CH), 107.1 (CH), 67.2 (CH), 43.3 (CH_2_), 34.5 (CH_2_), 29.8 (CH_2_), 29.2 (CH_3_); HRMS (ESI^+^) *m*/*z* calcd for C_12_H_16_NaO_3_ [M+Na]^+^: 231.0992, found 231.0990.

**1-[5-(Hydroxy-phenyl-methyl)-furan-2-yl]-propan-2-one (5e).**

The crude product obtained from the silylated alcohol **4e** (3.38 g, 9.81 mmol) and TBAF (14.7 mL, 14.7 mmol, 1.5 equivalent) was purified by column chromatography on silica gel (cyclohexane/EtOAc 95/5) to give a colorless oil (1.97 g, 8.56 mmol, 87% yield). *Rf* 0.17 (cyclohexane/EtOAc 7/3); IR (neat) ν 3406, 3063, 3029, 2959, 2925, 2854, 1715, 1599, 1357, 1160, 963, 724, 702 cm^−1^; ^1^H NMR (400 MHz, CDCl_3_) δ 7.27–7.43 (5H, m), 6.11 (1H, d, *J* = 3.2 Hz), 6.03 (1H, d, *J* = 3.2 Hz), 5.76 (1H, s), 3.66 (2H, s), 2.85 (1H, bs), 2.12 (3H, s); ^13^C NMR (100 MHz, CDCl_3_) δ 204.2 (C_q_), 155.7 (C_q_), 148.2 (C_q_), 140.7 (C_q_), 128.3 (2CH), 127.9 (CH), 126.5 (2CH), 108.9 (CH), 108.5 (CH), 69.9 (CH), 43.1 (CH_2_), 29.2 (CH_3_); HRMS (ESI^+^) *m*/*z* calcd for C_14_H_14_NaO_3_ [M+Na]^+^: 253.0835, found 253.0829.

**1-[5-(Hydroxy-phenyl-methyl)-furan-2-yl]-3-phenoxy-propan-2-one (5f).**

The crude product obtained from the silylated alcohol **4f** (1.03 g, 2.36 mmol) and Et_3_N.3HF (0.65 mL, 4.0 mmol, 1.7 equivalent) was purified by column chromatography on silica gel (cyclohexane/EtOAc 9/1) to give a colorless oil (658 mg, 2.04 mmol, 86% yield). *Rf* 0.47 (cyclohexane/EtOAc 7/3); IR (neat) ν 3426, 3062, 2919, 1736, 1598, 1494, 1241, 1080, 1058, 1016 cm^−1^; ^1^H NMR (400 MHz, CDCl_3_) δ 7.25–7.45 (7H, m), 7.00 (1H, t, *J* = 7.3 Hz), 6.84 (2H, dd, *J* = 8.7, 1.0 Hz), 6.17 (1H, d, *J* = 3.1 Hz), 6.05 (1H, d, *J* = 3.1 Hz), 5.78 (1H, s), 4.59 (2H, s), 3.91 (2H, s), 2.43 (1H, bs); ^13^C NMR (100 MHz, CDCl_3_) δ 202.3 (C_q_), 157.6 (C_q_), 155.8 (C_q_), 147.0 (C_q_), 140.6 (C_q_), 129.7, 128.5, 128.1, 126.6, 121.8, 114.5 (10CH), 109.4 (CH), 108.7 (CH), 72.2 (CH_2_), 70.1 (CH), 39.1 (CH_2_); HRMS (ESI^+^) *m*/*z* calcd for C_20_H_18_KO_4_ [M+K]^+^: 361.0842, found 361.1036.

#### 3.2.5. General Protocol for Oxime Formation

NH_2_OH.HCl (4 eq.) and pyridine (4 eq.) were added to a solution of ketone **5** (1 equivalent) in ethanol (5 mL/mmol of ketone). After heating 1h at reflux, the reaction mixture was diluted with water (5 mL/mmol of ketone) and then extracted with EtOAc (3 × 5 mL/mmol of ketone). The organic phases were dried over MgSO_4_, filtrated and concentrated under reduced pressure. The crude product was purified by column chromatography to afford products 6 and 7.

**1-[5-(1-Ethoxy-propyl)-furan-2-yl]-propan-2-one oxime (7a).**

The crude product obtained from the ketone **5a** (191 mg, 1.049 mmol), NH_2_OH.HCl (292 mg, 4.20 mmol, 4 equivalent) and pyridine (0.34 mL, 4.20 mmol, 4 equivalent) give a mixture which was purified by column chromatography on silica gel (cyclohexane/EtOAc 8/2) to give a colorless oil (81.1 mg, 0.36 mmol, 34% yield) as an inseparable 9:1 mixture of *E*:*Z* oximes **7a**. *Rf* 0.34 (cyclohexane/EtOAc 6/4); IR (neat) ν 3292, 2971, 2931, 2876, 1666, 1556, 1368, 1149, 1096, 968 cm^−1^; ^1^H NMR (400 MHz, CDCl_3_) Major *E*-**7a**-oxime δ 8.82 (1H, bs), 6.16 (1H, d, *J* = 3.1 Hz), 6.05 (1H, d, *J* = 3.1 Hz), 4.12 (1H, t, *J* = 7.0 Hz), 3.51 (2H, s), 3.32-3.51 (2H, m), 1.88 (3H, s), 1.74–1.91 (2H, m), 1.16 (3H, t, *J* = 7.0 Hz), 0.89 (3H, t, *J* = 7.4 Hz); characteristic signals of minor *Z*-**7a**-oxime δ 3.75 (2H, s), 1.82 (3H, s); ^13^C NMR (100 MHz, CDCl_3_) Major *E*-**7a**-oxime δ 155.3 (C_q_), 154.5 (C_q_), 150.0 (C_q_), 108.2 (CH), 107.6 (CH), 76.4 (CH), 64.0 (CH_2_), 34.8 (CH_2_), 27.3 (CH_2_), 15.2 (CH_3_), 13.1 (CH_3_), 10.1 (CH_3_); characteristic signals of minor *Z*-**7a**-oxime δ 154.9 (C_q_), 154.2 (C_q_), 149.6 (C_q_), 108.4 (CH), 107.7 (CH), 27.5 (CH_2_), 19.3 (CH_3_); HRMS (ESI^+^) *m*/*z* calcd for C_12_H_19_NNaO_3_ [M+Na]^+^: 248.1257, found 248.1263; calcd for C_12_H_19_KNO_3_ [M+K]^+^: 264.0997, found 264.0981.

**1-[5-(1-Ethoxy-2-methyl-propyl)-furan-2-yl]-propan-2-one oxime (7b) and 7-isobutylidene-3-methyl-1,6-dioxa-2-aza-spiro [4.4]nona-2,8-diene (6b).**

The crude product obtained from the ketone **5b** (280 mg, 1.43 mmol), NH_2_OH.HCl (397 mg, 5.71 mmol, 4 equivalent) and pyridine (0.46 mL, 5.71 mmol, 4 equivalent) was purified by column chromatography on silica gel (cyclohexane/EtOAc 99/1 to 90/10) to give a yellow oil (44 mg, 0.18 mmol, 13% yield) as an inseparable 77:15:8 mixture of *E*-**7b**:*Z*-**7b**:**6b**. *Rf* 0.61 (cyclohexane/EtOAc 6/4); IR (neat) ν 3253, 2962, 2929, 2873, 1674, 1559, 1473, 1387, 1372, 1238, 1156, 1093, 1015 cm^−1^; ^1^H NMR (400 MHz, CDCl_3_) Major oxime *E*-**7b** δ 8.46 (1H, bs), 6.14 (1H, d, *J* = 3.1 Hz), 6.05 (1H, d, *J* = 3.0 Hz), 3.81 (1H, d, *J* = 8.0 Hz), 3.51 (2H, s), 3.32 and 3.45 (2H, 2dq, *J* = 9.2, 7.0 Hz), 2.03–2.10 (1H, m), 1.87 (3H, s), 1.15 (3H, t, *J* = 7.0 Hz), 1.01 (3H, d, *J* = 6.6 Hz), 0.77 (3H, d, *J* = 6.6 Hz); characteristic signals of minor oxime *Z*-**7b** δ 3.75 and 3.76 (2H, 2s), 1.81 (3H, s); characteristic signals of bicyclic spiro compound 6b 6.27 (1H, d, *J* = 5.6 Hz), 5.92 (1H, d, *J* = 5.6 Hz), 4.44 (1H, d, *J* = 9.2 Hz), 3.13 (1H, dd, *J* = 18.1, 1.1 Hz), 3.02 (1H, d, *J* = 18.1 Hz), 2.75 (1H, d sept., *J* = 9.2, 6.7 Hz), 2.07 (3H, s); ^13^C NMR (100 MHz, CDCl_3_) Major oxime *E*-**7b** δ 155.4 (C_q_), 154.1 (C_q_), 149.9 (C_q_), 108.7 (CH), 107.6 (CH), 80.9 (CH), 64.4 (CH_2_), 34.8 (CH_2_), 32.5 (CH), 19.1 (CH_3_), 18.7 (CH_3_), 15.1 (CH_3_), 13.0 (CH_3_); characteristic signals of minor oxime *Z*-**7b** δ 155.0 (C_q_), 153.9 (C_q_), 149.5 (C_q_), 108.9 (CH), 107.7 (CH), 27.5 (CH_2_), 19.3 (CH_3_); characteristic signals of bicyclic spiro compound **6b** 156.6 (C_q_), 130.1 (CH), 126.5 (CH), 118.4 (C_q_), 111.3 (CH), 46.7 (CH_2_), 25.3 (CH), 13.4 (CH_3_). HRMS of oximes 7b (ESI^+^) *m*/*z* calcd for C_13_H_21_NNaO_3_ [M+Na]^+^: 262.1414, found 262.1408.

**1-[5-(1-Ethoxy-3-methyl-butyl)-furan-2-yl]-propan-2-one oxime (7c).**

The crude product obtained from the ketone **5c** (272.5 mg, 1.3 mmol), NH_2_OH.HCl (361 mg, 5.2 mmol, 4 equivalent) and pyridine (0.42 mL, 5.2 mmol, 4 equivalent), giving a mixture of **7c** and 6c, was purified by column chromatography on silica gel (PE/EtOAc 85/15) to give a yellow oil (45.7 mg, 0.18 mmol, 14% yield) as an inseparable 87:13 mixture of *E* and *Z* oximes **7c**. *Rf* 0.49 (PE/EtOAc 7/3); IR (neat) ν 3320, 2959, 2929, 2873, 1469, 1369, 1171, 1089 cm^−1^; ^1^H NMR (400 MHz, CDCl_3_) Major oxime **7c** δ 8.56 (1H, bs), 6.16 (1H, d, *J* = 3.1 Hz), 6.05 (1H, d, *J* = 3.1 Hz), 4.27 (1H, dd, *J* = 8.0, 6.1 Hz), 3.51 (2H, s), 3.45 (1H, dq, *J* = 9.2, 7.0 Hz), 3.36 (1H, dq, *J* = 9.2, 7.0 Hz), 1.88 (3H, s), 1.75–1.84 (1H, m), 1.55–1.71 (2H, m), 1.15 (3H, t, *J* = 7.0 Hz), 0.91 (3H, d, *J* = 6.5 Hz), 0.89 (3H, d, *J* = 6.5 Hz); characteristic signals of minor oxime **7c** δ 3.75 and 3.76 (2H, 2s), 1.82 (3H, s); ^13^C NMR (100 MHz, CDCl_3_) δ 155.3 (C_q_), 154.8 (C_q_), 150.0 (C_q_), 108.1 (CH), 107.6 (CH), 73.1 (CH), 63.9 (CH_2_), 43.2 (CH_2_), 34.8 (CH_2_), 24.6 (CH), 22.8 (CH_3_CH), 22.3 (CH_3_), 15.2 (CH_3_), 13.1 (CH_3_); HRMS (ESI^+^) *m*/*z* calcd for C_14_H_23_NNaO_3_ [M+Na]^+^: 276.1576, found 276.1570.

**3-Methyl-7-(3-methyl-butylidene)-1,6-dioxa-2-aza-spiro [4.4]nona-2,8-diene (6c).**

Compound 6c was obtained in the same experiment as **7c** and purified by column chromatography on silica gel (CH/EtOAc 90/10) to give a yellow oil (37.7 mg, 0.18 mmol, 14% yield). *Rf* 0.47 (PE/EtOAc 7/3); ^1^H NMR (400 MHz, CDCl_3_) δ 6.31 (1H, d, *J* = 5.5 Hz), 5.94 (1H, d, *J* = 5.5 Hz), 4.60 (1H, t, *J* = 7.7 Hz), 3.14 and 3.02 (2H, 2d, *J* = 18.2 Hz), 2.08 (3H, s), 2.04–2.06 (2H, m), 1.55–1.68 (1H, m), 0.90 (3H, d, *J* = 6.7 Hz), 0.89 (3H, d, *J* = 6.6 Hz); ^13^C NMR (100 MHz, CDCl_3_) δ 156.6 (C_q_), 155.4 (C_q_), 129.9 (CH), 126.5 (CH), 118.4 (C_q_), 102.7 (CH), 46.7 (CH_2_), 34.4 (CH_2_), 28.7 (CH), 22.3 (CH_3_), 22.2 (CH_3_), 13.4 (CH_3_); HRMS (ESI^+^) *m*/*z* calcd for C_12_H_17_NNaO_2_ [M–H_2_O+Na]^+^: 230.1151, found 230.1142.

**1-[5-(1-Ethoxy-pent-4-enyl)-furan-2-yl]-propan-2-one oxime (7d) and 3-Methyl-7-pent-4-enylidene-1,6-dioxa-2-aza-spiro [4.4]nona-2,8-diene (6d).**

The crude product obtained from the ketone **5d** (394 mg, 1.89 mmol), NH_2_OH.HCl (526 mg, 7.58 mmol, 4 equivalent) and pyridine (0.61 mL, 7.58 mmol, 4 equivalent) was purified by column chromatography on silica gel (cyclohexane/EtOAc 99/1) to give a yellow oil (62 mg, 0.25 mmol, 13% yield) as an inseparable 65:26:9 mixture of *E*-**7d**:*Z*-7d:6d. *Rf* 0.55 (cyclohexane/EtOAc 6/4); IR (neat) ν 3268, 3082, 2977, 2925, 2858, 1644, 1569, 1443, 1372, 1335, 1160, 1067, 1089 cm^−1^; ^1^H NMR (400 MHz, CDCl_3_) Major oxime *E*-**7d** δ 8.54 (1H, bs), 6.16 (1H, d, *J* = 3.1 Hz), 6.05 (1H, d, *J* = 3.1 Hz), 5.80 (1H, ddt, *J* = 17.3, 10.3, 6.3 Hz), 5.01 (1H, dq, *J* = 17.3, 1.7 Hz), 4.96 (1H, dq, *J* = 10.4, 1.5 Hz), 4.22 (1H, t, *J* = 7.0 Hz), 3.51 (2H, s), 3.45 (1H, dq, *J* = 9.3, 7.0 Hz), 3.36 (1H, dq, *J* = 9.3, 7.0 Hz), 2.03–2.16 (1H, m), 1.80–2.03 (1H, m), 1.88 (3H, s), 1.60 (3H, t, *J* = 7.0 Hz); characteristic signals of minor oxime *Z*-7d δ 3.75 (2H, d, *J* = 1.8 Hz), 1.82 (3H, s); characteristic signals of bicyclic spiro compound **6d** δ 6.30 (1H, d, *J* = 5.6 Hz), 5.95 (1H, d, *J* = 5.6 Hz), 4.58 (1H, t, *J* = 7.4 Hz), 3.15 (1H, d, *J* = 18.1 Hz), 3.03 (1H, d, *J* = 18.1 Hz), ^13^C NMR (100 MHz, CDCl_3_) Major oxime *E*-**7d** δ 155.3 (C_q_), 154.4 (C_q_), 150.2 (C_q_), 138.0 (CH), 114.9 (CH_2_), 108.3 (CH), 107.7 (CH), 74.1 (CH), 64.1 (CH_2_), 34.8 (CH_2_), 33.4 (CH_2_), 29.8 (CH_2_), 15.2 (CH_3_), 13.1 (CH_3_); characteristic signals of minor oxime *Z*-7d δ 154.9 (C_q_), 154.1 (C_q_), 149.7 (C_q_), 138.0 (CH), 108.4 (CH), 107.7 (CH), 74.1 (CH), 64.0 (CH_2_), 27.5 (CH_2_); characteristic signals of bicyclic spiro compound 6d δ 129.8 (CH), 126.8 (CH), 102.8 (CH), 46.7 (CH_2_); HRMS of oximes **7d** (ESI^+^) *m*/*z* calcd for C_14_H_21_NNaO_3_ [M+Na]^+^: 274.1414, found 274.1417; HRMS of bicyclic spiro compound 6d (ESI^+^) *m*/*z* calcd for C_12_H_16_NO_2_ [M+H]^+^: 206.1176, found 206.1178.

**7-Benzylidene-3-methyl-1,6-dioxa-2-aza-spiro [4.4] nona-2,8-diene (6e).**

The crude product obtained from the ketone **5e** (500 mg, 2.17 mmol), NH_2_OH.HCl (604 mg, 8.69 mmol, 4 equivalent) and pyridine (0.7 mL, 8.7 mmol, 4 equivalent) was purified by column chromatography on silica gel (cyclohexane/EtOAc 90/10) to give a white solid (354 mg, 1.56 mmol, 72% yield). *Rf* 0.41 (cyclohexane/EtOAc 6/4); mp 113.5–115.0 °C; IR (neat) ν 3104, 3052, 2985, 2925, 2858, 1652, 1599, 1391, 1369, 1339, 1179, 1108, 940, 844, 825 cm^−1^; ^1^H NMR (400 MHz, CDCl_3_) δ 7.60 (2H, dd, *J* = 7.2, 1.3 Hz), 7.29 (2H, t, *J* = 7.5 Hz), 7.16 (1H, tt, *J* = 7.3, 1.2 Hz), 6.45 (1H, d, *J* = 5.5 Hz), 6.08 (1H, dd, *J* = 5.5, 0.7 Hz), 5.51 (1H, s), 3.25 (1H, dq, *J* = 18.3, 1.1 Hz), 3.13 (1H, dq, *J* = 18.3, 0.6 Hz), 2.11 (3H, s); ^13^C NMR (100 MHz, CDCl_3_) δ 156.9 (C_q_), 155.3 (C_q_), 135.2 (C_q_), 131.2 (CH), 128.5 (2CH), 128.3 (2CH), 127.5 (CH), 126.3 (CH), 119.9 (C_q_), 103.3 (CH), 47.0 (CH_2_), 13.4 (CH_3_); HRMS (ESI^+^) *m*/*z* calcd for C_14_H_14_NO_2_ [M+H]^+^: 228.1025, found 228.1019; *m*/*z* calcd for C_14_H_13_NNaO_2_ [M+Na]^+^: 250.0844, found 250.0847.

**7-Benzylidene-3-phenoxymethyl-1,6-dioxa-2-aza-spiro [4.4]nona-2,8-diene (6f).**

The crude product obtained from the ketone **5f** (191 mg, 0.59 mmol), NH_2_OH.HCl (164.7 mg, 2.37 mmol, 4 equivalent) and pyridine (0.2 mL, 2.4 mmol, 4 equivalent) was purified by column chromatography on silica gel (cyclohexane/EtOAc 7/3) to give a colorless oil (118 mg, 0.369 mmol, 62% yield). *Rf* 0.52 (cyclohexane/EtOAc 7/3); ^1^H NMR (400 MHz, CDCl_3_) δ 7.55 (2H, dd, *J* = 7.9, 0.7 Hz), 7.25-7.35 (4H, m), 7.16 (1H, t, *J* = 7.4 Hz), 7.04 (1H, t, *J* = 7.4 Hz), 6.99 (2H, dd, *J* = 8.7, 0.9 Hz), 6.48 (1H, d, *J* = 5.5 Hz), 6.09 (1H, dd, *J* = 5.5, 0.7 Hz), 5.53 (1H, s), 4.97 and 4.93 (2H, 2d, *J* = 13.0 Hz), 3.349 and 3.347 (2H, 2d, *J* = 18.5 Hz); ^13^C NMR (100 MHz, CDCl_3_) δ 157.7 (C_q_), 157.5 (C_q_), 155.1 (C_q_), 135.0 (C_q_), 131.6 (CH), 129.7 (2CH), 128.6 (2CH), 128.3 (2CH), 127.0 (CH), 126.5 (CH), 121.9 (CH), 120.2 (C_q_), 114.8 (2CH), 103.9 (CH), 62.9 (CH_2_), 43.6 (CH_2_); HRMS (ESI^+^) *m*/*z* calcd for C_20_H_18_NO_3_ [M–H_2_O+H]^+^: 320.1281, found 320.1280; calcd for C_20_H_18_NNaO_3_ [M+Na]^+^: 342.1101, found 342.1103.

## 4. Conclusions

Oximation of ketofurfuryl alcohols in ethanol with hydroxylamine hydrochloride in the presence of pyridine led, in most cases, to an oxime along with a spiroisoxazoline. The oxime/spiroisoxazoline product ratio depended mainly on the nature of the substituent attached to the furfuryl alcohol moiety (i.e., R_1_). If the substituent was a phenyl group, only the spiroisoxazoline presenting a dihydrofurane moiety including a phenyl methylene group was obtained (yield up to 72%), suggesting an important stabilisation by a mesomeric effect. The suggested formation of spiranic derivatives involved an in situ oximation/dehydration/S_N_’ cascade reaction.

This original pathway, giving access to new pharmacophores, prompted us to further study rearrangement from furyloximes.

## Figures and Tables

**Table 1:** *Tert*-butyldimethylsilyl ether deprotection.

Entry	Substrate 4			Conditions *	Product 5	Yield %
		R_1_	R_2_			
1	**4a**	Et	Me	A	**5a**	40
2	**4b**	iPr	Me	A	**5b**	14
3				B		64
4	**4c**	iBu	Me	A	**5c**	61
5	**4d**	3-Butenyl	Me	A	**5d**	Degradation
6				B		59
7	**4e**	Ph	Me	A	**5e**	87
8	**4f**	Ph	CH_2_OPh	A	**5f**	16
9				B		86

* Condition A: 1.5 eq. TBAF, THF, rt, 2 h; Condition B: 1.5 eq. Et_3_N·3HF, CH_3_CN, rt, 16 h.

**Table 2:** Oximation of ketone **5**.

Entry	Substrate 5			Product 6 (Yield %)	Product 7 (Yield %)
		R_1_	R_2_		
1	**5a**	Et	Me	**6a** (4)	**7a** (34)
2	**5b**	iPr	Me	**6b** (1)	**7b** (12)
3	**5c**	iBu	Me	**6c** (14)	**7c** (14)
4	**5d**	3-Butenyl	Me	**6d** (16)	**7d** (32)
5	**5e**	Ph	Me	**6e** (61)	**7e** (0)
6	**5f**	Ph	CH_2_OPh	**6f** (72)	**7f** (0)

## Data Availability

Data are contained within the article and Supplementary Materials.

## Supplementary Materials

The following supporting information can be downloaded at: https://www.mdpi.com/article/10.3390/molecules29225474/s1, Figures S1–S10: NMR spectra of silylated alcohols **2a**–**e**; Figures S11–S22: NMR spectra of compounds **3a**–**f**; Figures S23–S34: NMR spectra of compounds **4a**–**f**; Figures S35–S46: NMR spectra of keto alcohols **5a**–**f**; Figures S47–S54: NMR spectra of compounds **7a**–**d**; Figures S55–S67 NMR spectra of compounds **6c**,**e** and **f**.
