# Supplementary material for: An Unexpected Formation of Spiro Isoxazoline-Dihydrofurane Compounds from Substituted Ketofurfuryl Alcohols
Source: Molecules. 2024 Nov 20;29(22):5474. doi: 10.3390/molecules29225474 (PMC11597928; doi:10.3390/molecules29225474)
Supplement: Supplementary file 1 [file molecules-29-05474-s001.zip › molecules-3272959-supplementary.pdf]

An unexpected formation of spiro isoxazoline-dihydrofuran compounds from substituted ketofurfuryl alcohols

Claire Cuyamendous, Mathieu Y. Laurent, Christine Saluzzo

MSO, Institut des Molécules et Matériaux du Mans (IMMM), UMR CNRS 6283,

Le Mans Université, Avenue O. Messiaen, 72085 Le Mans, CEDEX 9, France

Content

1) NMR spectra of silylated alcohols **2a-e**..S2

2) NMR spectra of compounds **3a-f**..S7

3) NMR spectra of compounds **4a-f**..S13

4) NMR spectra of keto alcohols **5a-f**...S19

5) NMR spectra of compounds **7a-d**..S25

6) NMR spectra of compounds **6c,e-f**...S29

1) NMR spectra of silylated alcohols **2a-e**

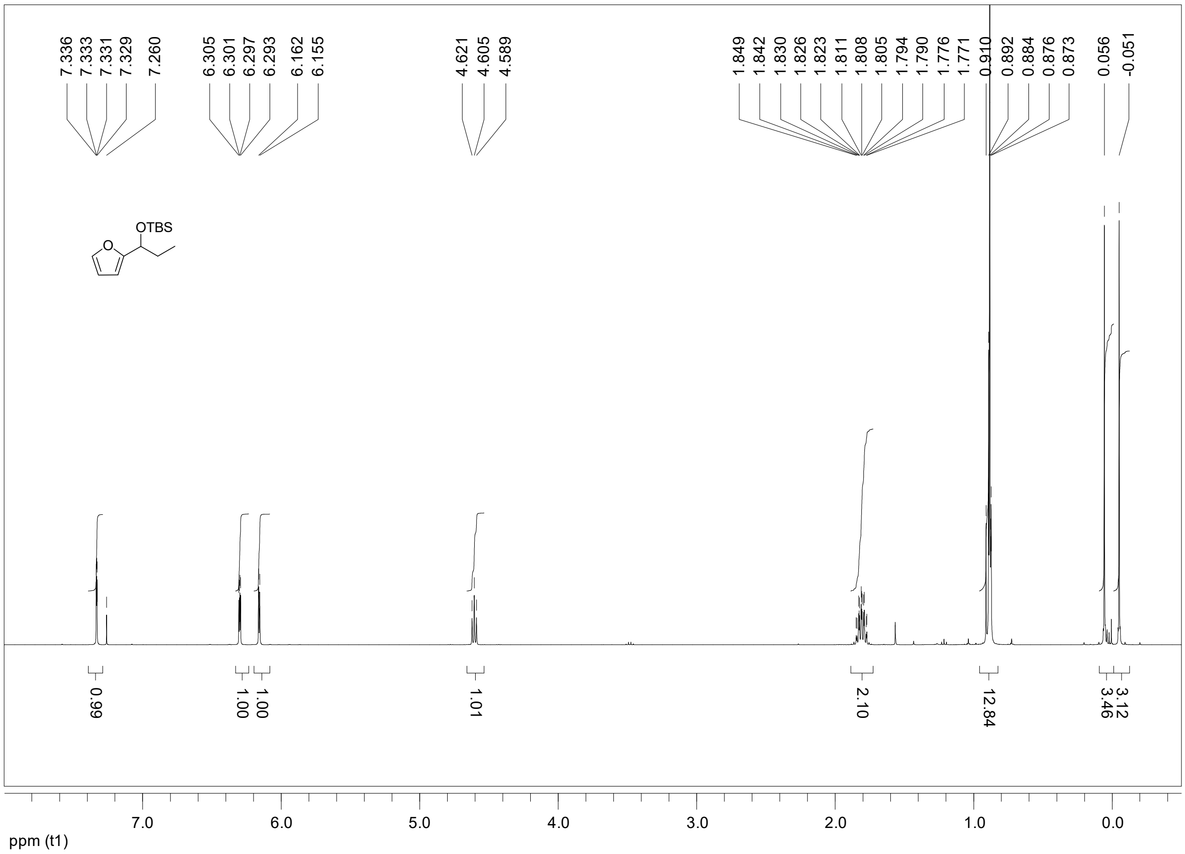

Figure S1: ^{1}H NMR spectrum (CDCl$_3$, 400 MHz) of *tert*-Butyl-(1-furan-2-yl-propoxy)-dimethyl-silane (2a)

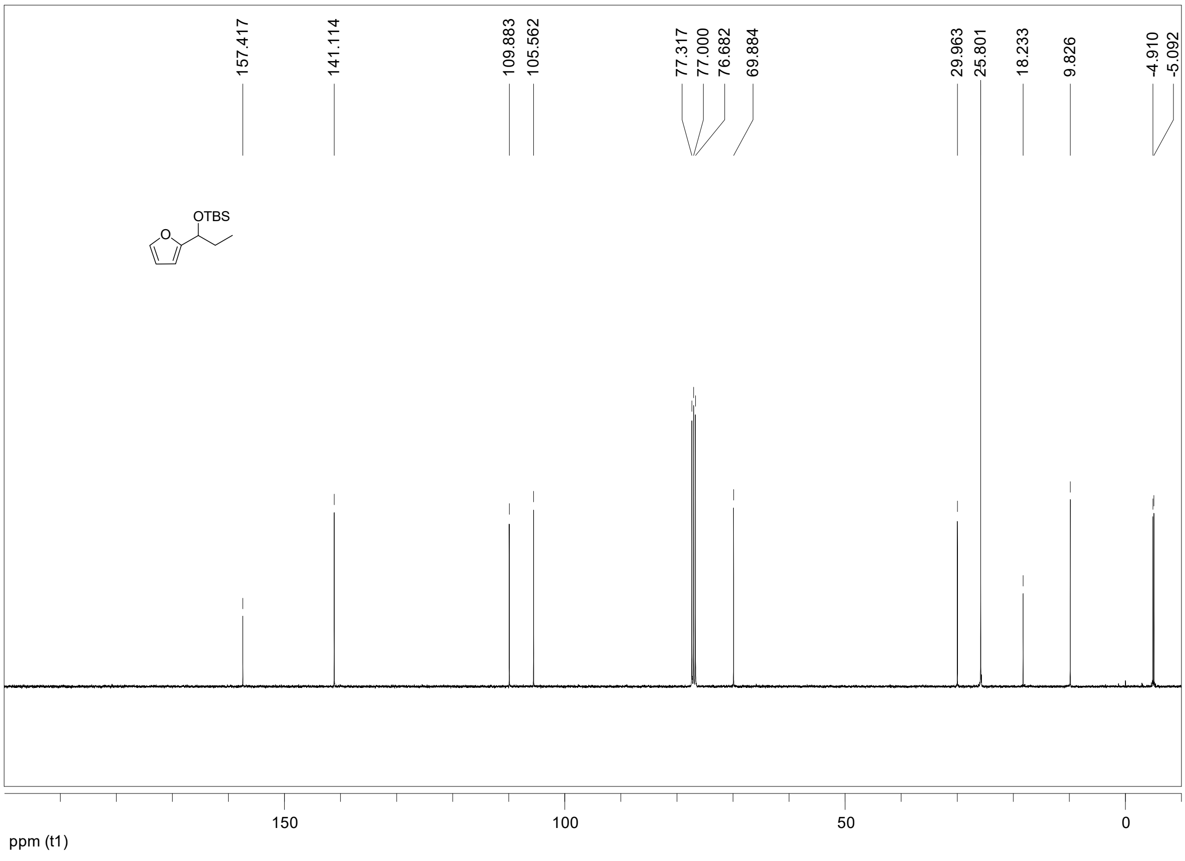

Figure S2: ^{13}C{^{1}H} NMR spectrum (CDCl$_3$, 100 MHz) of *tert*-Butyl-(1-furan-2-yl-propoxy)-dimethyl-silane (2a)

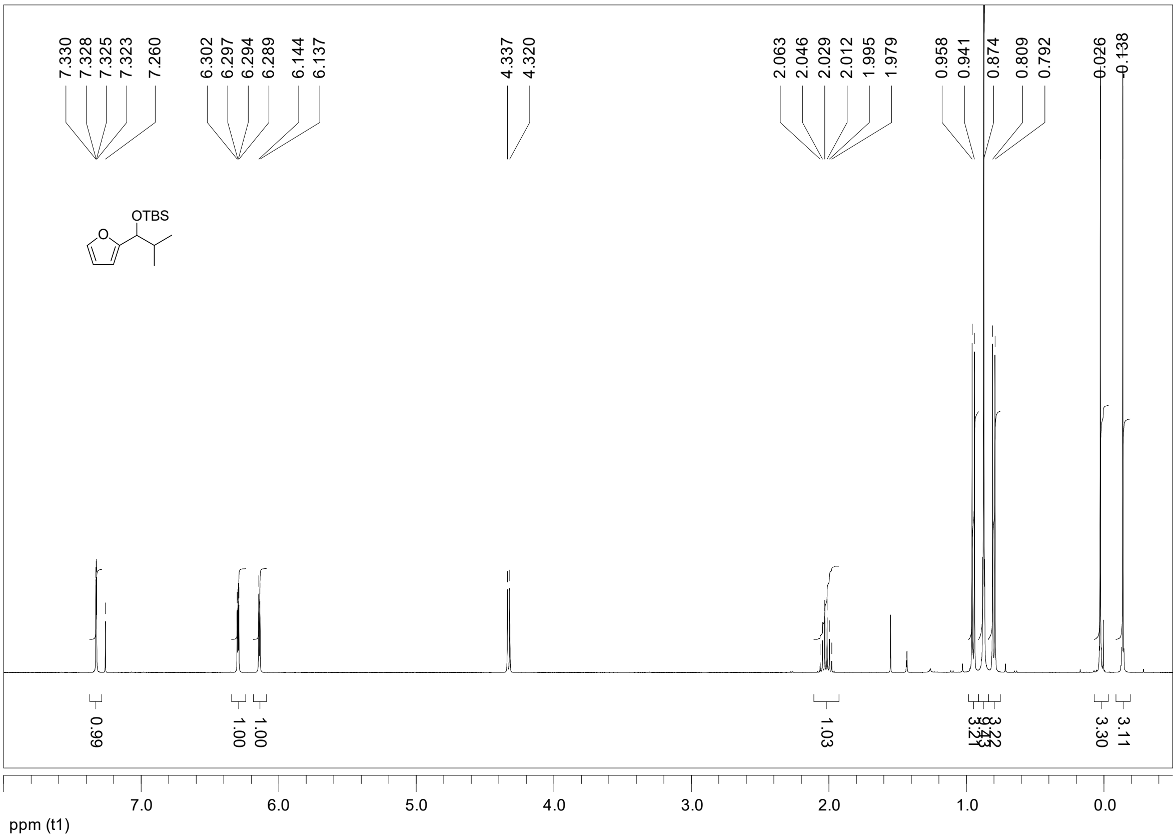

Figure S3: ^{1}H NMR spectrum ($CDCl_3$, 400 MHz) of *tert*-Butyl-(1-furan-2-yl-2-methyl-propoxy)-dimethyl-silane (2b)

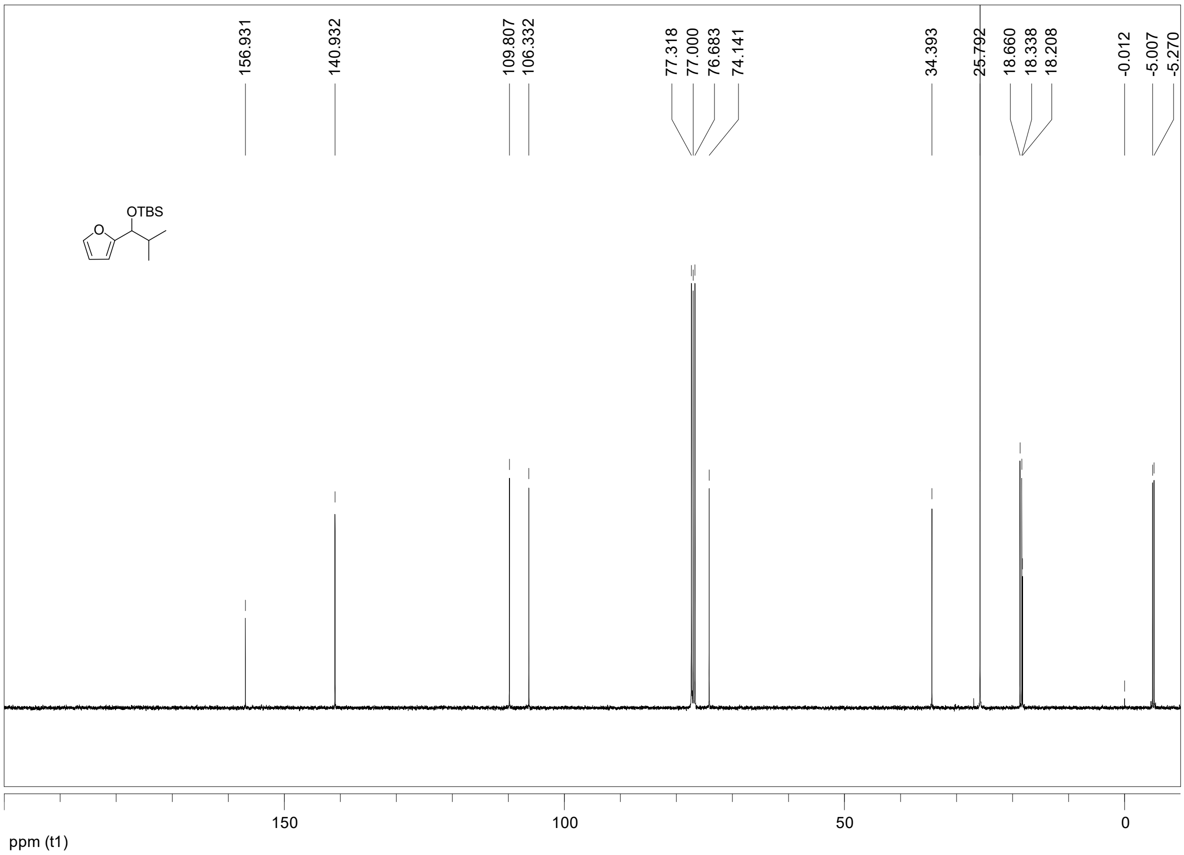

Figure S4: $^{13}C\{^{1}H\}$ NMR spectrum ($CDCl_3$, 100 MHz) of *tert*-Butyl-(1-furan-2-yl-2-methyl-propoxy)-dimethyl-silane (2b)

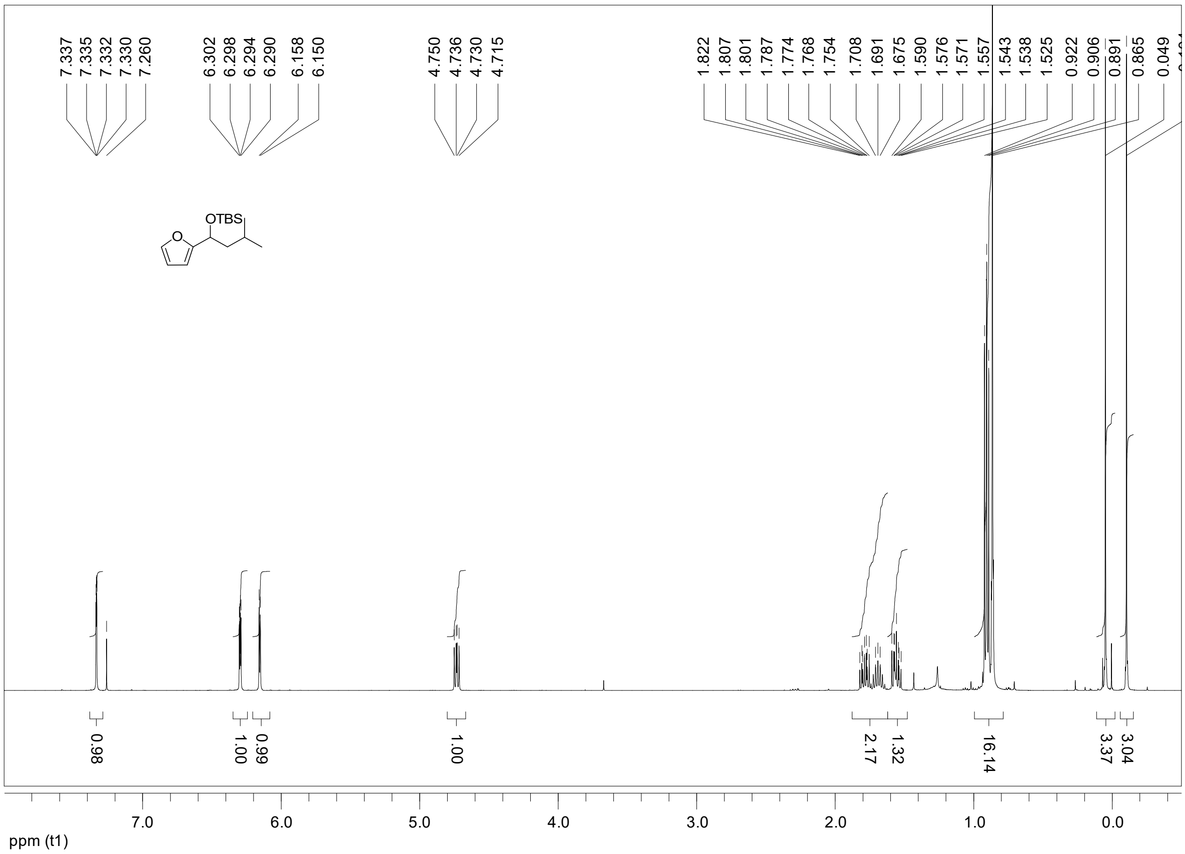

Figure S5: ^{1}H NMR spectrum ($CDCl_3$, 400 MHz) of *tert*-Butyl-(1-furan-2-yl-3-methyl-butoxy)-dimethyl-silane (2c)

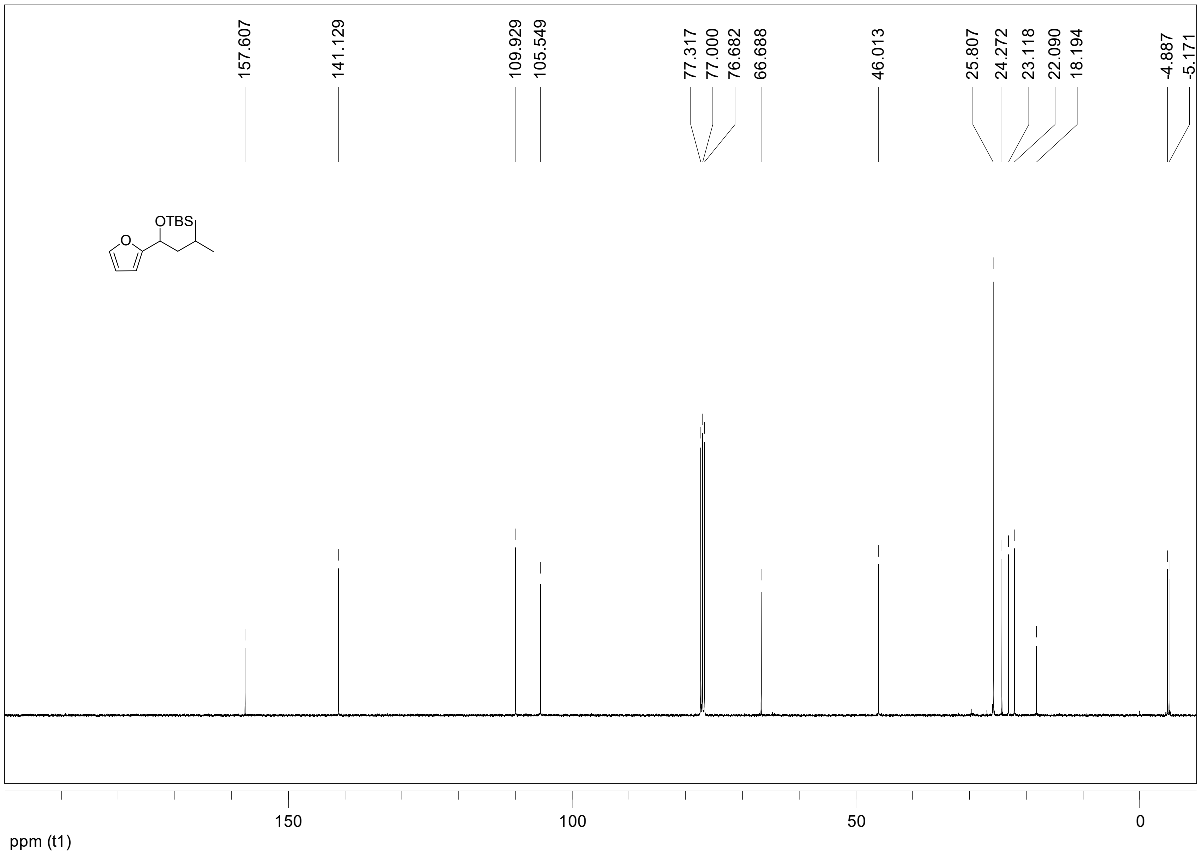

Figure S6: $^{13}C\{^1H\}$ NMR spectrum ($CDCl_3$, 100 MHz) of *tert*-Butyl-(1-furan-2-yl-3-methyl-butoxy)-dimethyl-silane (2c)

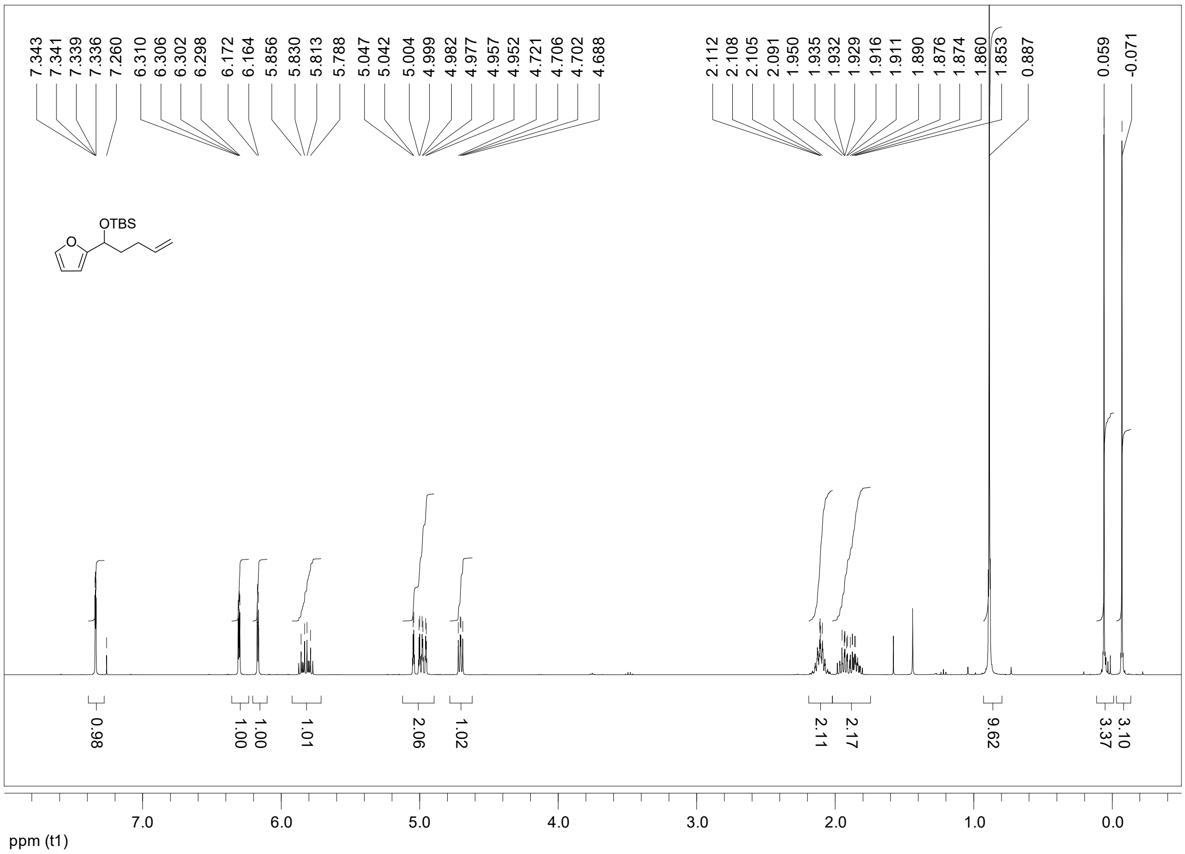

Figure S7: ^{1}H NMR spectrum ($CDCl_3$, 400 MHz) of *tert*-Butyl-(1-furan-2-yl-pent-4-enyloxy)-dimethyl-silane (2d)

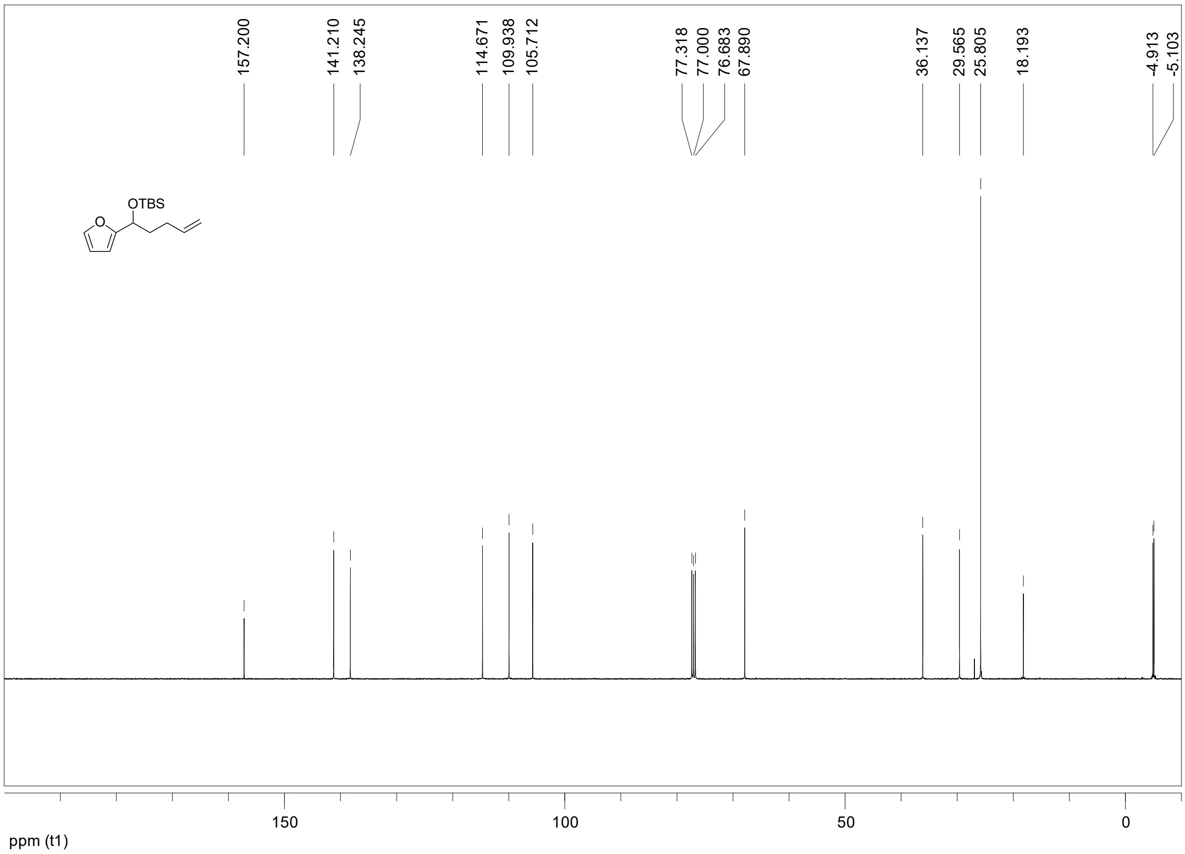

Figure S8: $^{13}C\{^{1}H\}$ NMR spectrum ($CDCl_3$, 100 MHz) of *tert*-Butyl-(1-furan-2-yl-pent-4-enyloxy)-dimethyl-silane (2d)

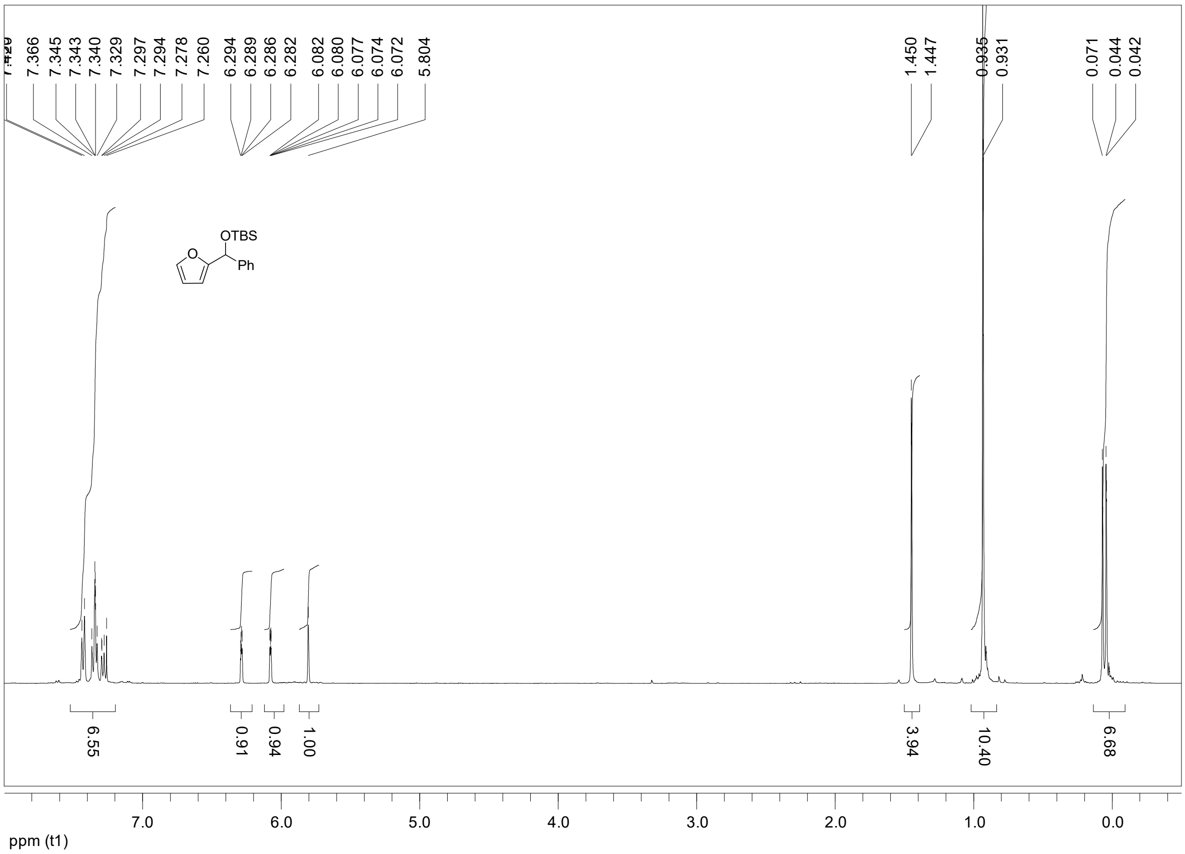

Figure S9: ^{1}H NMR spectrum ($CDCl_3$, 400 MHz) of *tert*-Butyl-(furan-2-yl-phenyl-methoxy)-dimethyl-silane (2e)

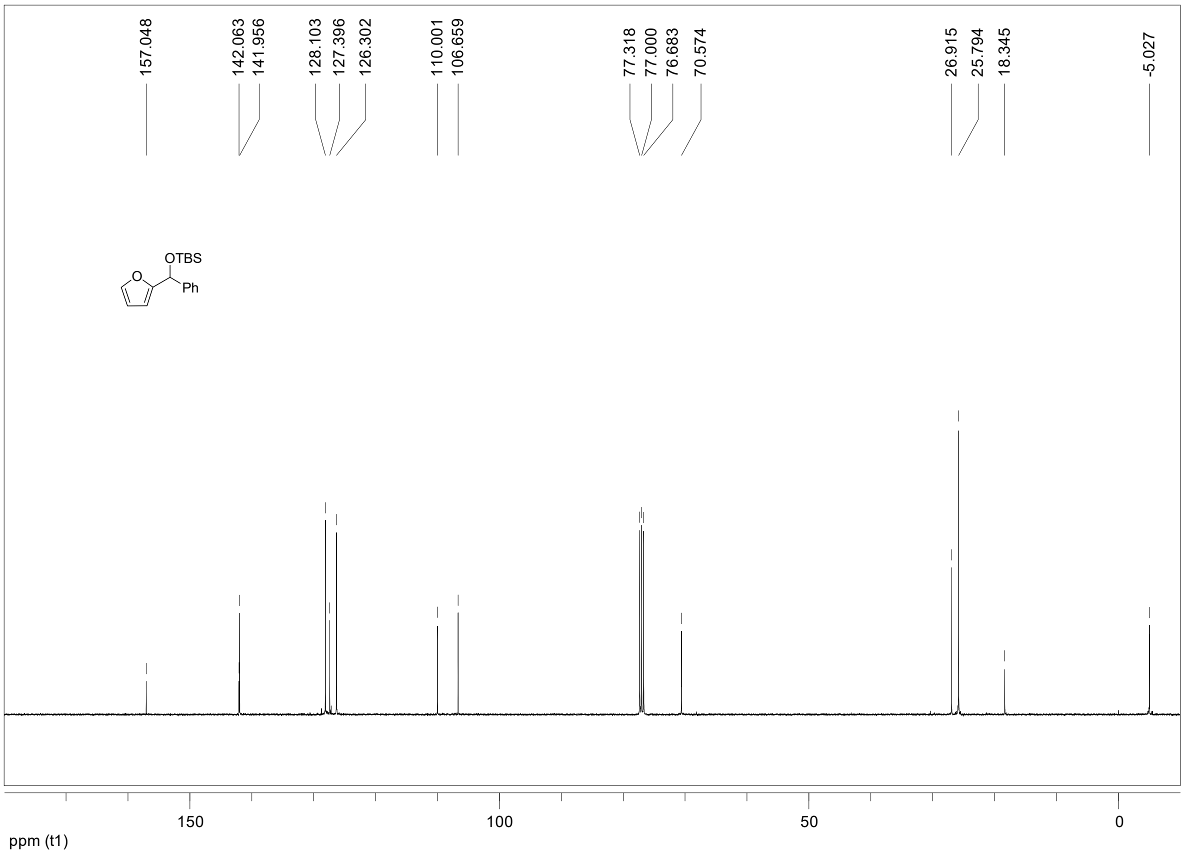

Figure S10: $^{13}C\{^{1}H\}$ NMR spectrum ($CDCl_3$, 100 MHz) of *tert*-Butyl-(furan-2-yl-phenyl-methoxy)-dimethyl-silane (2e)

2) NMR spectra of compounds **3a-f**

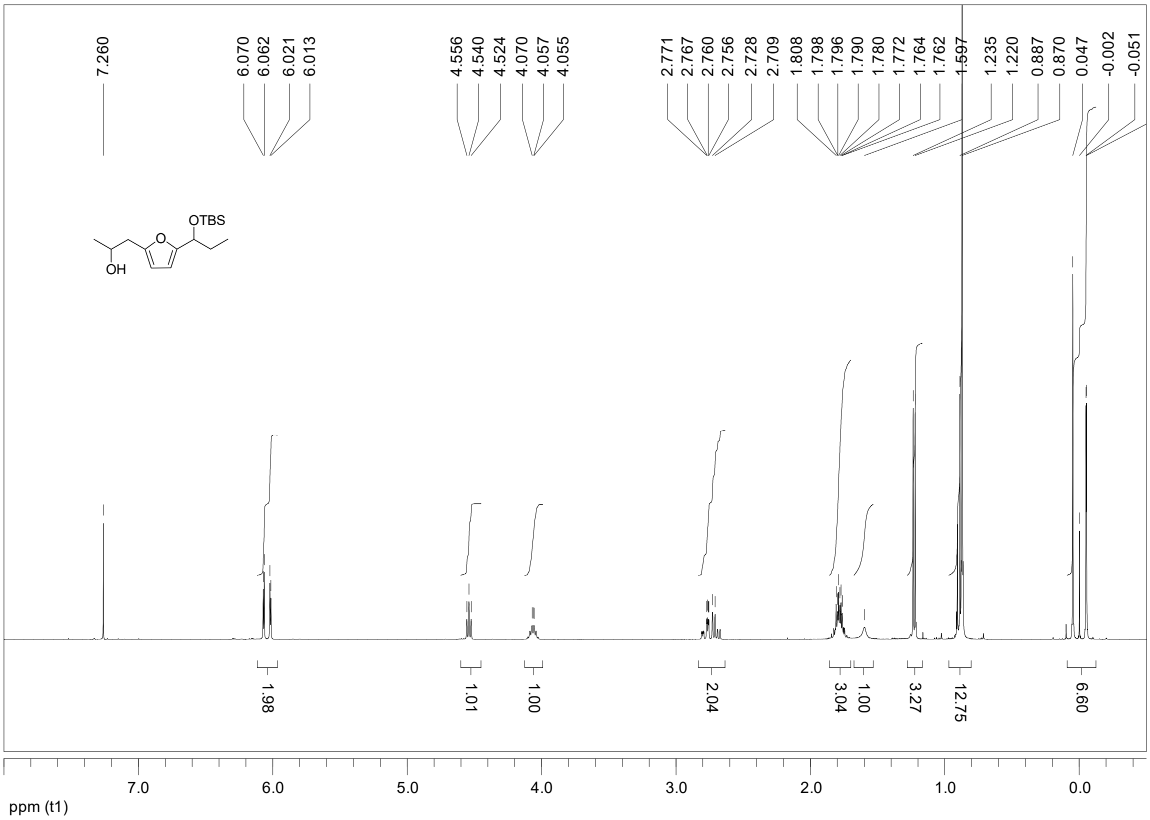

Figure S11: ^{1}H NMR spectrum ($CDCl_3$, 400 MHz) of 1-[5-[1-(*tert*-Butyl-dimethyl-silanyloxy)-propyl]-furan-2-yl]-propan-2-ol (3a)

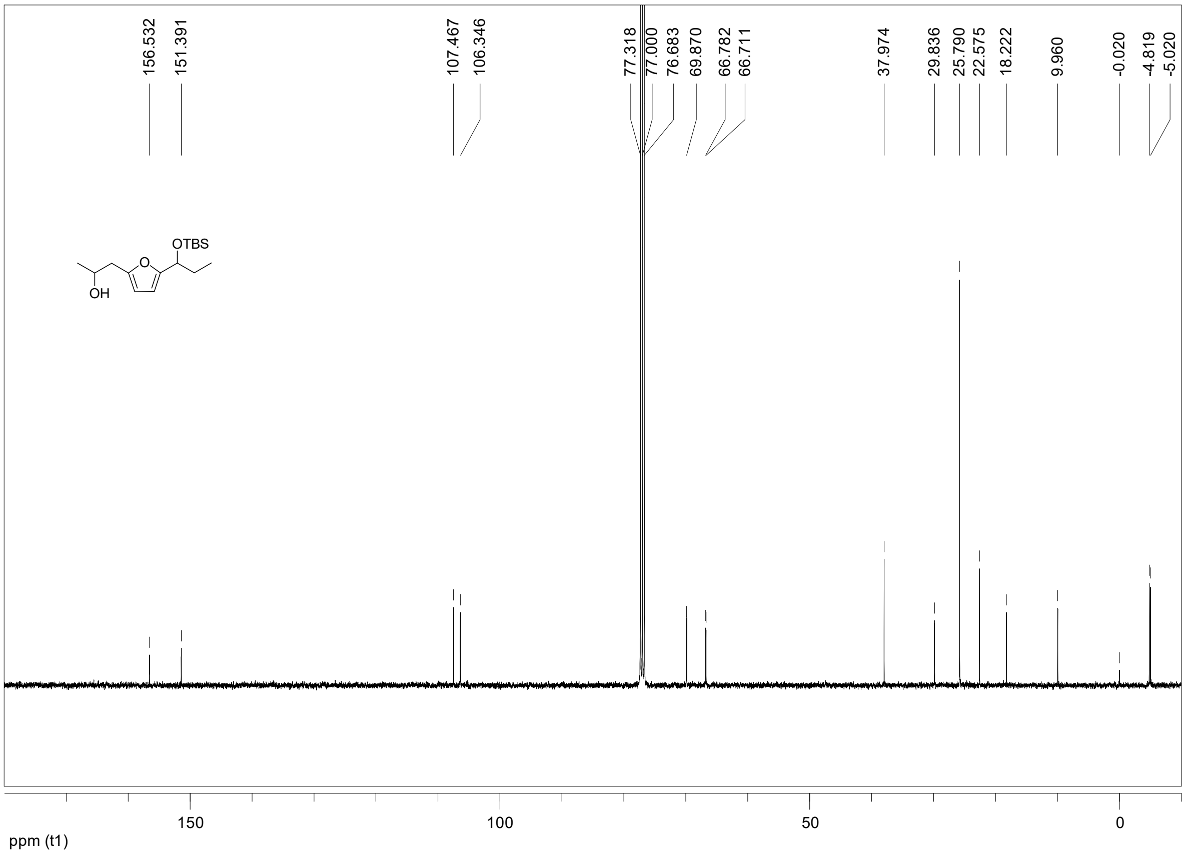

Figure S12: $^{13}C\{^{1}H\}$ NMR spectrum ($CDCl_3$, 100 MHz) of 1-[5-[1-(*tert*-Butyl-dimethyl-silanyloxy)-propyl]-furan-2-yl]-propan-2-ol (3a)

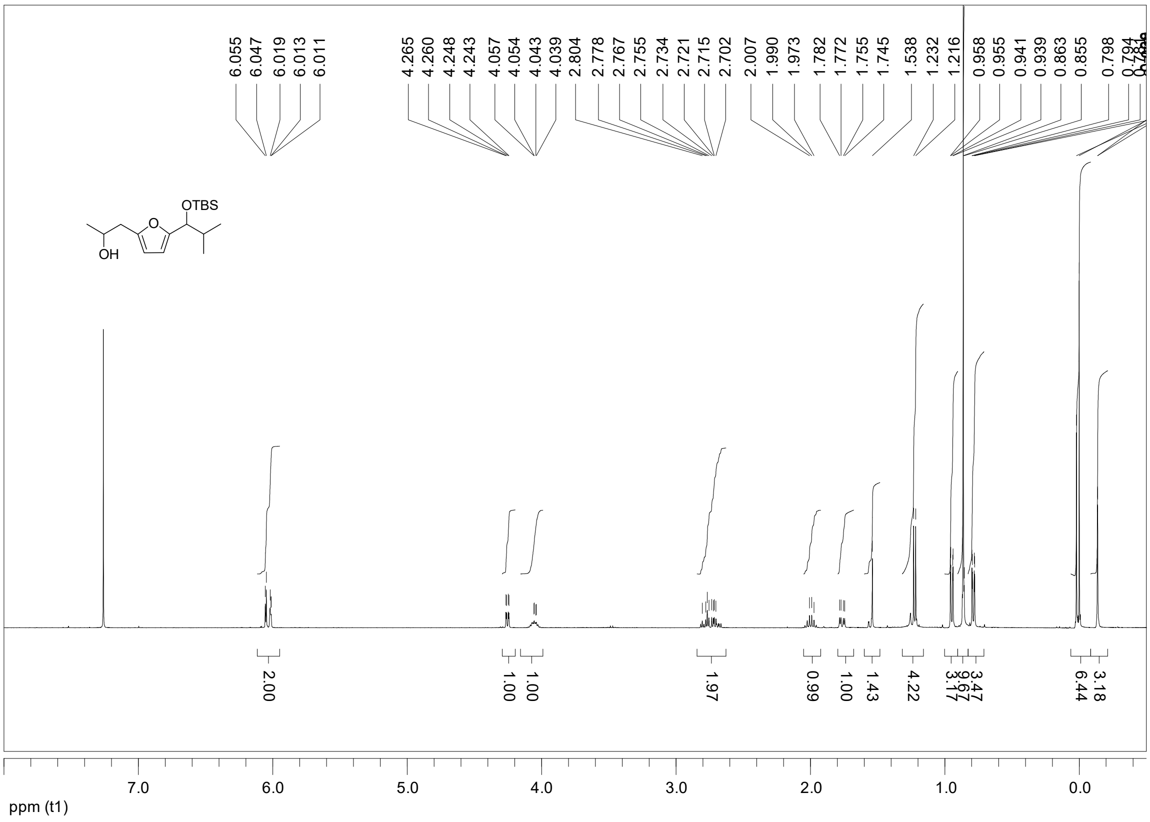

Figure S13: ^{1}H NMR spectrum ($CDCl_3$, 400 MHz) of 1-[5-[1-(tert-Butyl-dimethyl-silanyloxy)-2-methyl-propyl]-furan-2-yl]-propan-2-ol (3b)

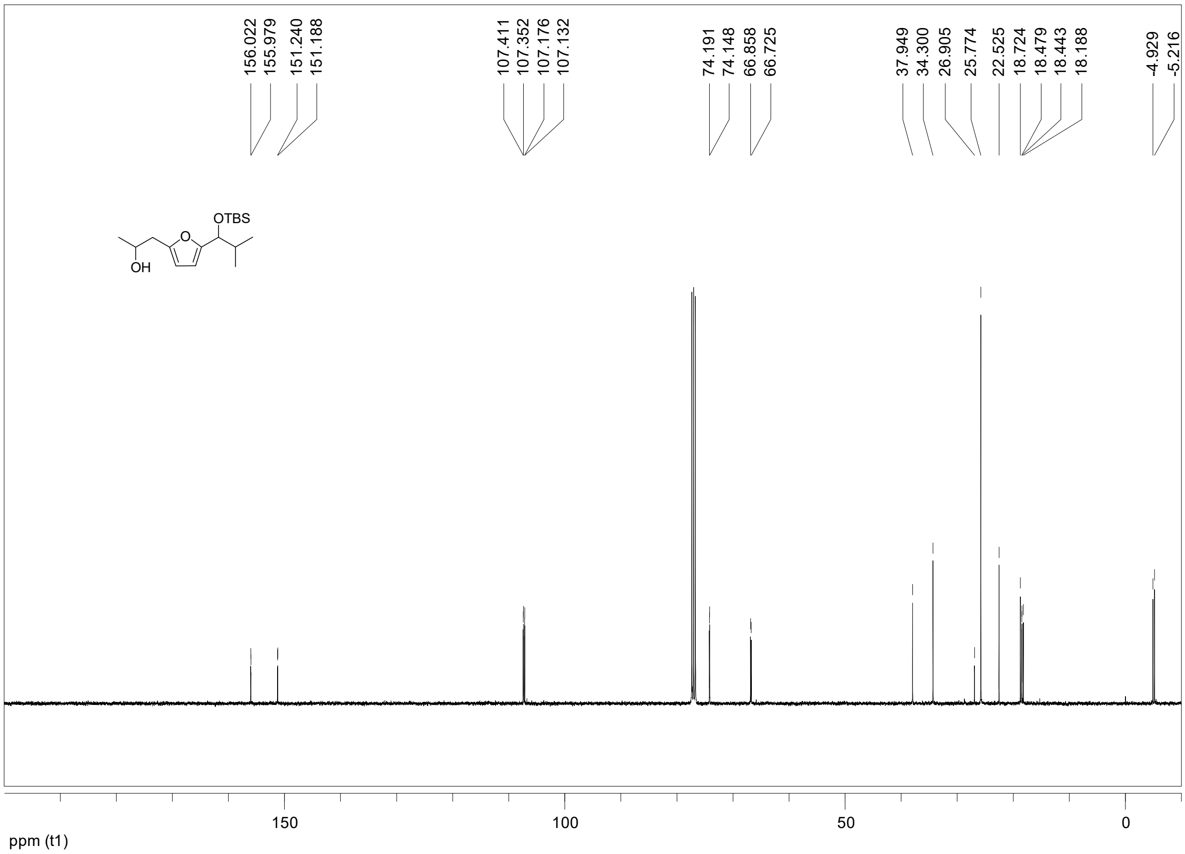

Figure S14: $^{13}C\{^{1}H\}$ NMR spectrum ($CDCl_3$, 100 MHz) of 1-[5-[1-(tert-Butyl-dimethyl-silanyloxy)-2-methyl-propyl]-furan-2-yl]-propan-2-ol (3b)

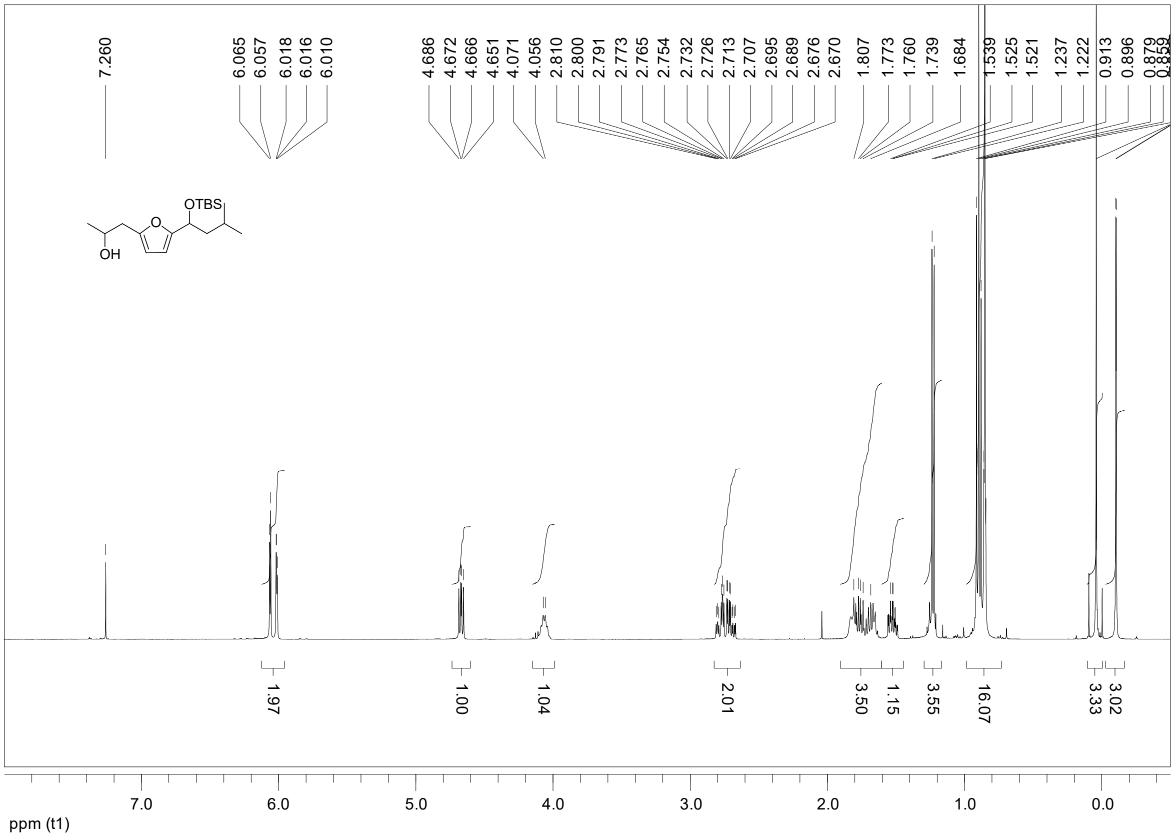

Figure S15: ^{1}H NMR spectrum ($CDCl_3$, 400 MHz) of 1-[5-[1-(*tert*-Butyl-dimethyl-silanyloxy)-3-methyl-butyl]-furan-2-yl]-propan-2-ol (3c)

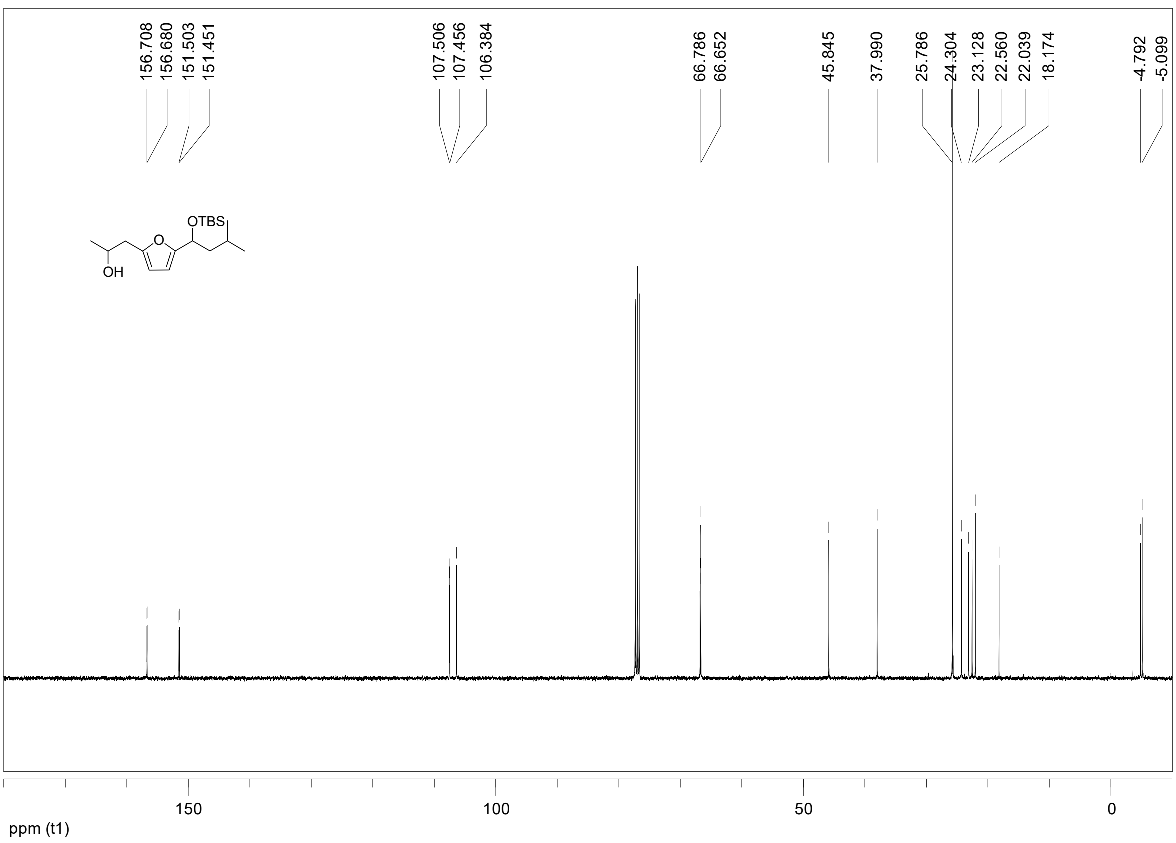

Figure S16: $^{13}C\{^{1}H\}$ NMR spectrum ($CDCl_3$, 100 MHz) of 1-[5-[1-(*tert*-Butyl-dimethyl-silanyloxy)-3-methyl-butyl]-furan-2-yl]-propan-2-ol (3c)

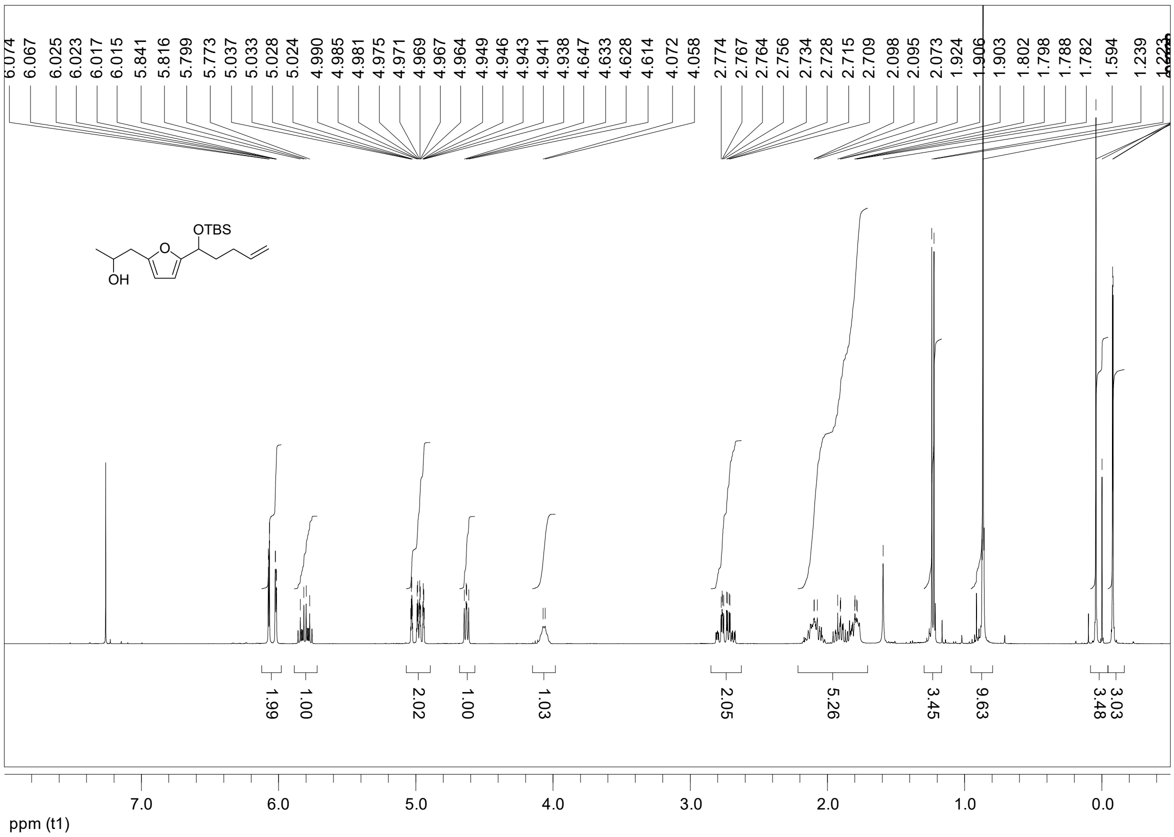

Figure S17: ^{1}H NMR spectrum ($CDCl_3$, 400 MHz) of 1-[5-[1-(*tert*-Butyl-dimethyl-silanyloxy)-pent-4-enyl]-furan-2-yl]-propan-2-ol (3d)

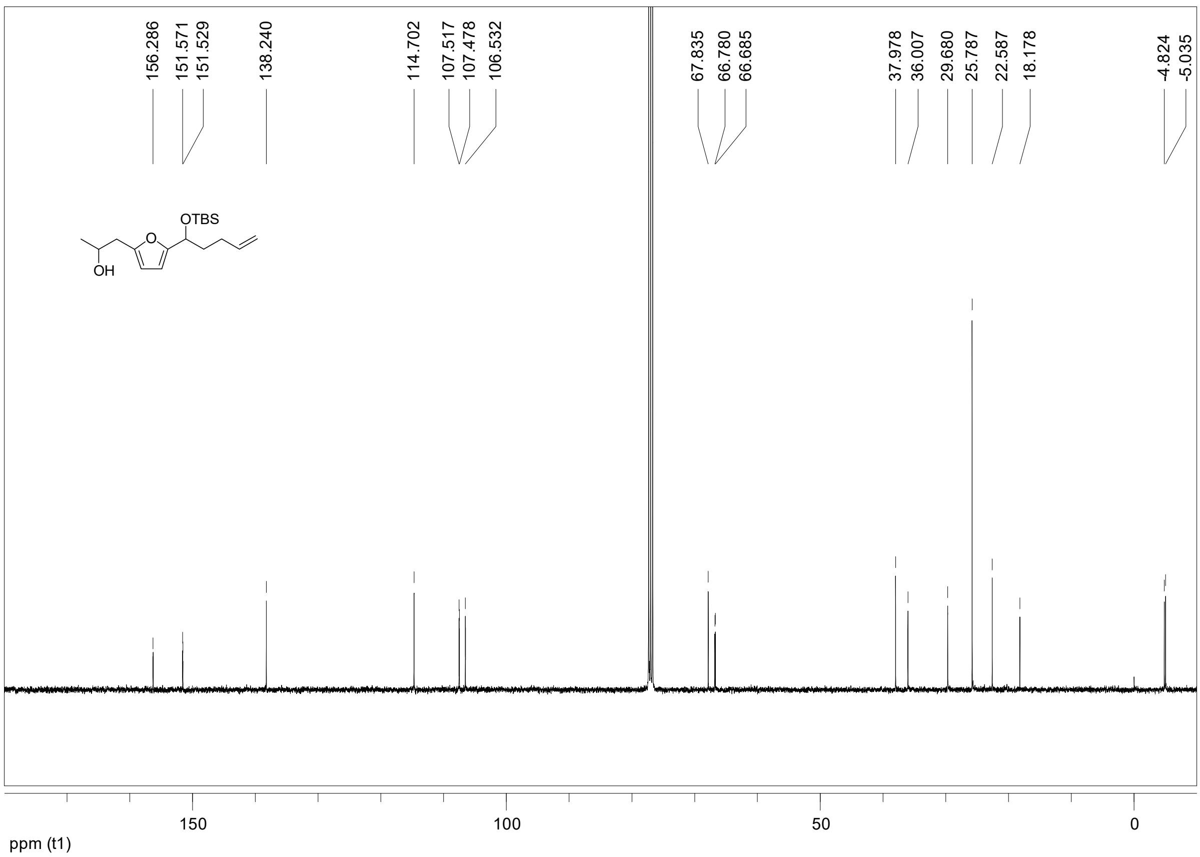

Figure S18: $^{13}C\{^{1}H\}$ NMR spectrum ($CDCl_3$, 100 MHz) of 1-[5-[1-(*tert*-Butyl-dimethyl-silanyloxy)-pent-4-enyl]-furan-2-yl]-propan-2-ol (3d)

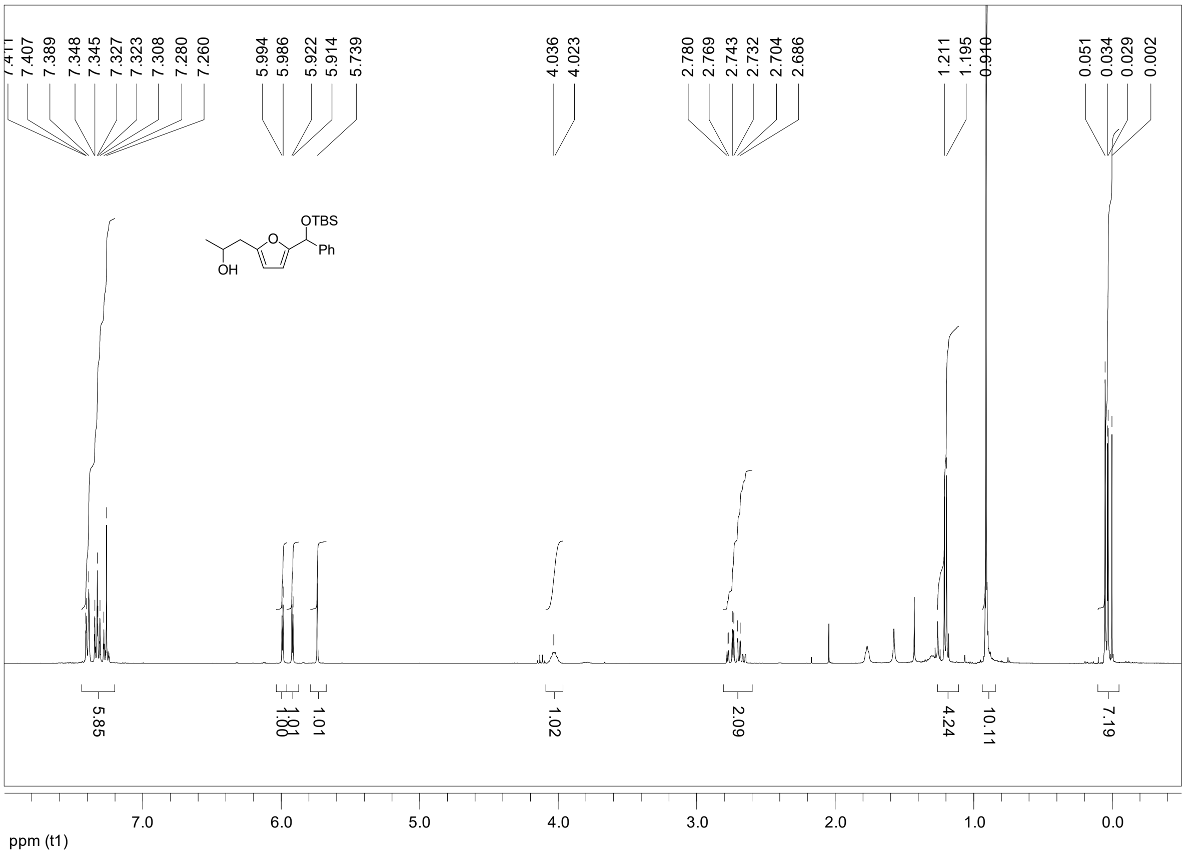

Figure S19: ^{1}H NMR spectrum ($CDCl_3$, 400 MHz) of 1-[5-[(*tert*-Butyl-dimethyl-silanyloxy)-phenyl-methyl]-furan-2-yl]-propan-2-ol (3e)

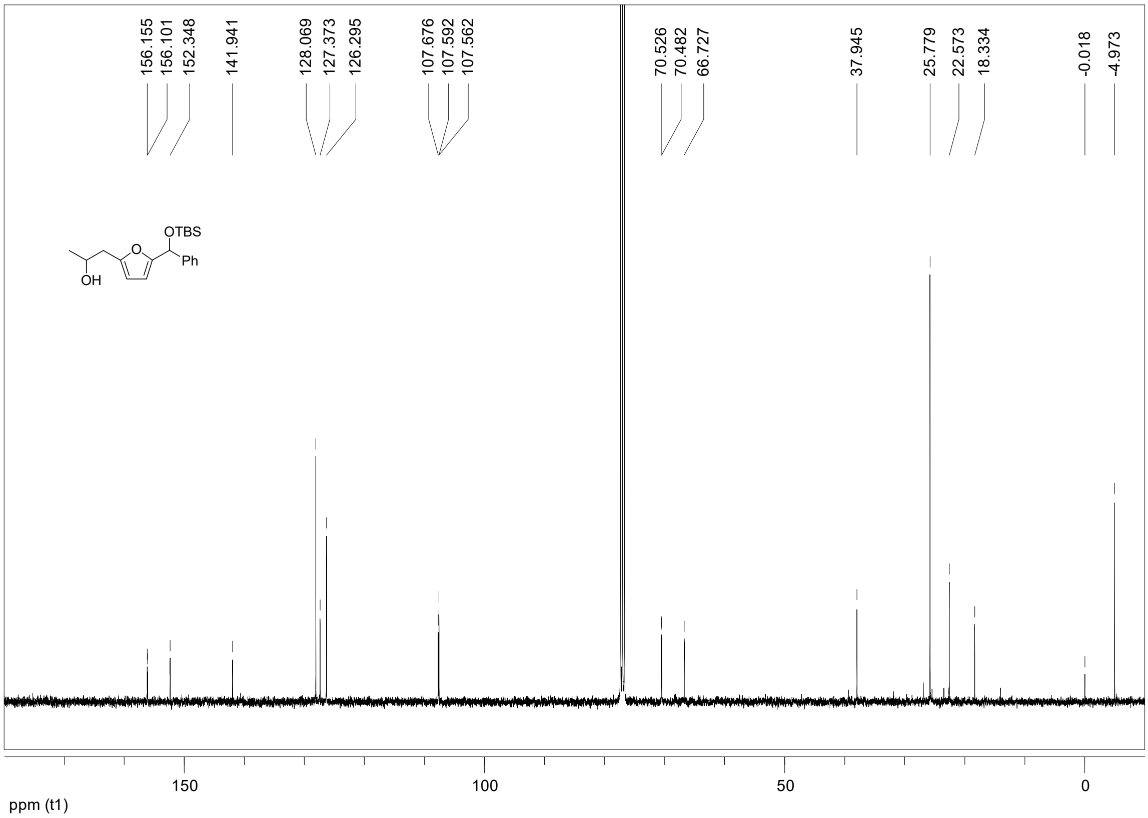

Figure S20: $^{13}C\{^{1}H\}$ NMR spectrum ($CDCl_3$, 100 MHz) of 1-[5-[(*tert*-Butyl-dimethyl-silanyloxy)-phenyl-methyl]-furan-2-yl]-propan-2-ol (3e)

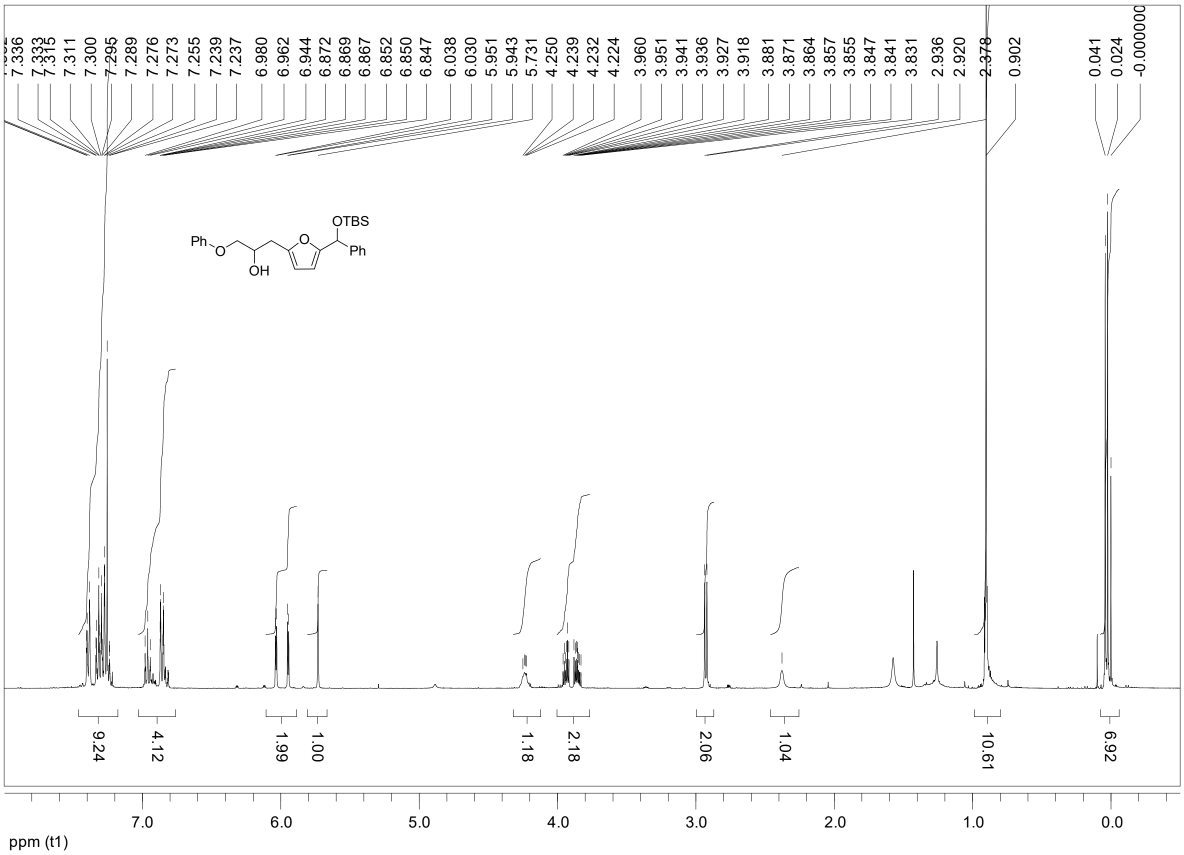

Figure S21: ^{1}H NMR spectrum ($CDCl_3$, 400 MHz) of 1-[5-[(*tert*-Butyl-dimethyl-silanyloxy)-phenyl-methyl]-furan-2-yl]-3-phenoxy-propan-2-ol (3f)

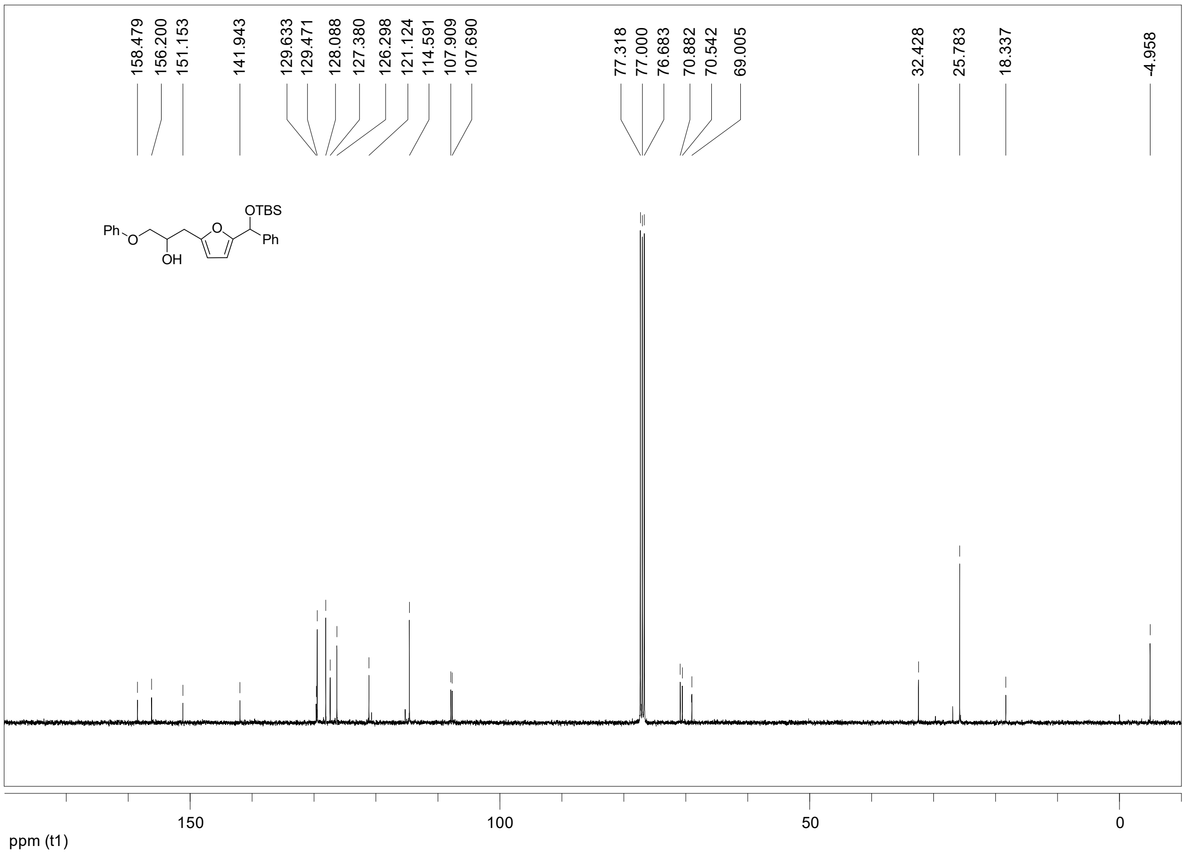

Figure S22: $^{13}C\{^1H\}$ NMR spectrum ($CDCl_3$, 100 MHz) of 1-[5-[(*tert*-Butyl-dimethyl-silanyloxy)-phenyl-methyl]-furan-2-yl]-3-phenoxy-propan-2-ol (3f)

3) NMR spectra of compounds **4a-f**

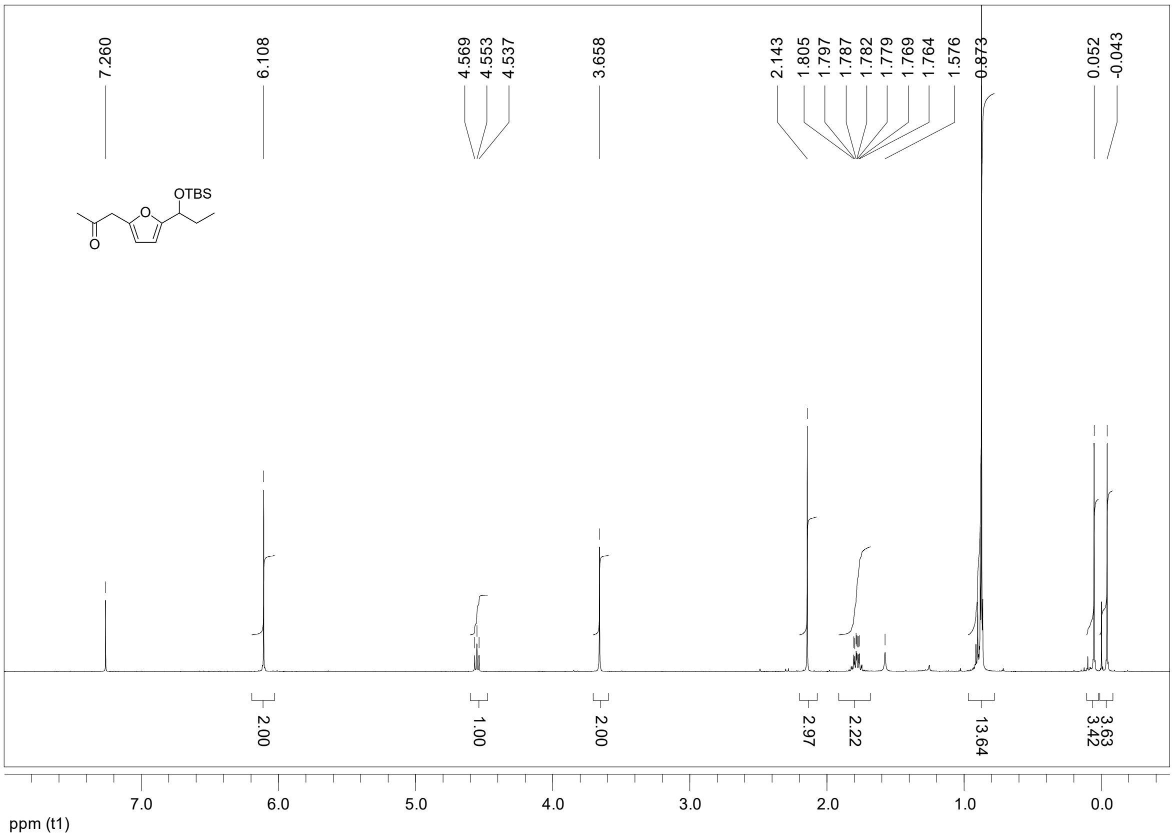

Figure S23: ^{1}H NMR spectrum ($CDCl_3$, 400 MHz) of 1-[5-[1-(*tert*-Butyl-dimethyl-silanyloxy)-propyl]-furan-2-yl]-propan-2-one (4a)

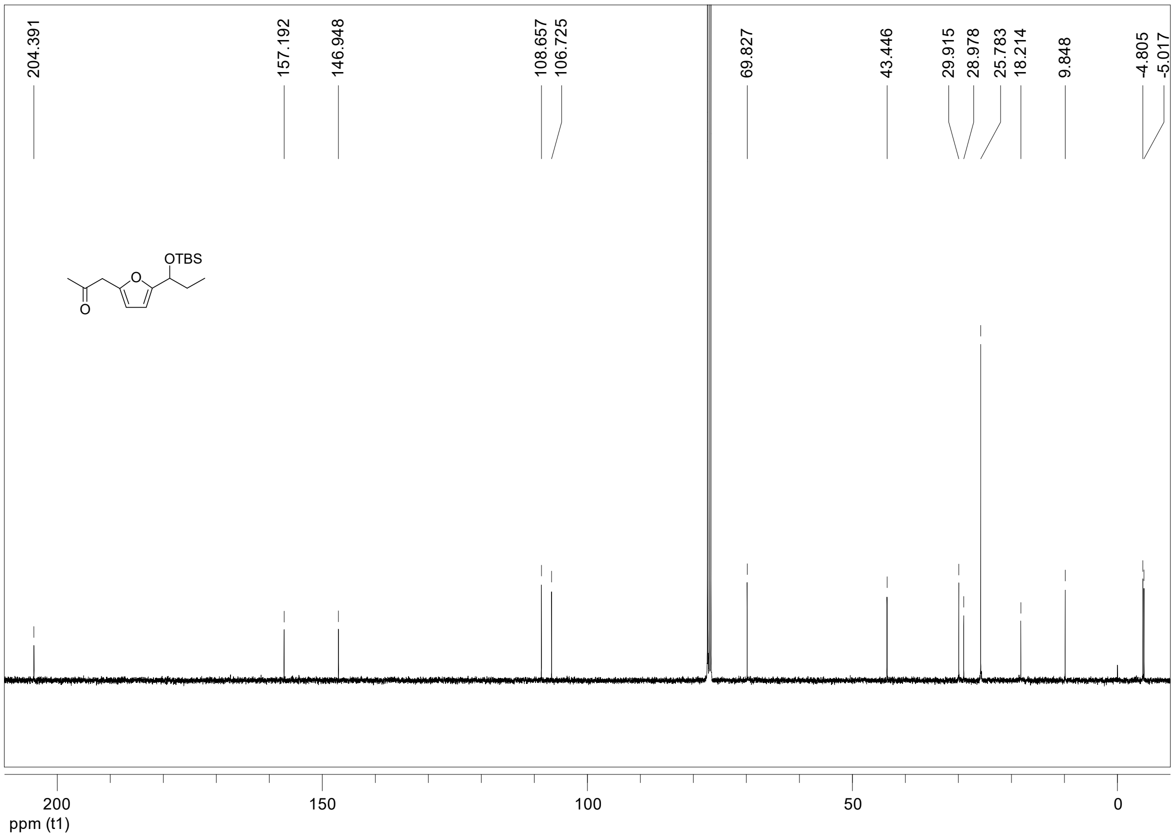

Figure S24: $^{13}C\{^{1}H\}$ NMR spectrum ($CDCl_3$, 100 MHz) of 1-[5-[1-(*tert*-Butyl-dimethyl-silanyloxy)-propyl]-furan-2-yl]-propan-2-one (4a)

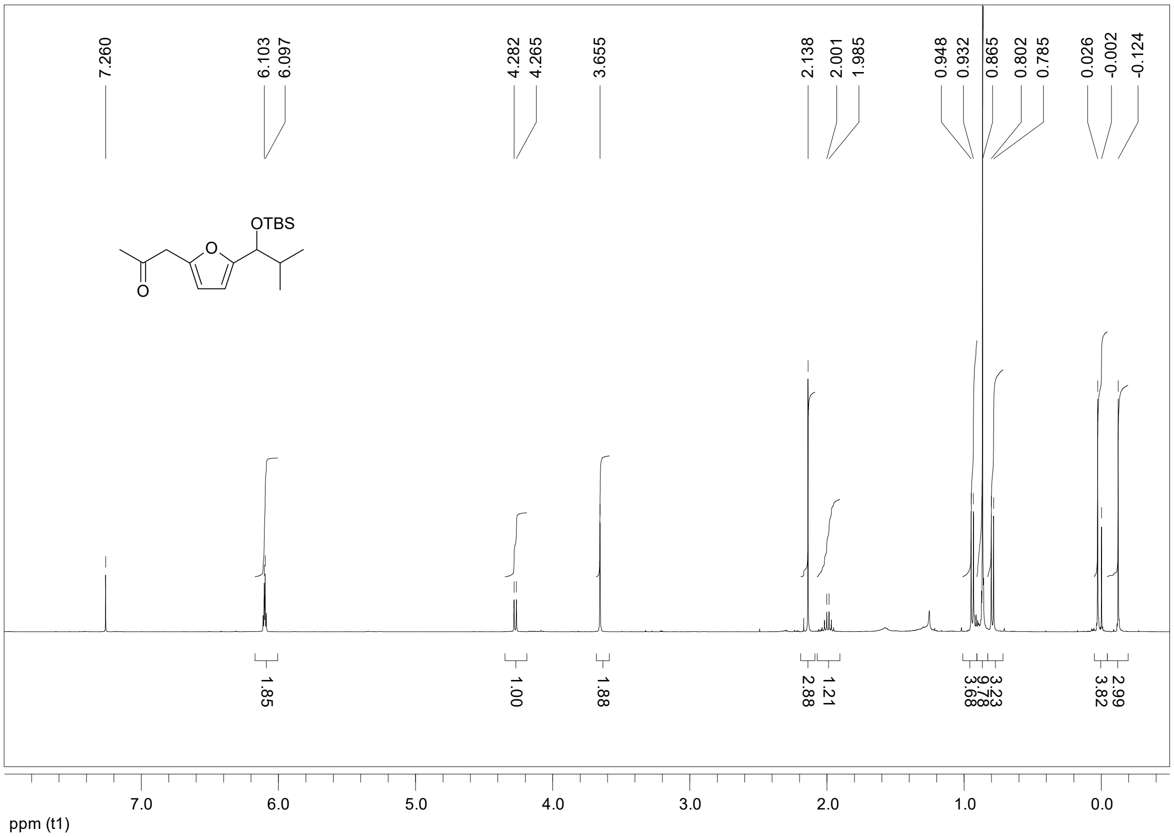

Figure S25: ^{1}H NMR spectrum ($CDCl_3$, 400 MHz) of 1-[5-[1-((*tert*-Butyl-dimethyl-silanyloxy)-2-methyl-propyl]-furan-2-yl]-propan-2-one (4b)

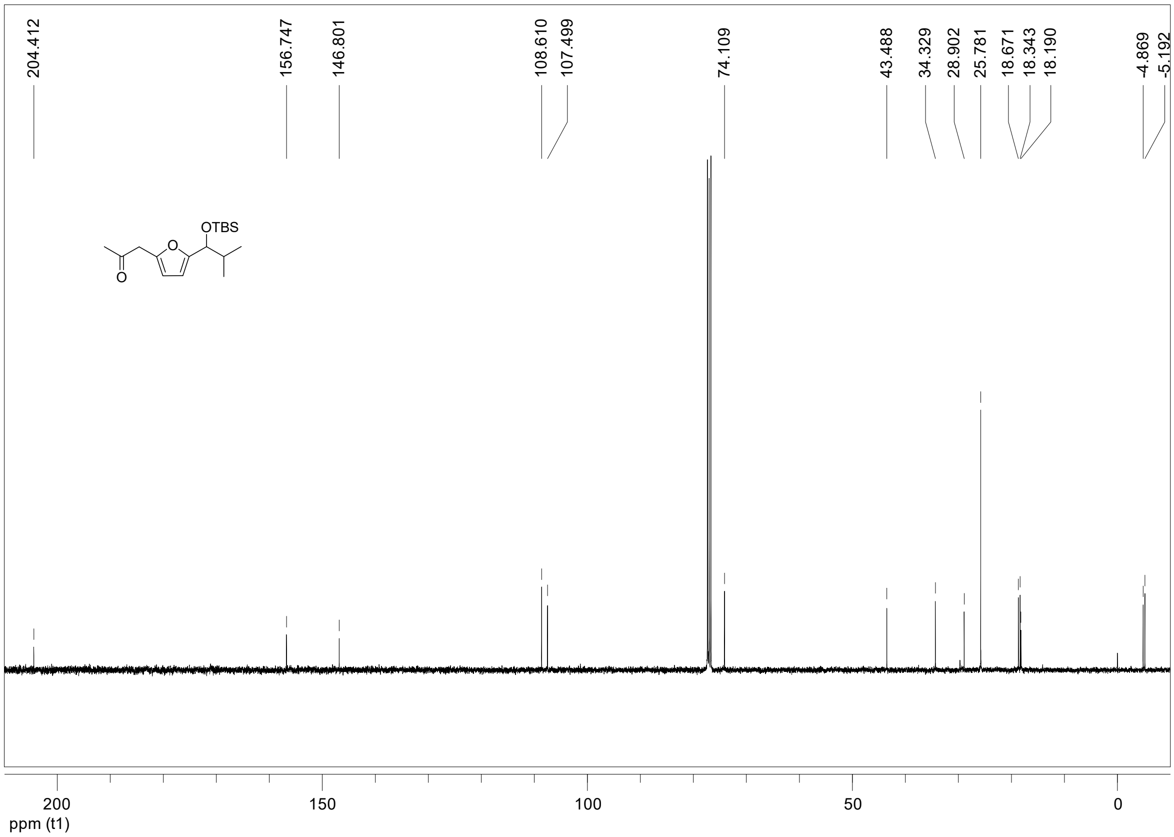

Figure S26: ^{13}C{^{1}H} NMR spectrum ($CDCl_3$, 100 MHz) of 1-[5-[1-((*tert*-Butyl-dimethyl-silanyloxy)-2-methyl-propyl]-furan-2-yl]-propan-2-one (4b)

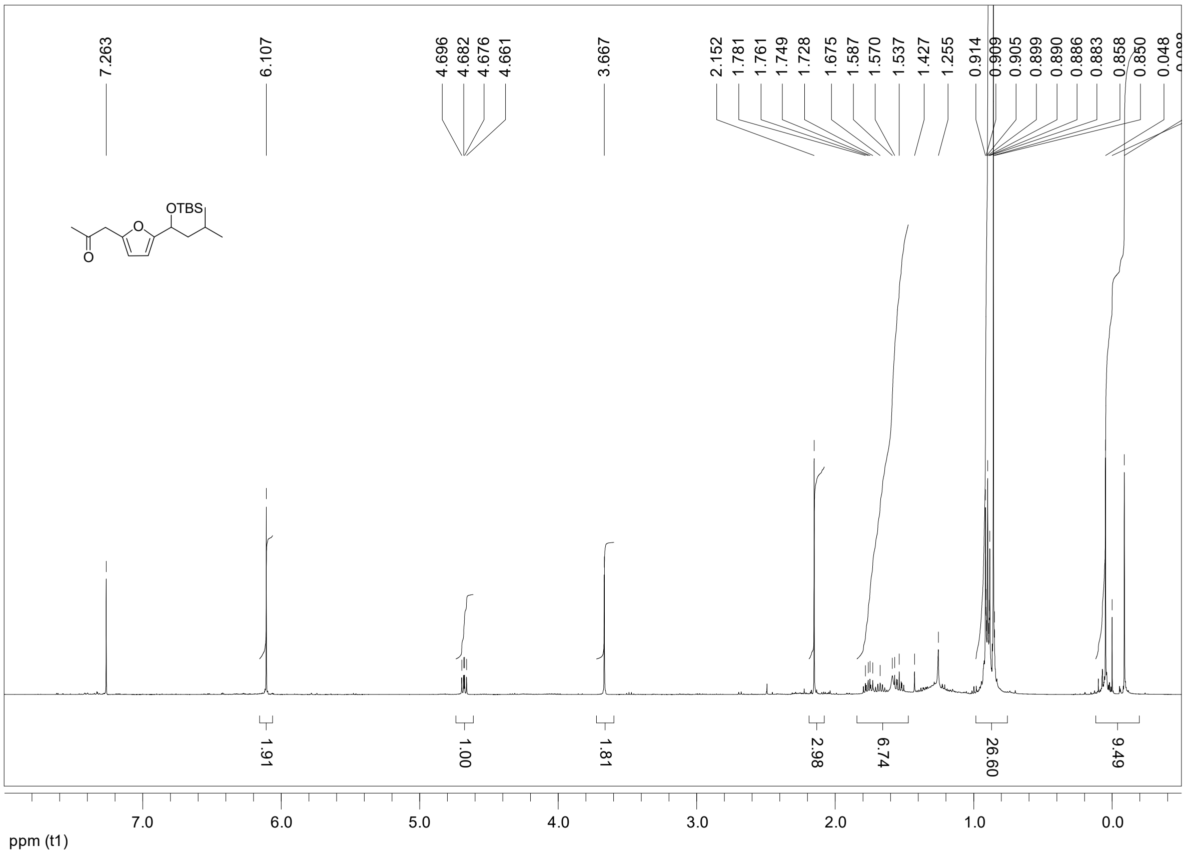

Figure S27: ^{1}H NMR spectrum ($CDCl_3$, 400 MHz) of 1-[5-[1-(*tert*-Butyl-dimethyl-silanyloxy)-3-methyl-butyl]-furan-2-yl]-propan-2-one (4c)

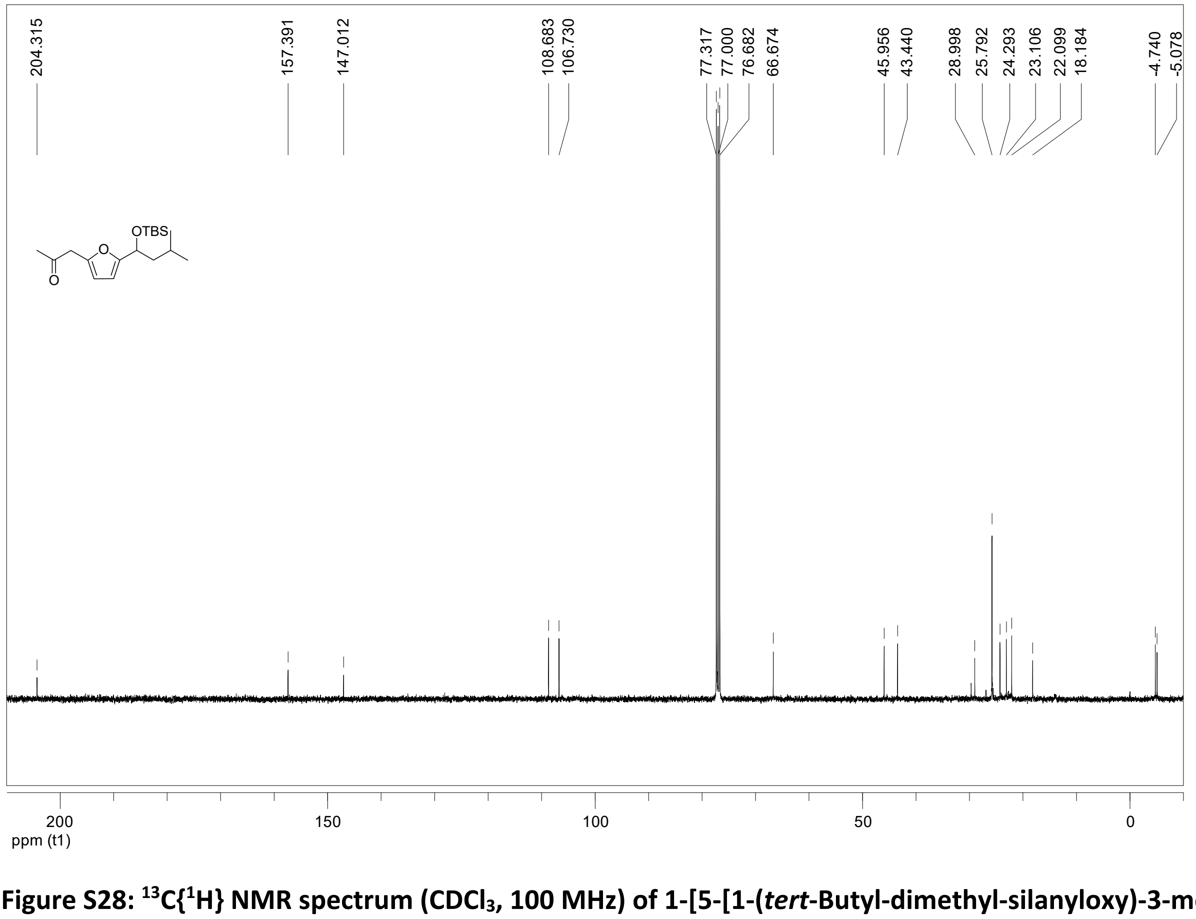

Figure S28: $^{13}C\{^{1}H\}$ NMR spectrum ($CDCl_3$, 100 MHz) of 1-[5-[1-(*tert*-Butyl-dimethyl-silanyloxy)-3-methyl-butyl]-furan-2-yl]-propan-2-one (4c)

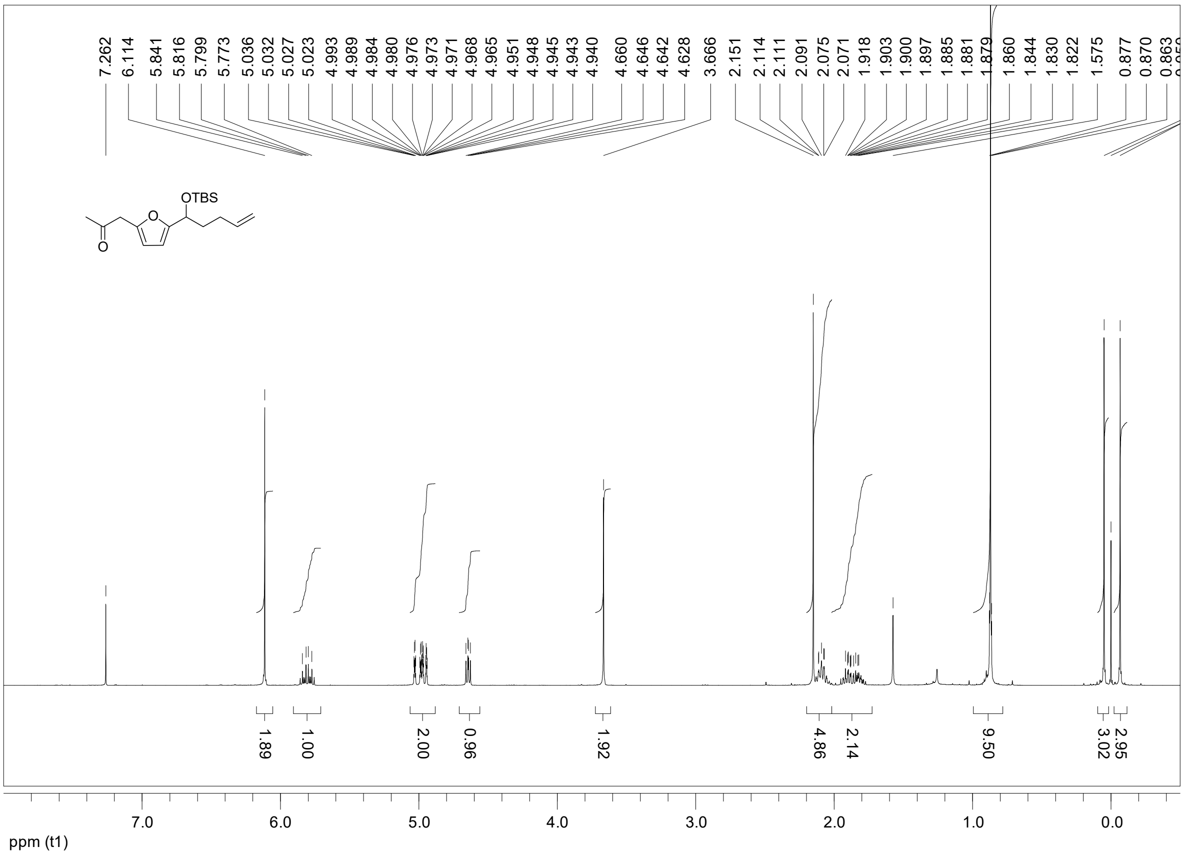

Figure S29: ^{1}H NMR spectrum ($CDCl_3$, 400 MHz) of 1-[5-[1-(*tert*-Butyl-dimethyl-silanyloxy)-pent-4-enyl]-furan-2-yl]-propan-2-one (4d)

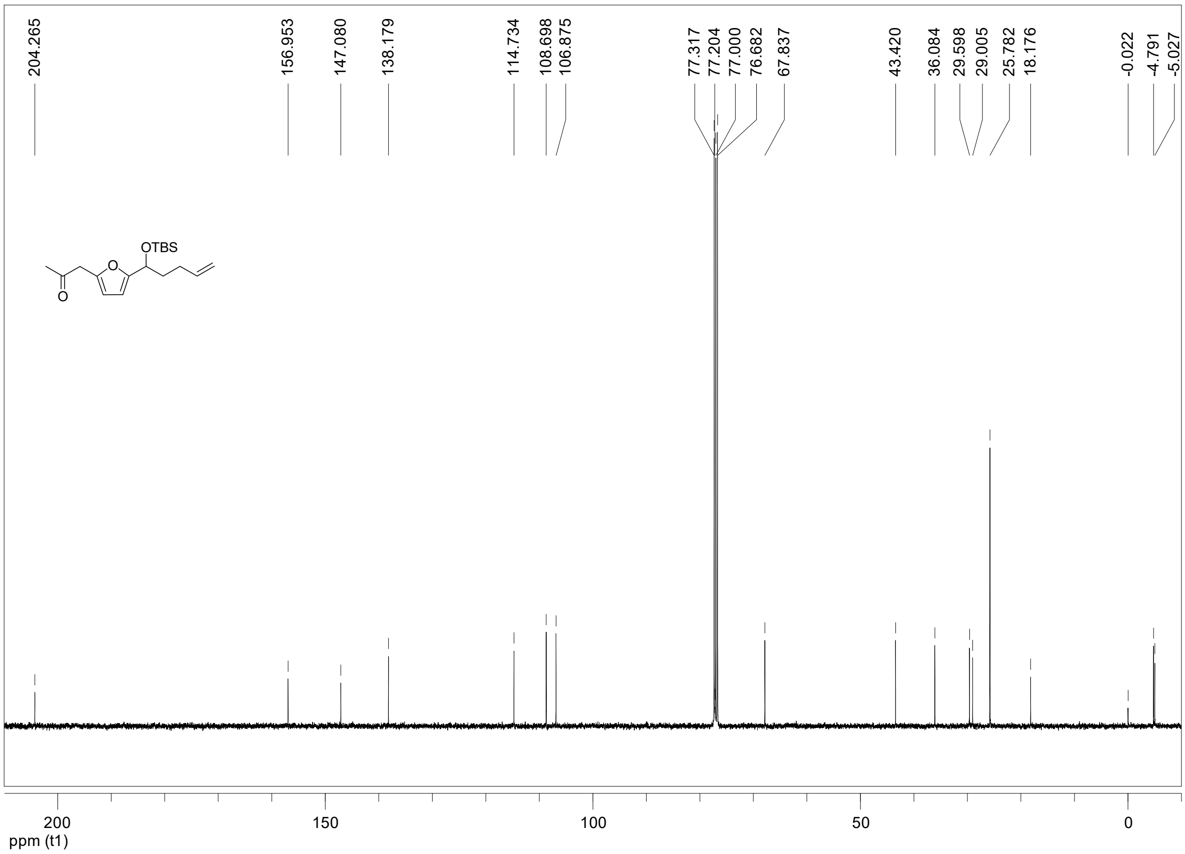

Figure S30: $^{13}C\{^1H\}$ NMR spectrum ($CDCl_3$, 100 MHz) of 1-[5-[1-(*tert*-Butyl-dimethyl-silanyloxy)-pent-4-enyl]-furan-2-yl]-propan-2-one (4d)

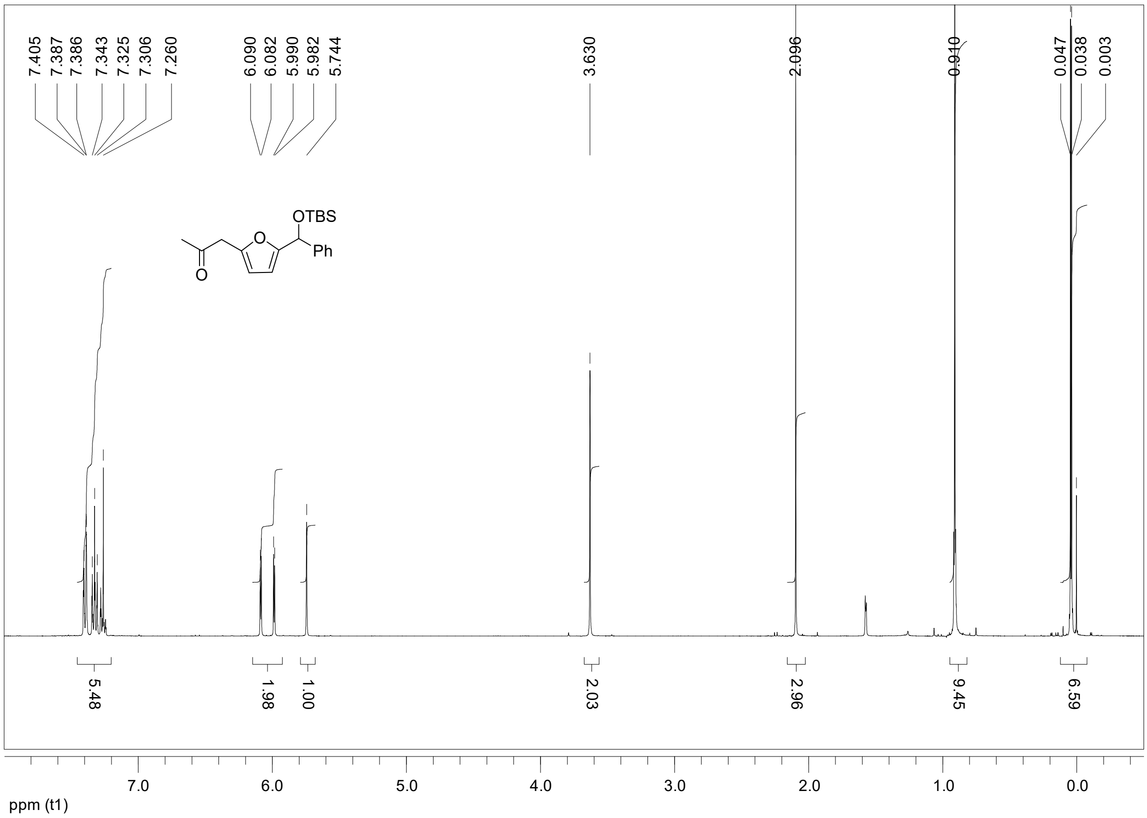

Figure S31: ^{1}H NMR spectrum ($CDCl_3$, 400 MHz) of 1-[5-[(*tert*-Butyl-dimethyl-silanyloxy)-phenyl-methyl]-furan-2-yl]-propan-2-one (4e)

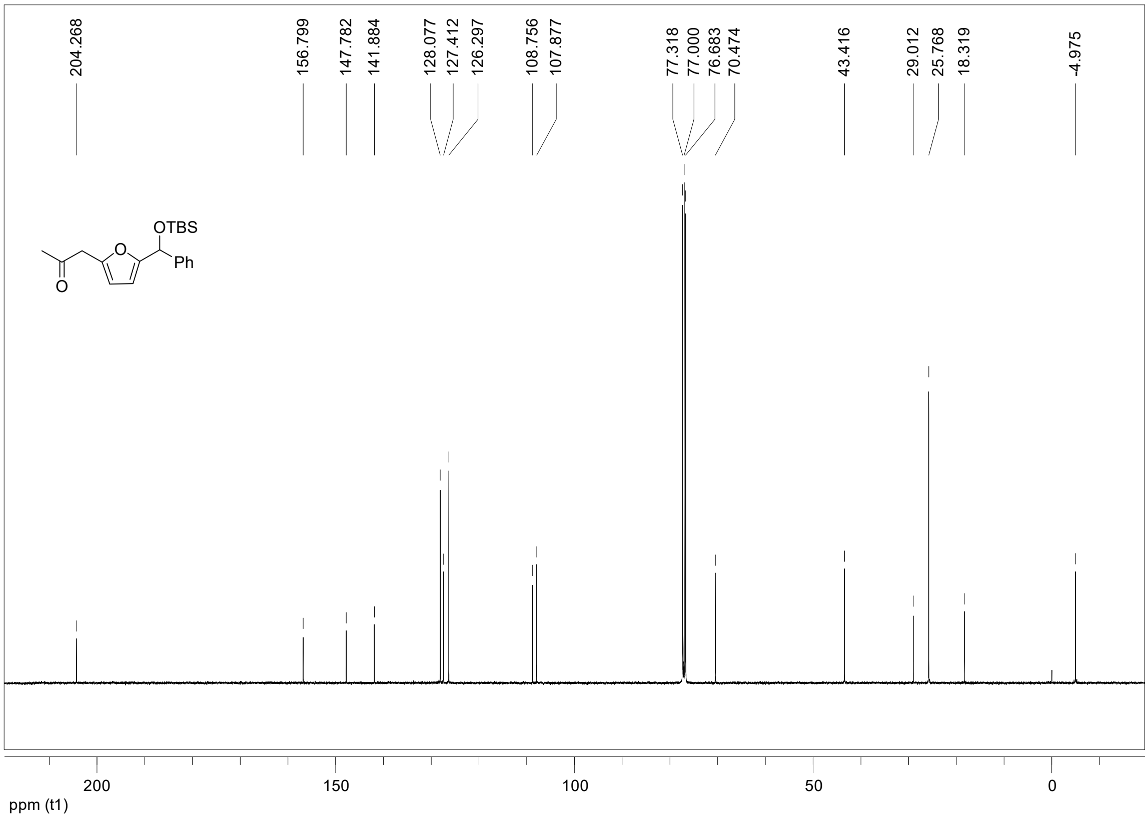

Figure S32: $^{13}C\{^{1}H\}$ NMR spectrum ($CDCl_3$, 100 MHz) of 1-[5-[(*tert*-Butyl-dimethyl-silanyloxy)-phenyl-methyl]-furan-2-yl]-propan-2-one (4e)

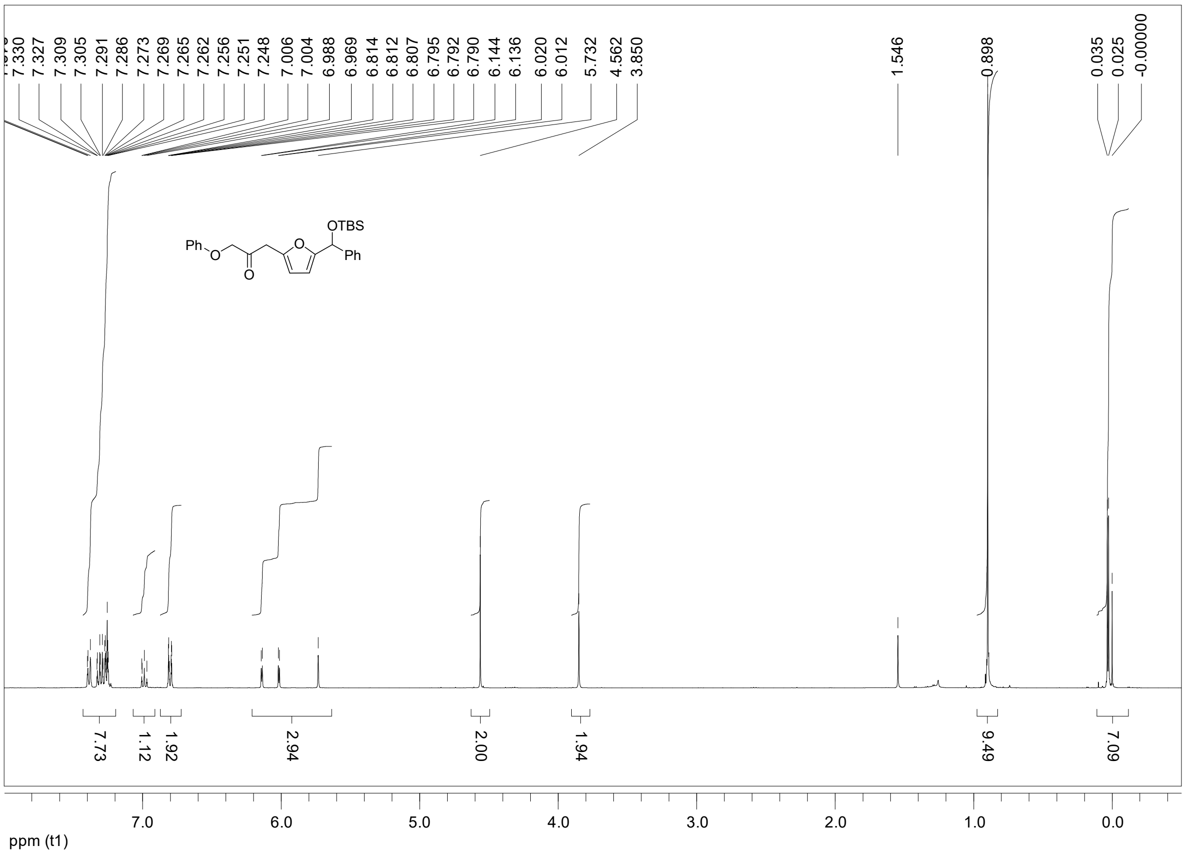

Figure S33: ^{1}H NMR spectrum ($CDCl_3$, 400 MHz) of 1-[5-[(*tert*-Butyl-dimethyl-silanyloxy)-phenyl-methyl]-furan-2-yl]-3-phenoxy-propan-2-one (4f)

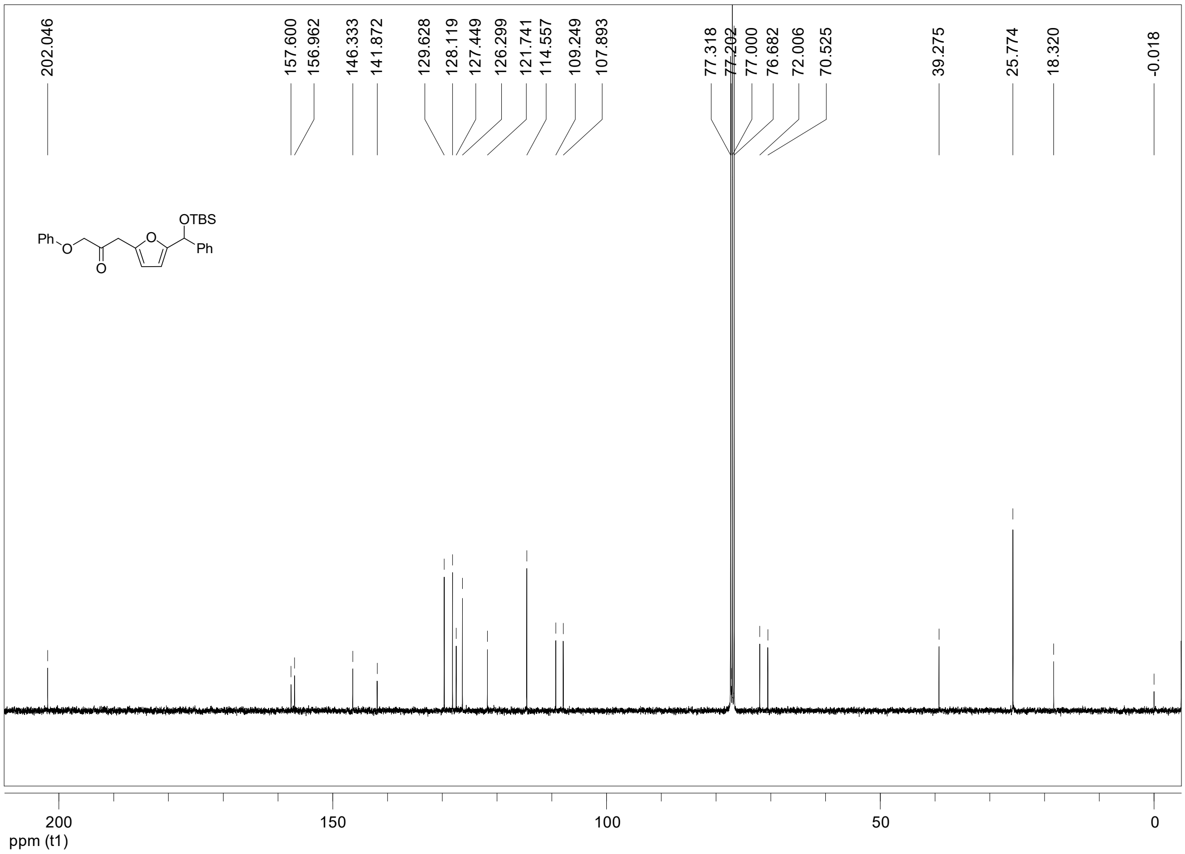

Figure S34: $^{13}C\{^{1}H\}$ NMR spectrum ($CDCl_3$, 100 MHz) of 1-[5-[(*tert*-Butyl-dimethyl-silanyloxy)-phenyl-methyl]-furan-2-yl]-3-phenoxy-propan-2-one (4f)

4) NMR spectra of keto alcohols **5a-f**

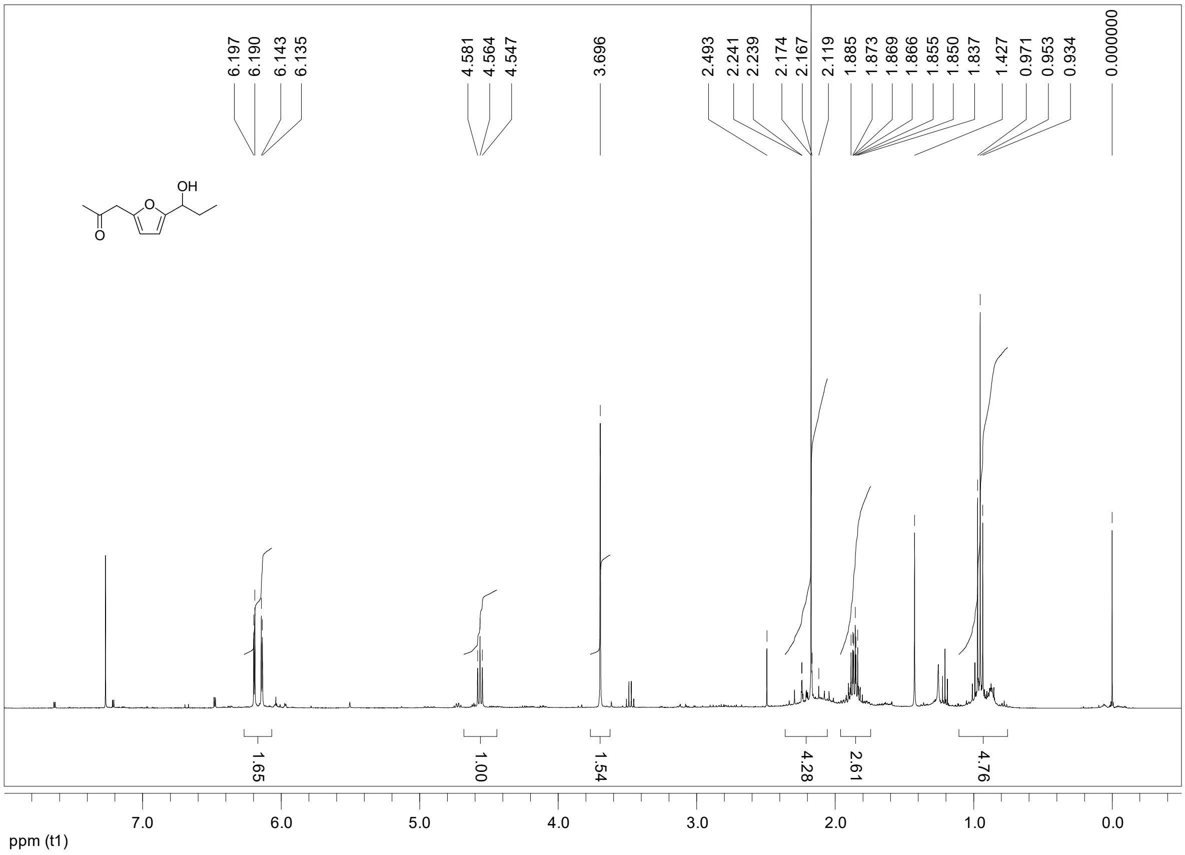

Figure S35: ^{1}H NMR spectrum ($CDCl_3$, 400 MHz) of 1-[5-(1-Hydroxy-propyl)-furan-2-yl]-propan-2-one (5a)

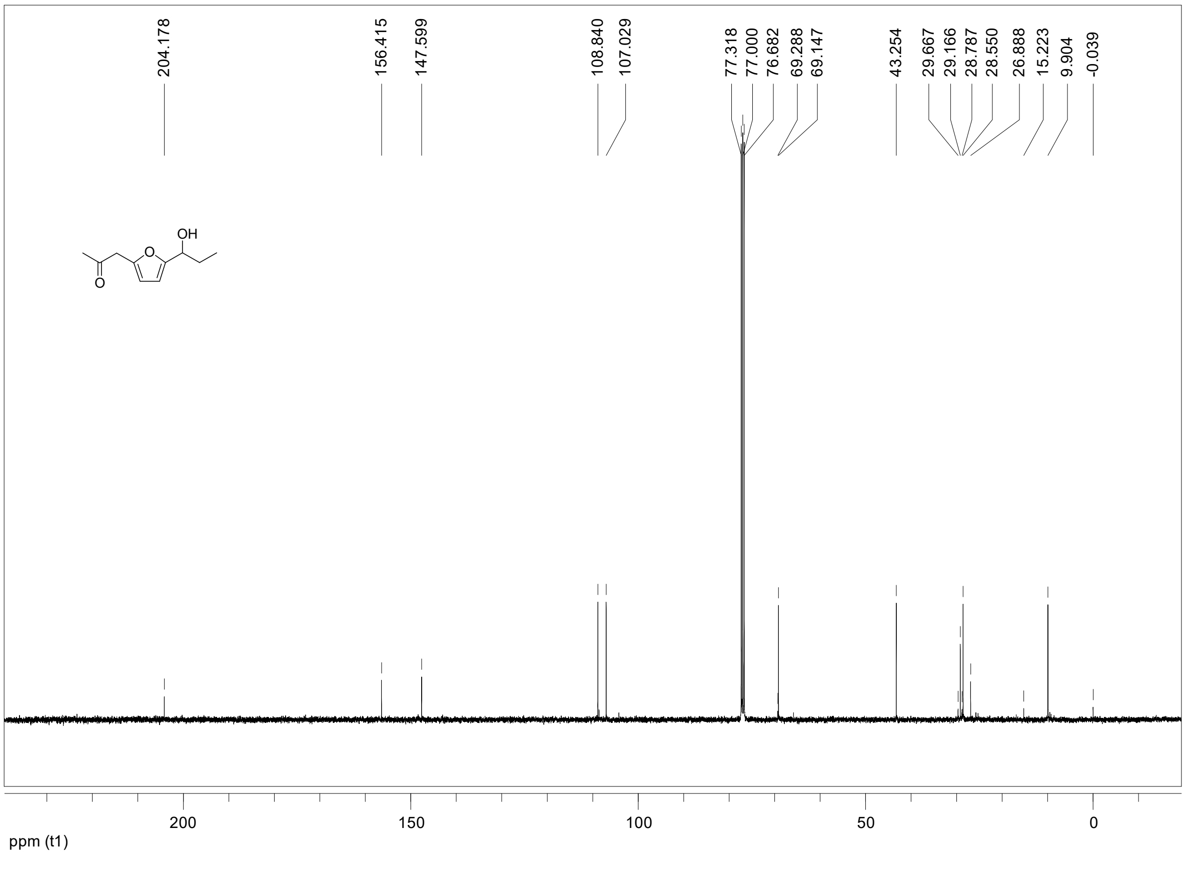

Figure S36: $^{13}C\{^1H\}$ NMR spectrum ($CDCl_3$, 100 MHz) of 1-[5-(1-Hydroxy-propyl)-furan-2-yl]-propan-2-one (5a)

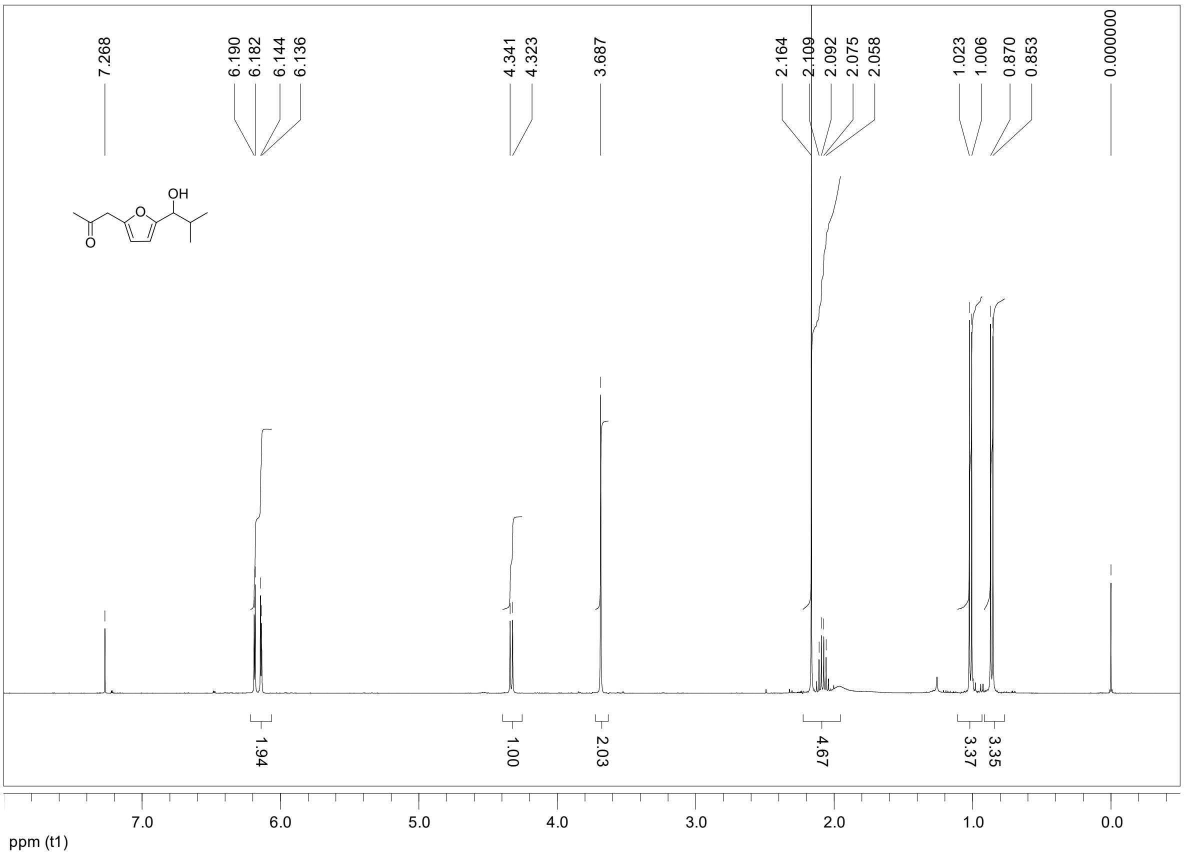

Figure S37: ^{1}H NMR spectrum ($CDCl_3$, 400 MHz) of 1-[5-(1-Hydroxy-2-methyl-propyl)-furan-2-yl]-propan-2-one (5b)

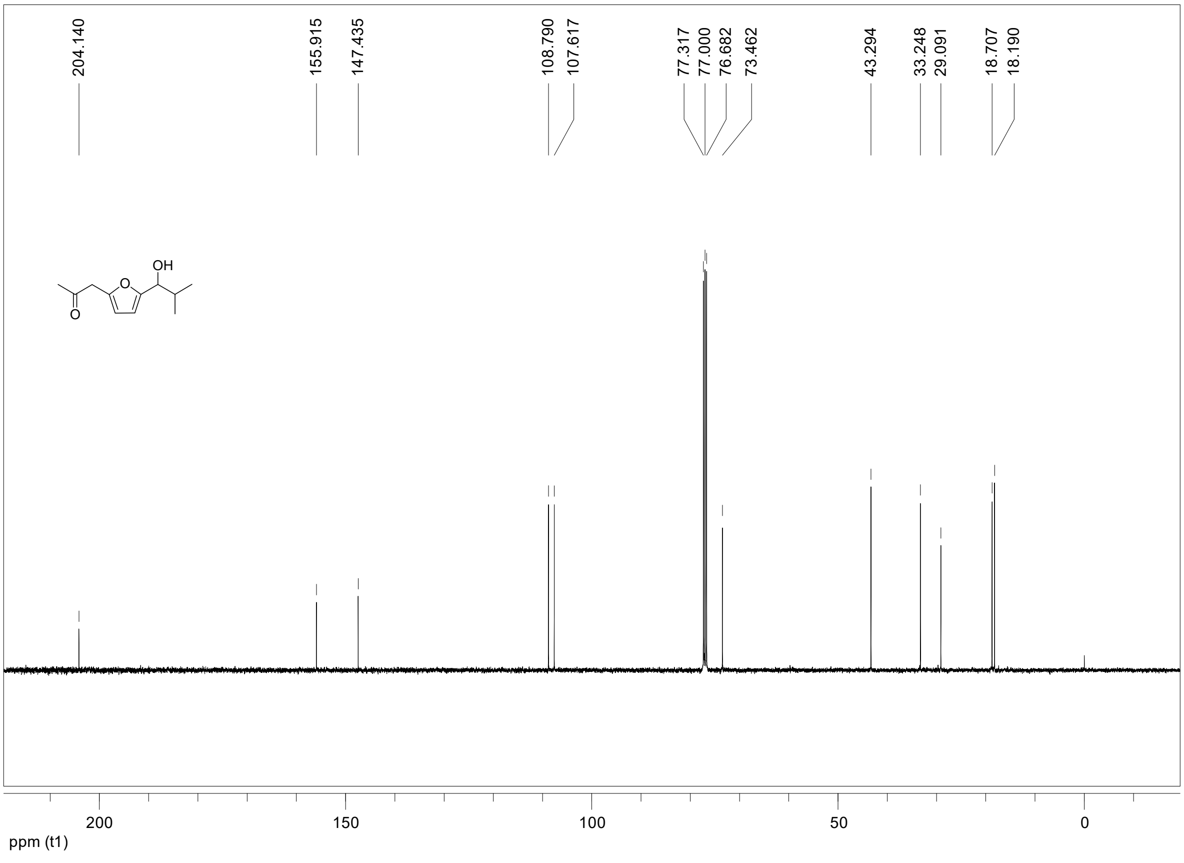

Figure S38: ^{13}C{^{1}H} NMR spectrum ($CDCl_3$, 100 MHz) of 1-[5-(1-Hydroxy-2-methyl-propyl)-furan-2-yl]-propan-2-one (5b)

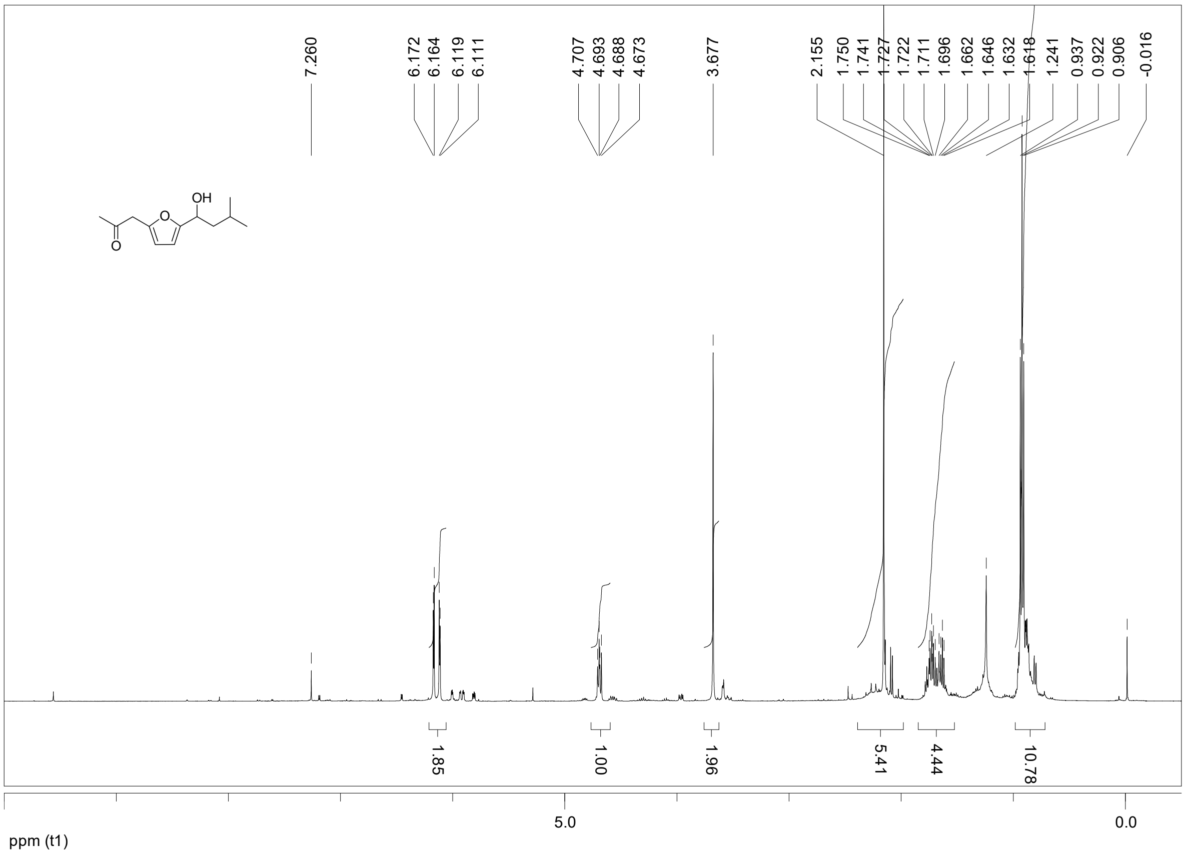

Figure S39: ^{1}H NMR spectrum ($CDCl_3$, 400 MHz) of 1-[5-(1-Hydroxy-3-methyl-butyl)-furan-2-yl]-propan-2-one (5c)

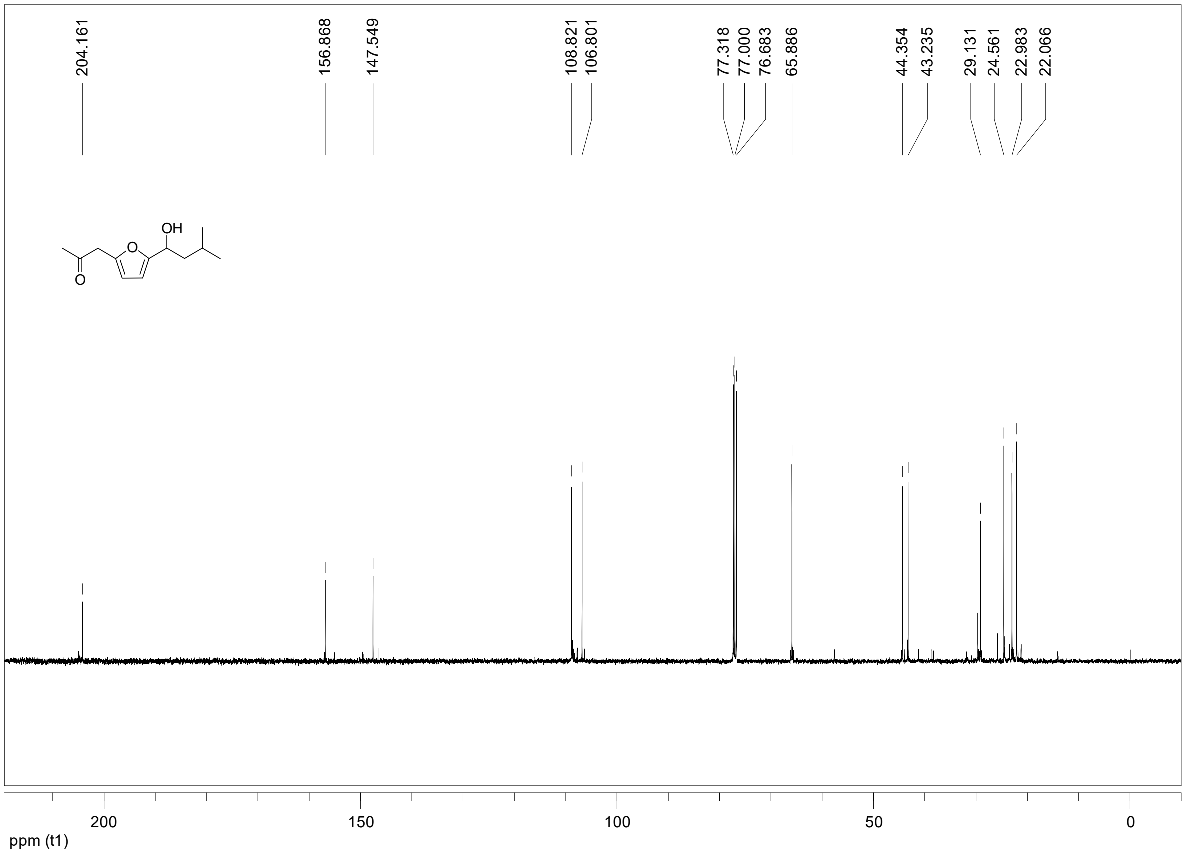

Figure S40: $^{13}C\{^{1}H\}$ NMR spectrum ($CDCl_3$, 100 MHz) of 1-[5-(1-Hydroxy-3-methyl-butyl)-furan-2-yl]-propan-2-one (5c)

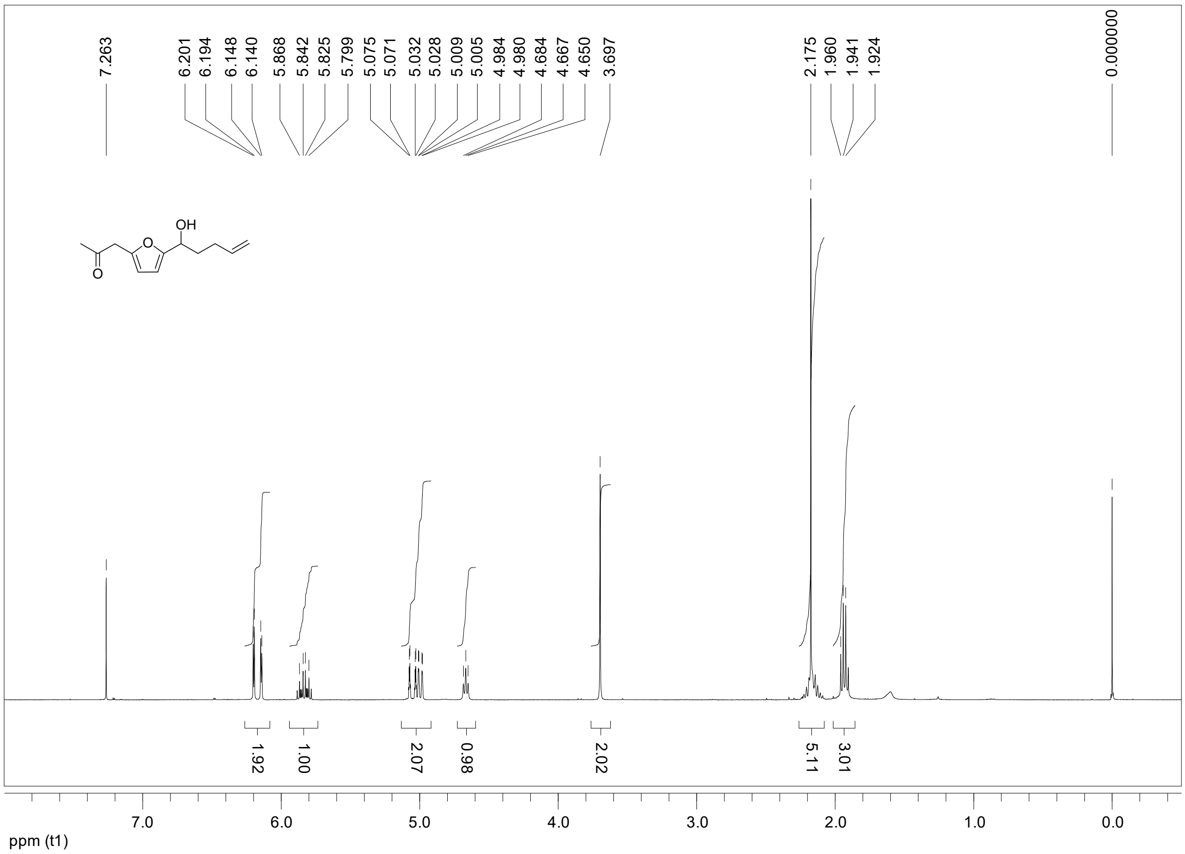

Figure S41: ^{1}H NMR spectrum ($CDCl_3$, 400 MHz) of 1-[5-(1-Hydroxy-pent-4-enyl)-furan-2-yl]-propan-2-one (5d)

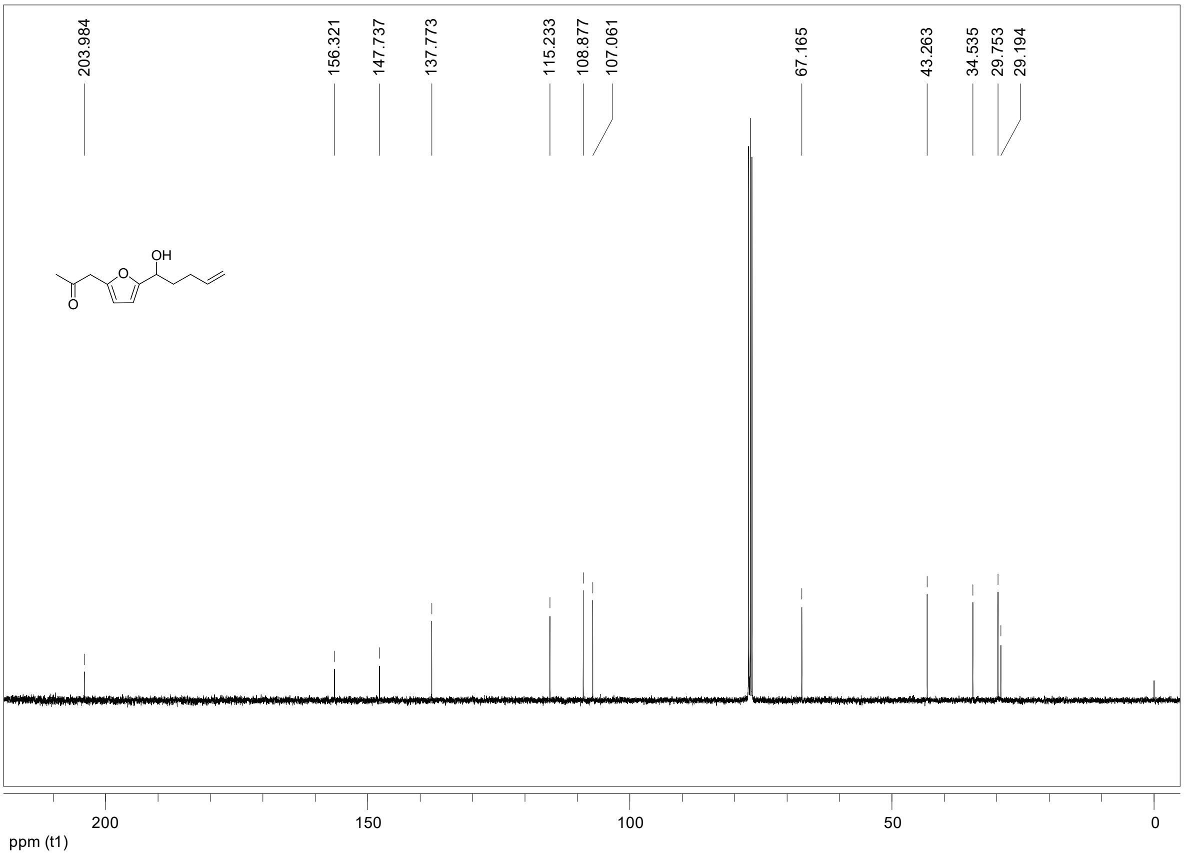

Figure S42: $^{13}C\{^{1}H\}$ NMR spectrum ($CDCl_3$, 100 MHz) of 1-[5-(1-Hydroxy-pent-4-enyl)-furan-2-yl]-propan-2-one (5d)

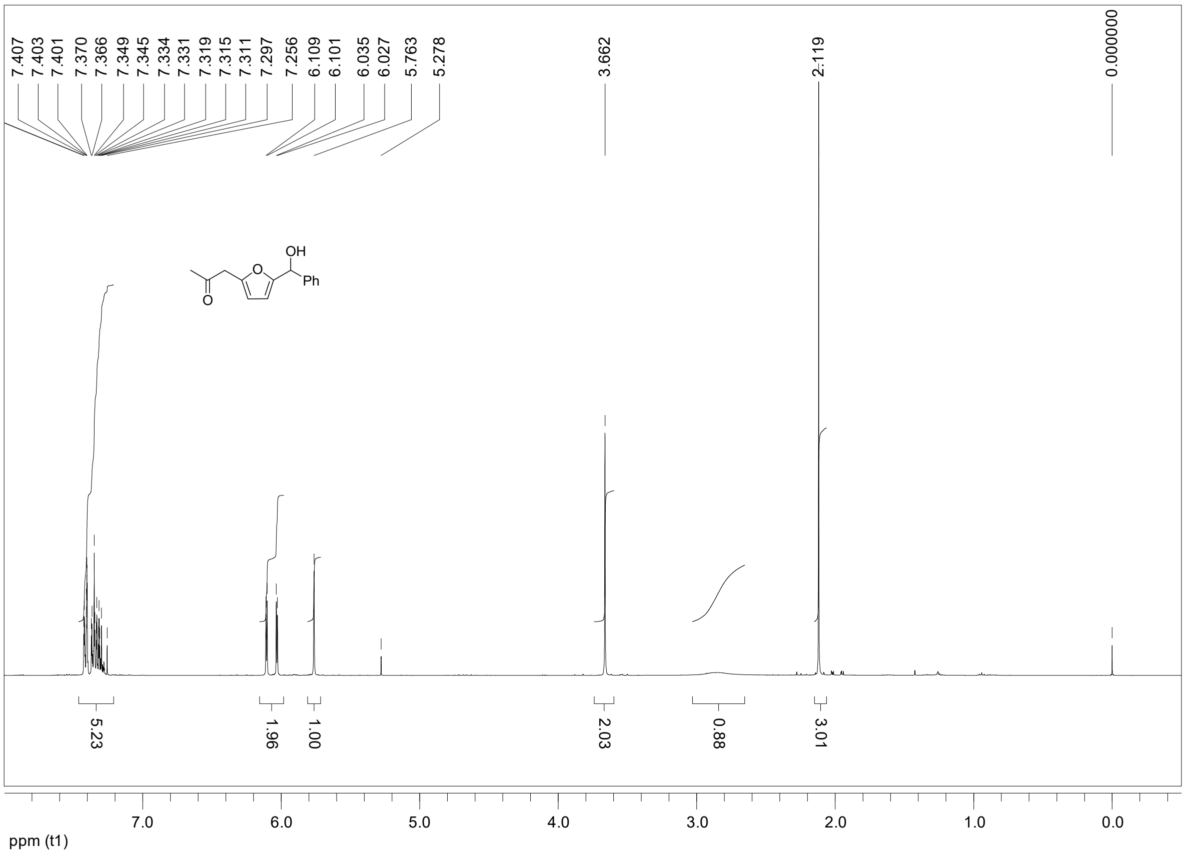

Figure S43: ^{1}H NMR spectrum ($CDCl_3$, 400 MHz) of 1-[5-(Hydroxy-phenyl-methyl)-furan-2-yl]-propan-2-one (5e)

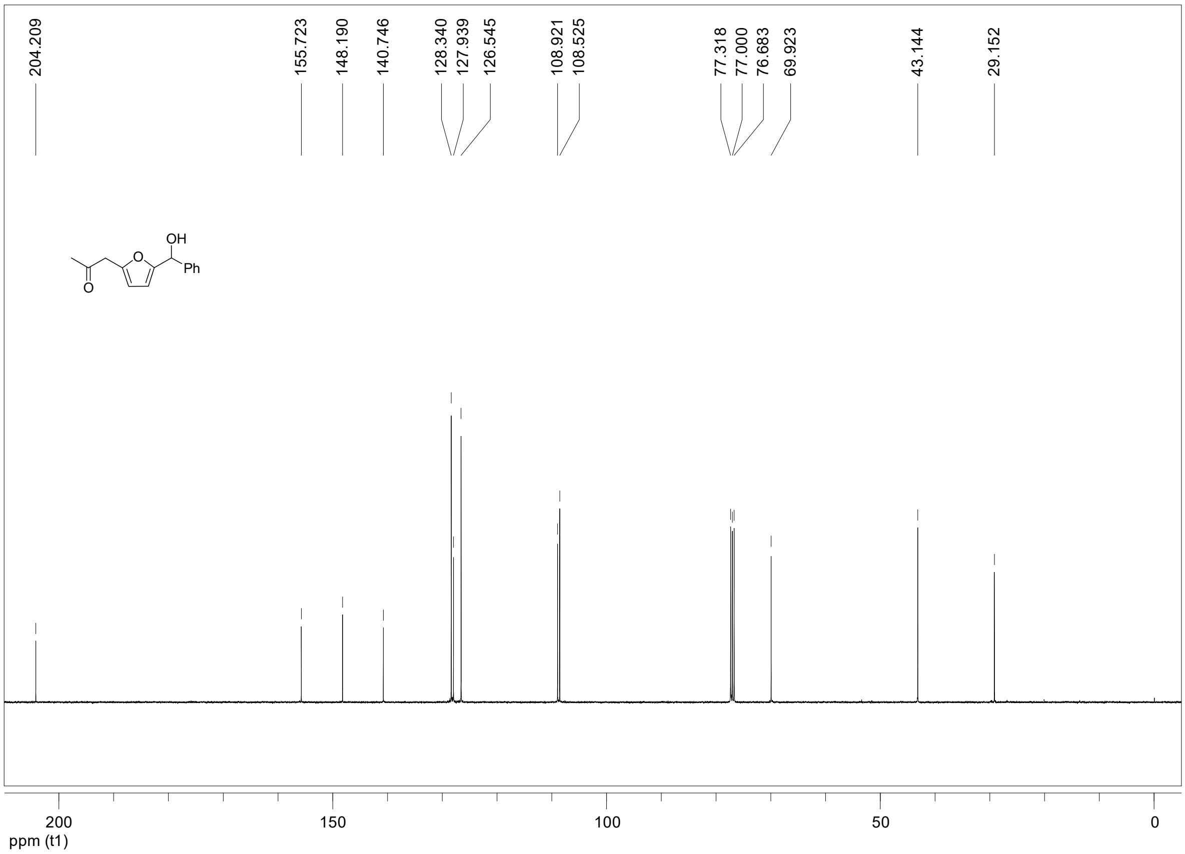

Figure S44: $^{13}C\{^1H\}$ NMR spectrum ($CDCl_3$, 100 MHz) of 1-[5-(Hydroxy-phenyl-methyl)-furan-2-yl]-propan-2-one (5e)

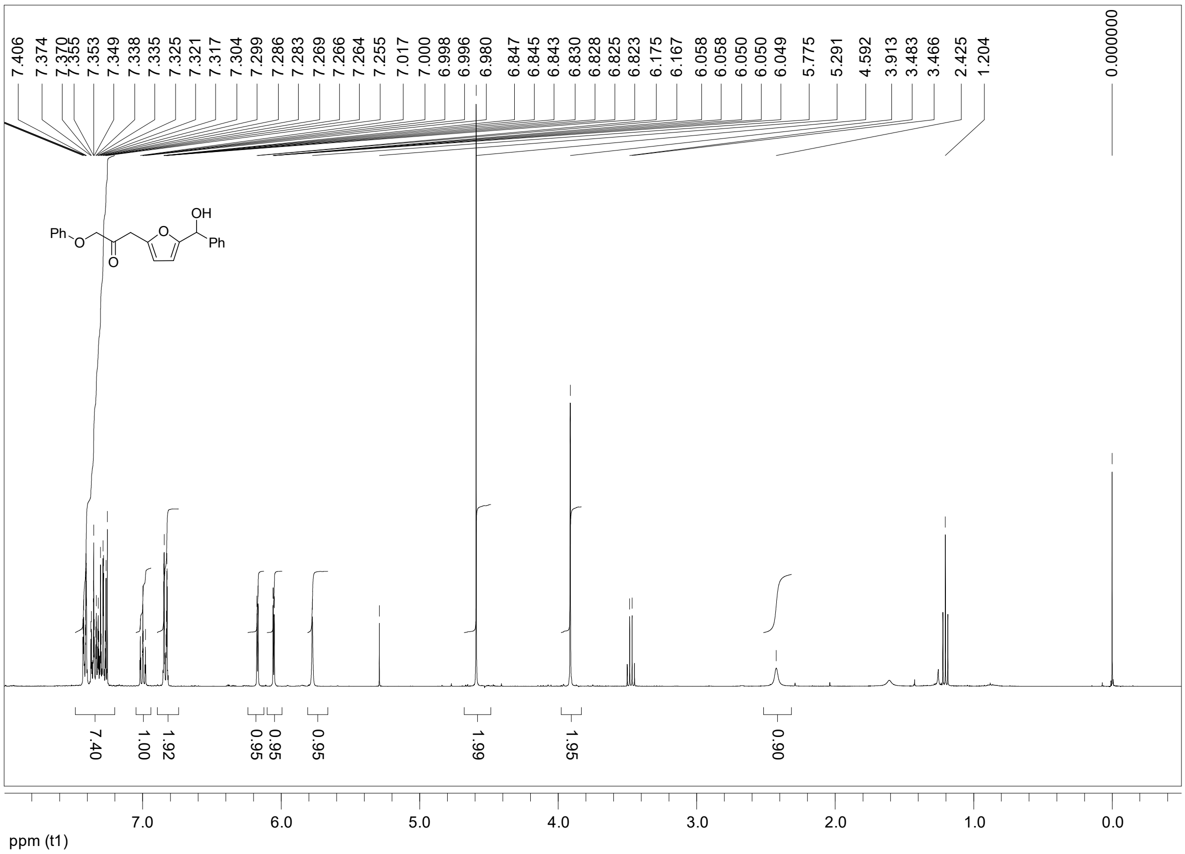

Figure S45: ^{1}H NMR spectrum (CDCl$_3$, 400 MHz) of 1-[5-(Hydroxy-phenyl-methyl)-furan-2-yl]-3-phenoxy-propan-2-one (5f)

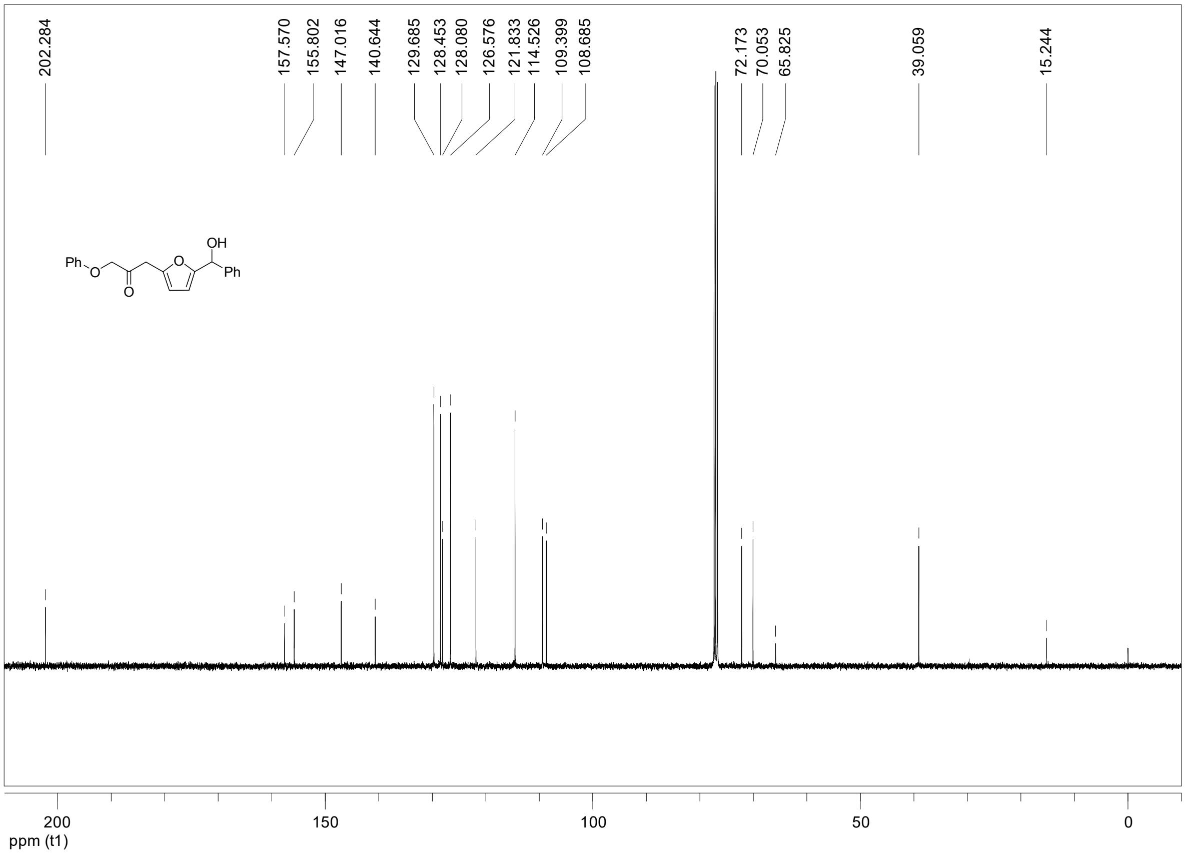

Figure S46: ^{13}C{^{1}H} NMR spectrum (CDCl$_3$, 100 MHz) of 1-[5-(Hydroxy-phenyl-methyl)-furan-2-yl]-3-phenoxy-propan-2-one (5f)

5) NMR spectra of compounds **7a-d**

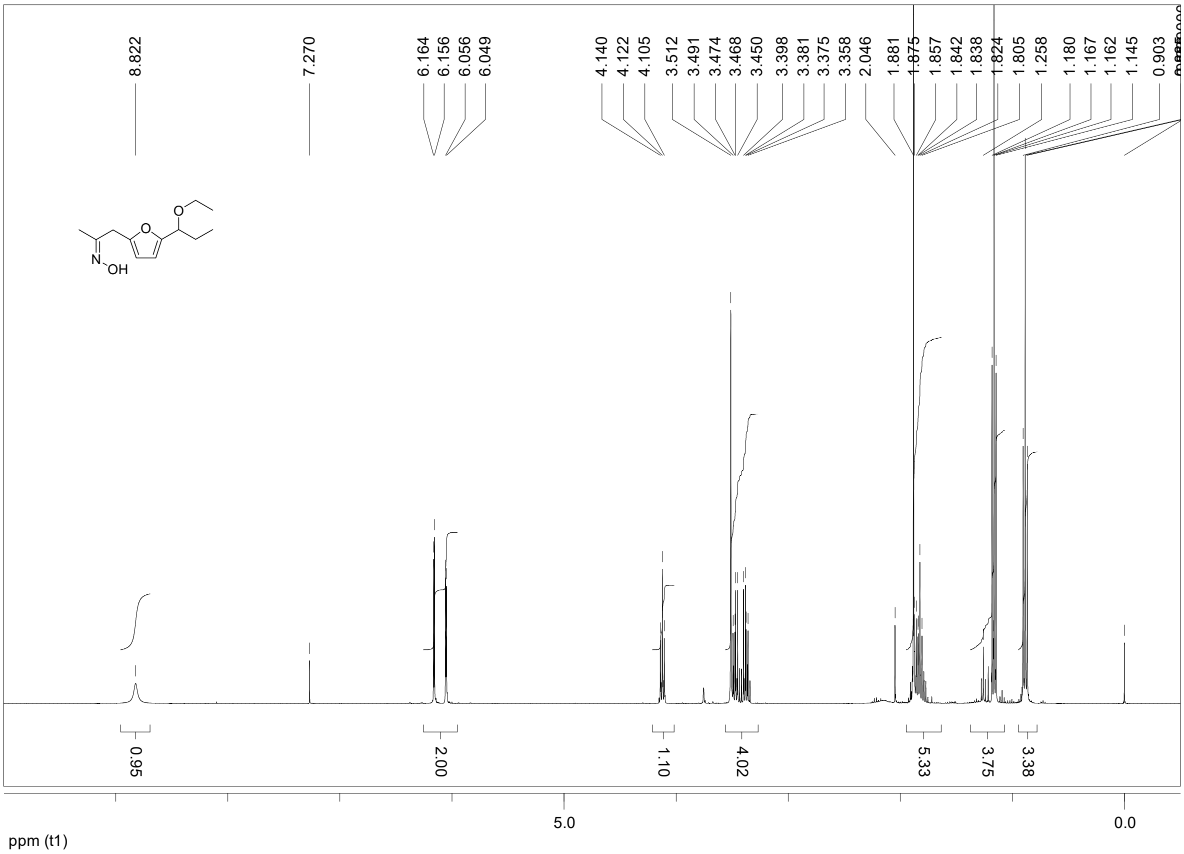

Figure S47: ^{1}H NMR spectrum ($CDCl_3$, 400 MHz) of 1-[5-(1-Ethoxy-propyl)-furan-2-yl]-propan-2-one oxime (7a)

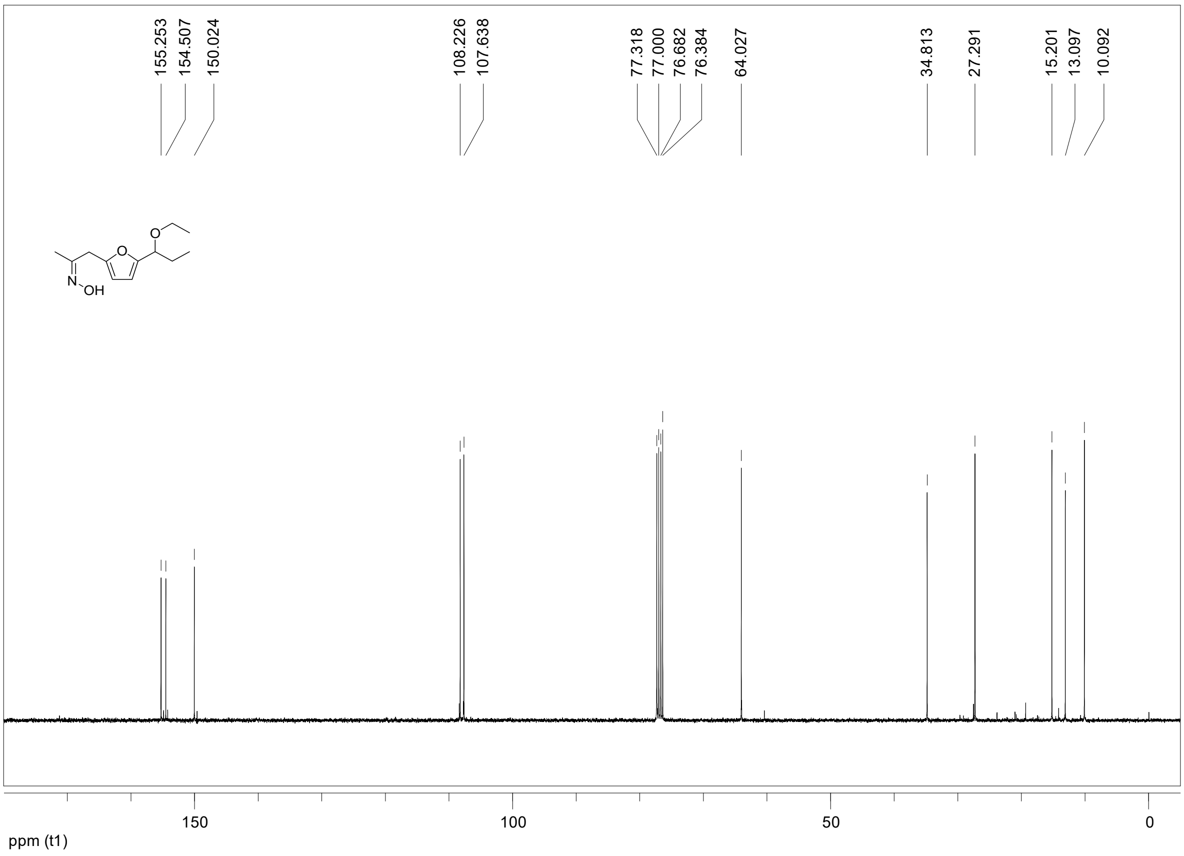

Figure S48: $^{13}C\{^{1}H\}$ NMR spectrum ($CDCl_3$, 100 MHz) of 1-[5-(1-Ethoxy-propyl)-furan-2-yl]-propan-2-one oxime (7a)

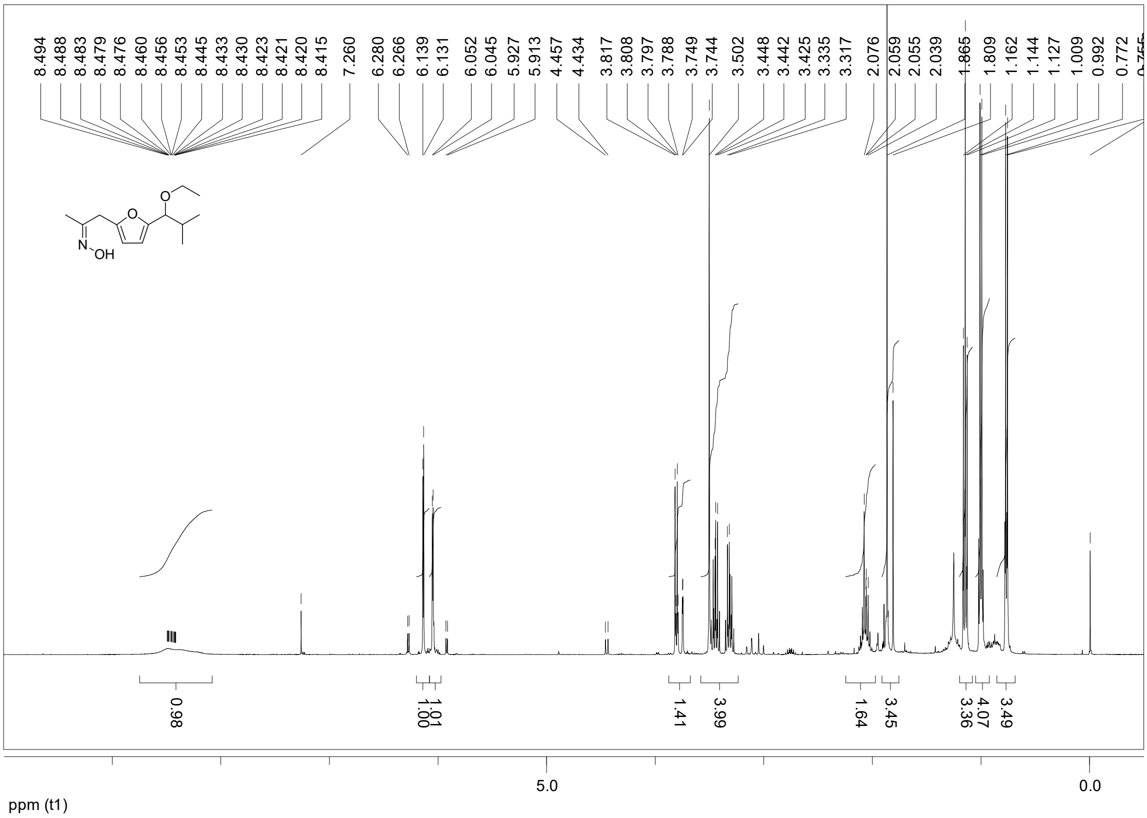

Figure S49: ^{1}H NMR spectrum ($CDCl_3$, 400 MHz) of 1-[5-(1-Ethoxy-2-methyl-propyl)-furan-2-yl]-propan-2-one oxime (7b)

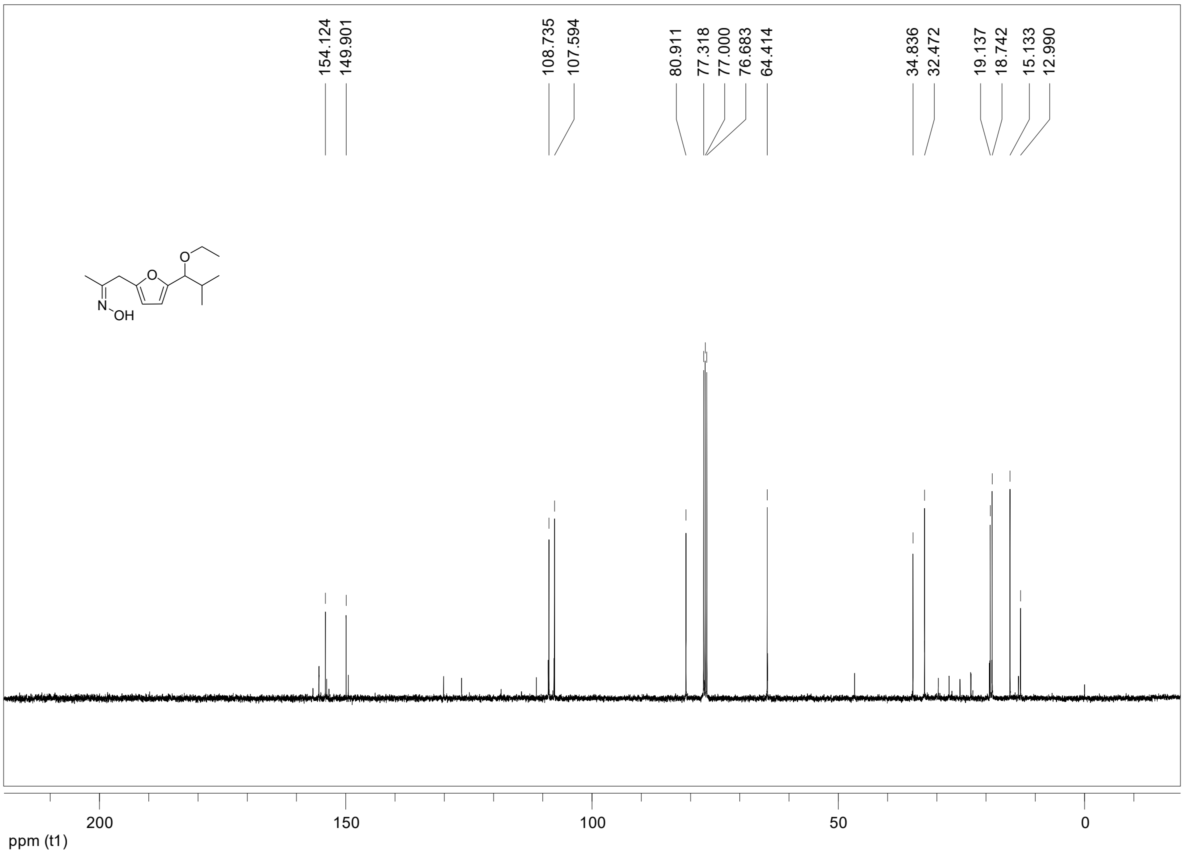

Figure S50: $^{13}C\{^{1}H\}$ NMR spectrum ($CDCl_3$, 100 MHz) of 1-[5-(1-Ethoxy-2-methyl-propyl)-furan-2-yl]-propan-2-one oxime (7b)

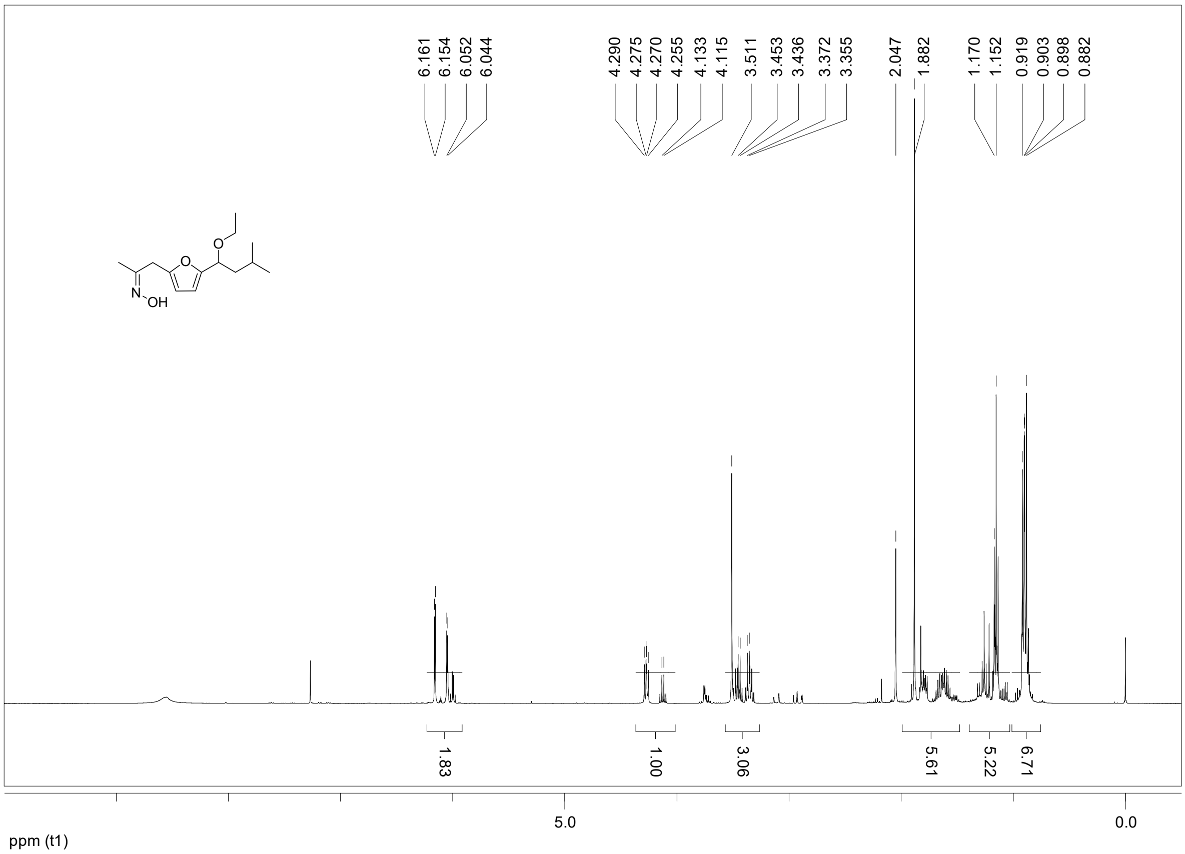

Figure S51: ^{1}H NMR spectrum ($CDCl_3$, 400 MHz) of 1-[5-(1-Ethoxy-3-methyl-butyl)-furan-2-yl]-propan-2-one oxime (7c)

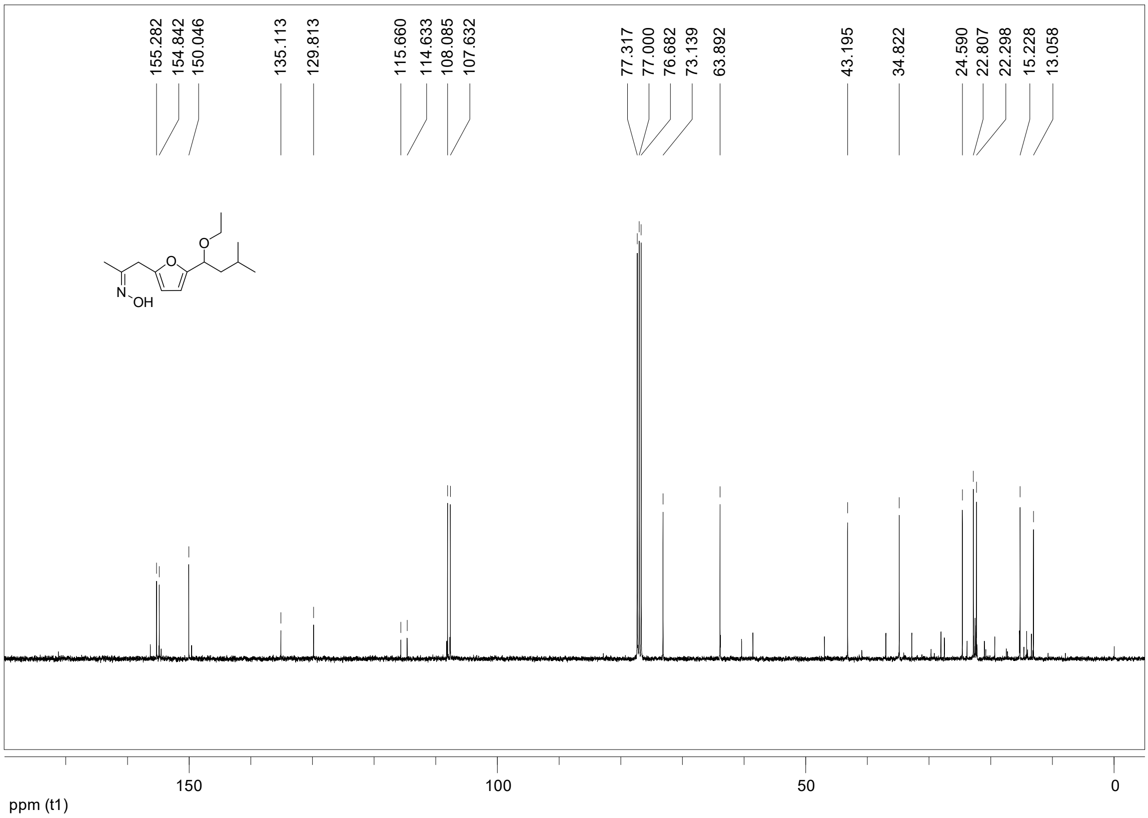

Figure S52: $^{13}C\{^1H\}$ NMR spectrum ($CDCl_3$, 100 MHz) of 1-[5-(1-Ethoxy-3-methyl-butyl)-furan-2-yl]-propan-2-one oxime (7c)

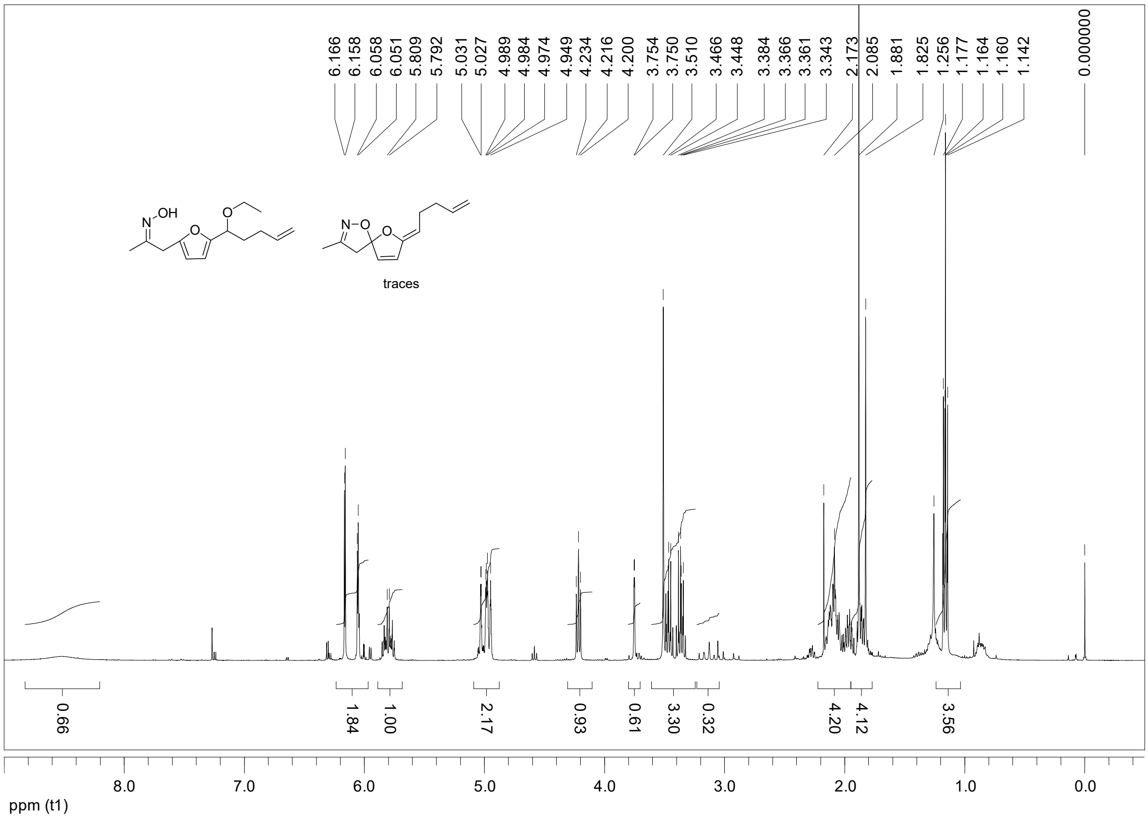

Figure S53: ^{1}H NMR spectrum ($CDCl_3$, 400 MHz) of 1-[5-(1-Ethoxy-pent-4-enyl)-furan-2-yl]-propan-2-one oxime (7d)

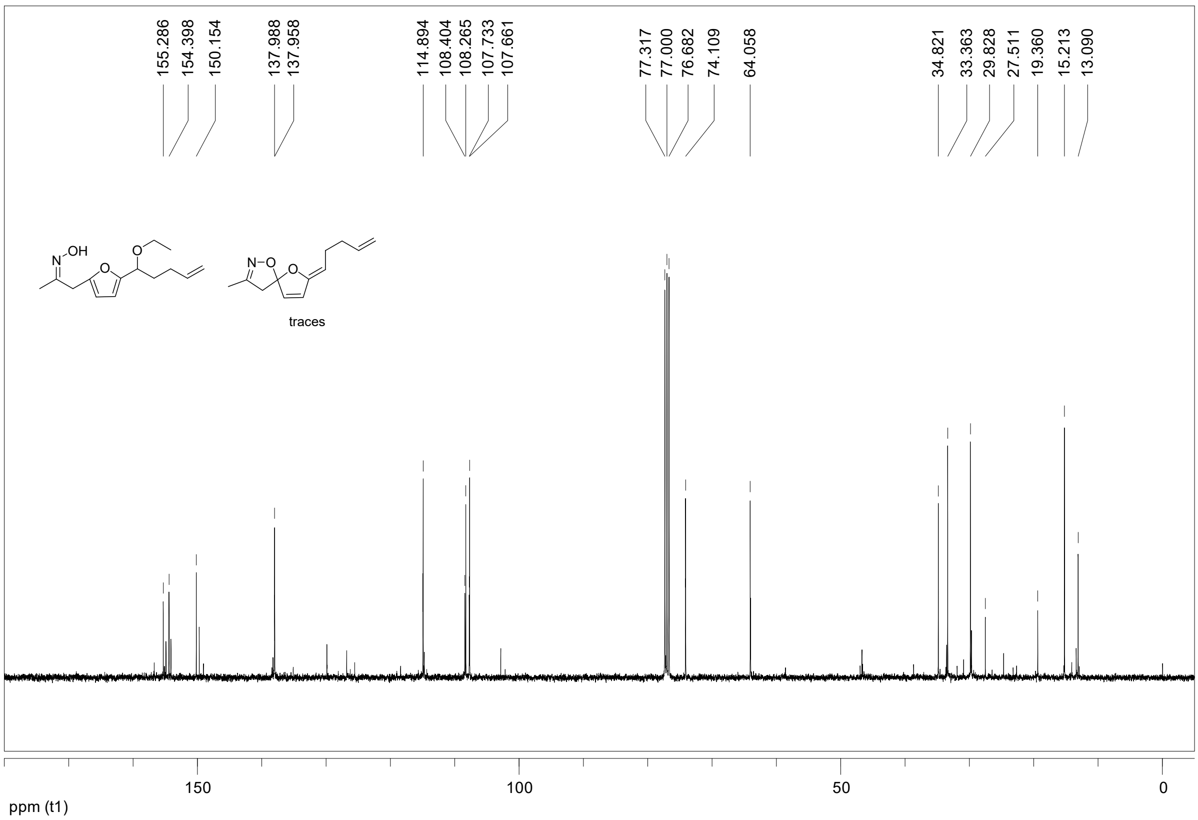

Figure S54: $^{13}C\{^{1}H\}$ NMR spectrum ($CDCl_3$, 100 MHz) of 1-[5-(1-Ethoxy-pent-4-enyl)-furan-2-yl]-propan-2-one oxime (7d)

6) NMR spectra of compounds **6c,e-f**

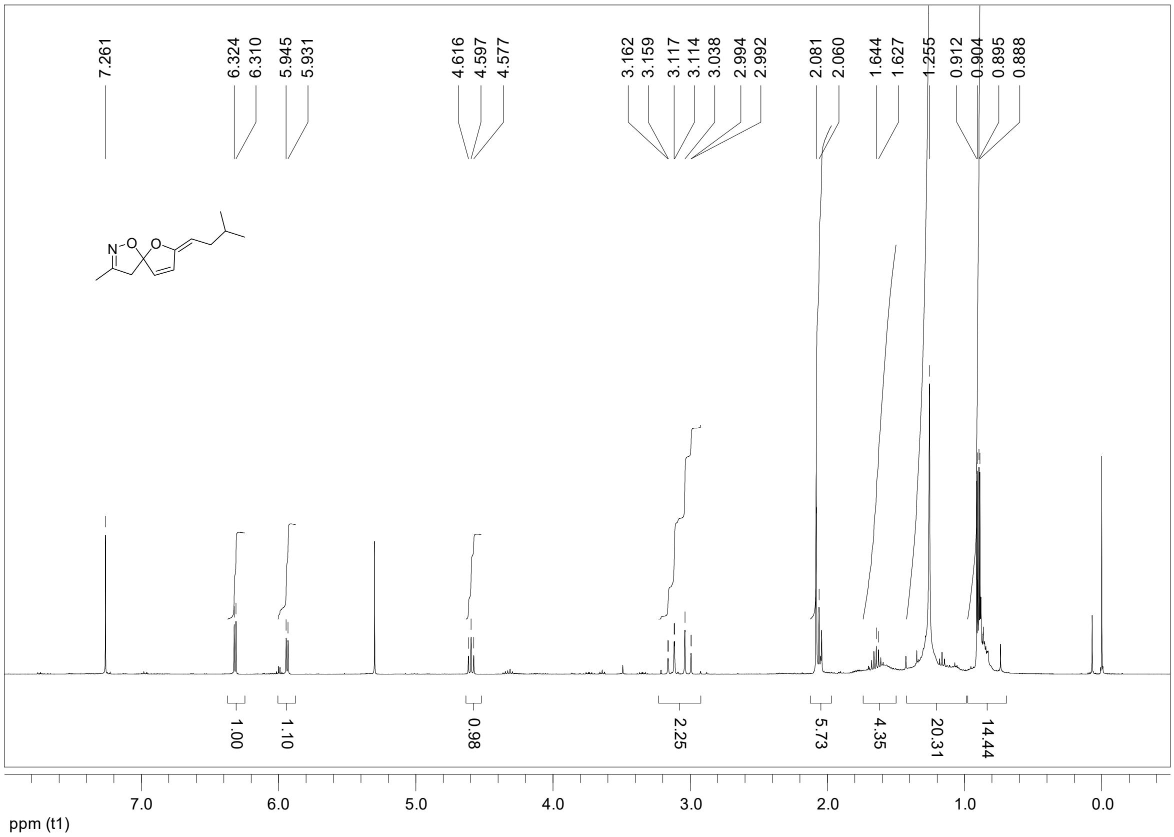

Figure S55: ^{1}H NMR spectrum ($CDCl_3$, 400 MHz) of 3-Methyl-7-(3-methyl-butylidene)-1,6-dioxa-2-aza-spiro[4.4]nona-2,8-diene (6c)

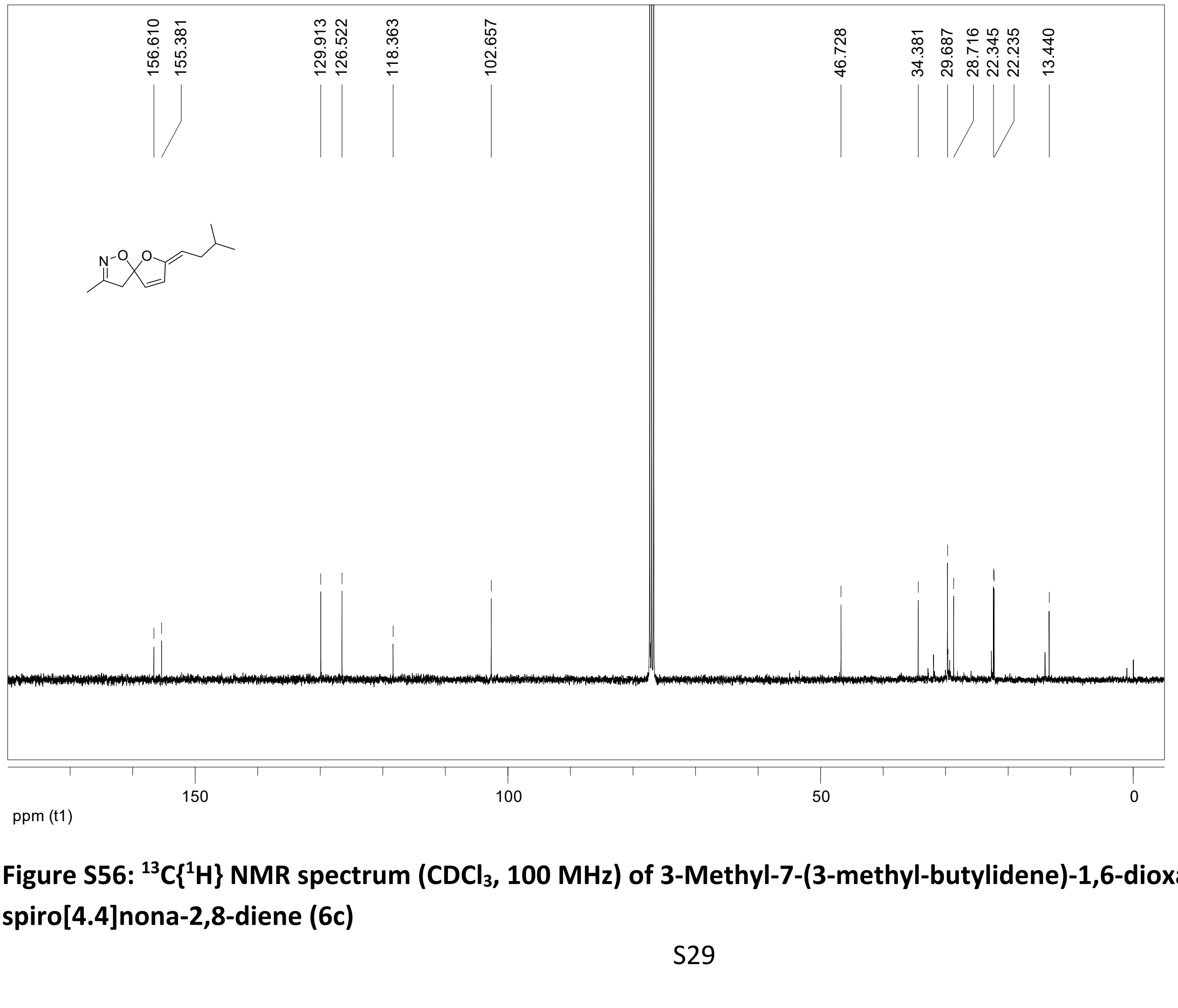

Figure S56: ^{13}C{^{1}H} NMR spectrum ($CDCl_3$, 100 MHz) of 3-Methyl-7-(3-methyl-butylidene)-1,6-dioxa-2-aza-spiro[4.4]nona-2,8-diene (6c)

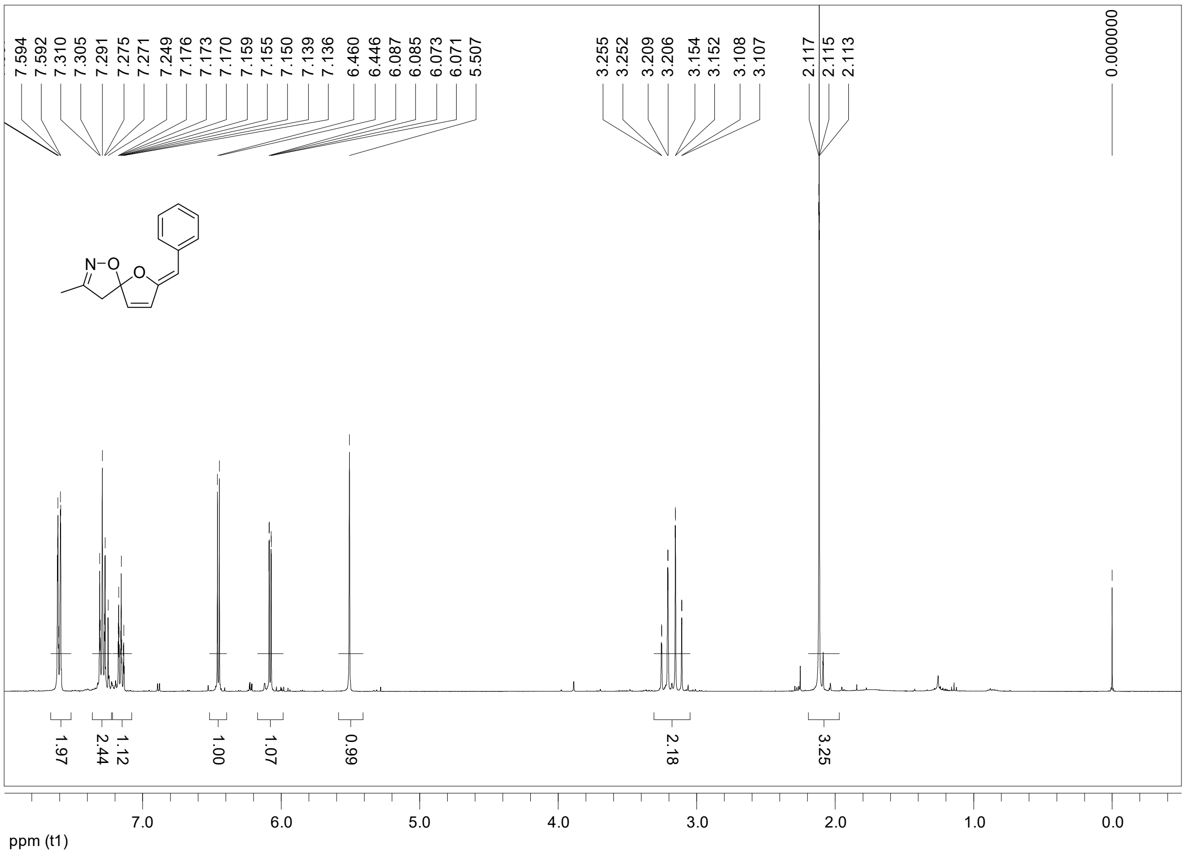

Figure S57: ^{1}H NMR spectrum ($CDCl_3$, 400 MHz) of 7-Benzylidene-3-methyl-1,6-dioxa-2-aza-spiro[4.4]nona-2,8-diene (6e)

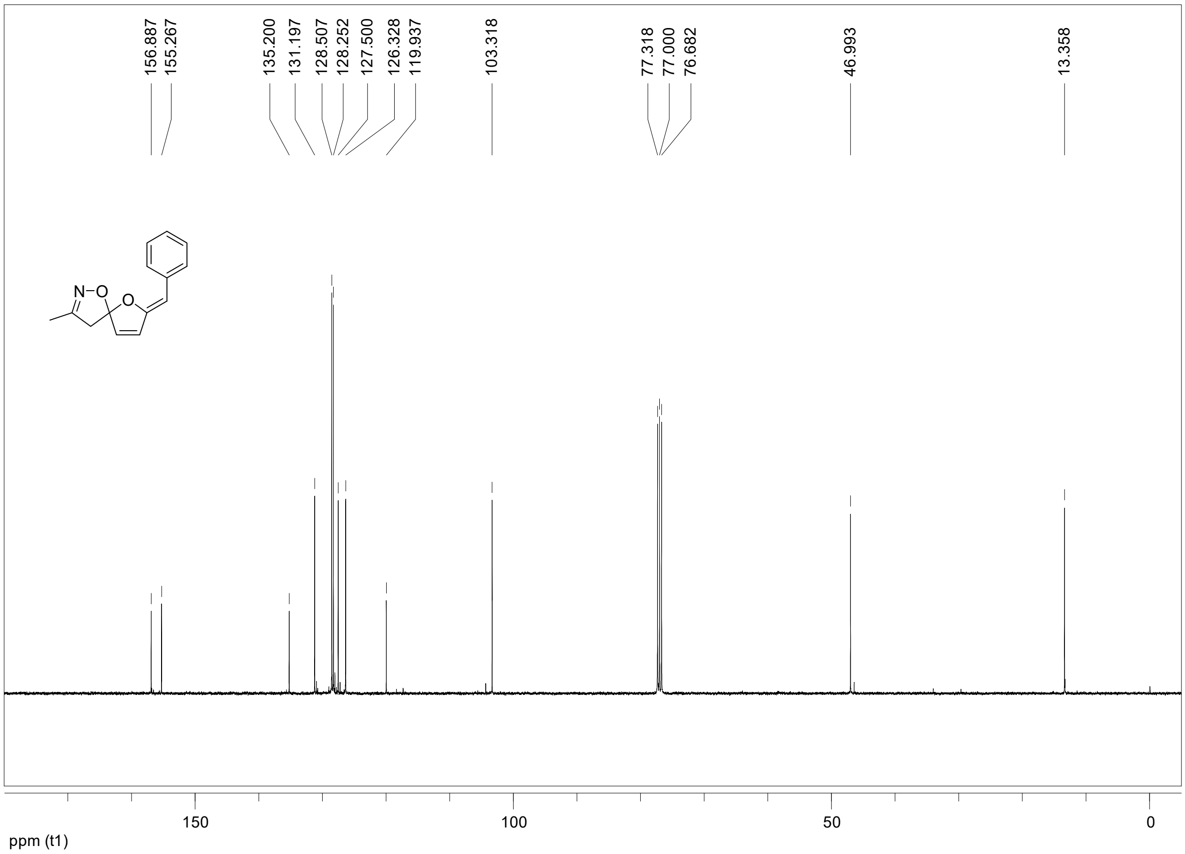

Figure S58: $^{13}C\{^{1}H\}$ NMR spectrum ($CDCl_3$, 100 MHz) of 7-Benzylidene-3-methyl-1,6-dioxa-2-aza-spiro[4.4]nona-2,8-diene (6e)

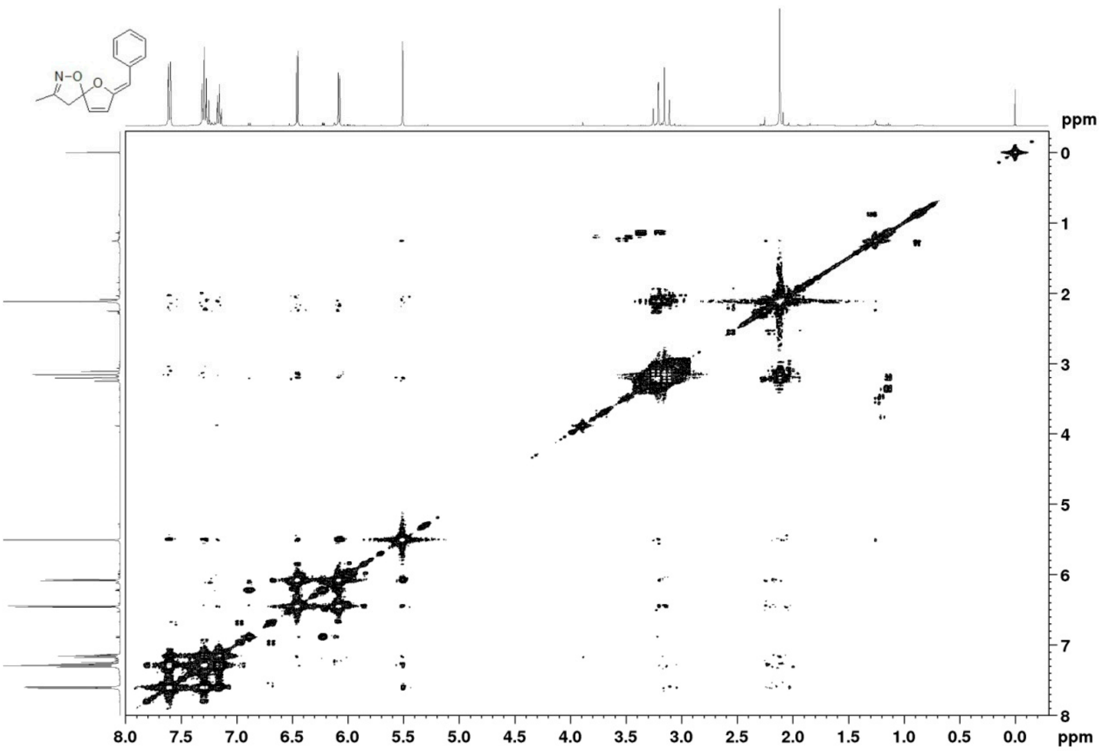

Figure S59: COSY NMR spectrum ($CDCl_3$, 400 MHz) of 7-Benzylidene-3-methyl-1,6-dioxa-2-aza-spiro[4.4]nona-2,8-diene (6e)

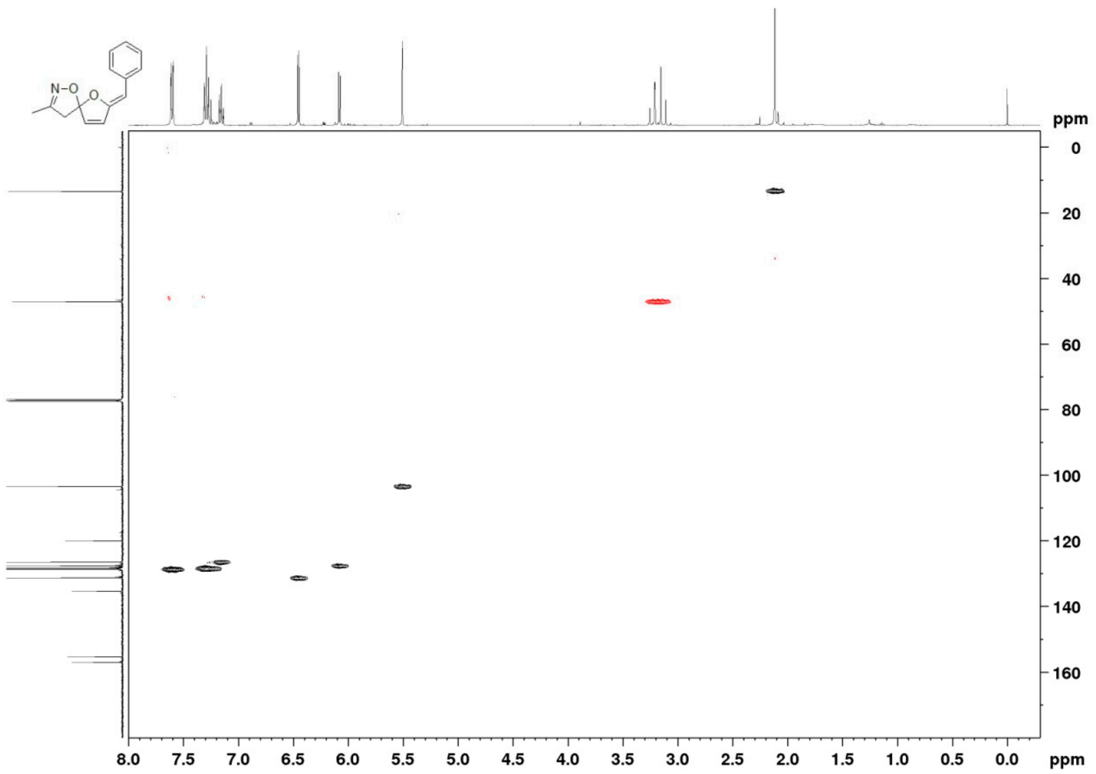

Figure S60: HSQC NMR spectrum ($CDCl_3$, 400 MHz) of 7-Benzylidene-3-methyl-1,6-dioxa-2-aza-spiro[4.4]nona-2,8-diene (6e)

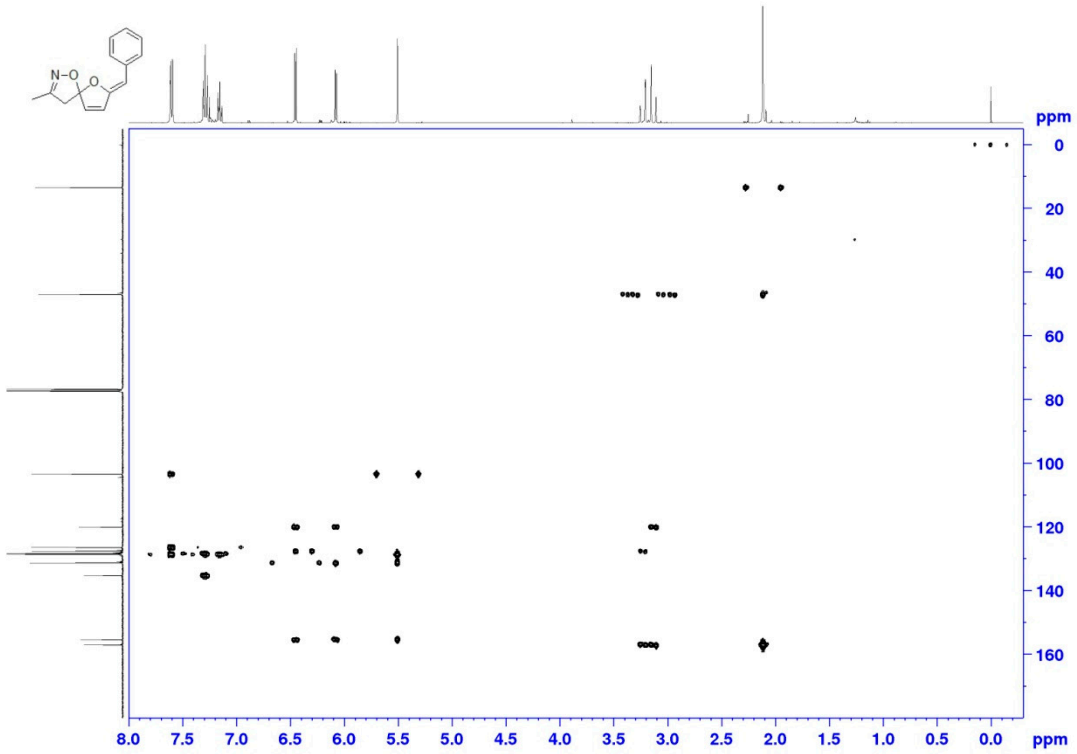

Figure S61: HMBC NMR spectrum ($CDCl_3$, 400 MHz) of 7-Benzylidene-3-methyl-1,6-dioxa-2-aza-spiro[4.4]nona-2,8-diene (6e)

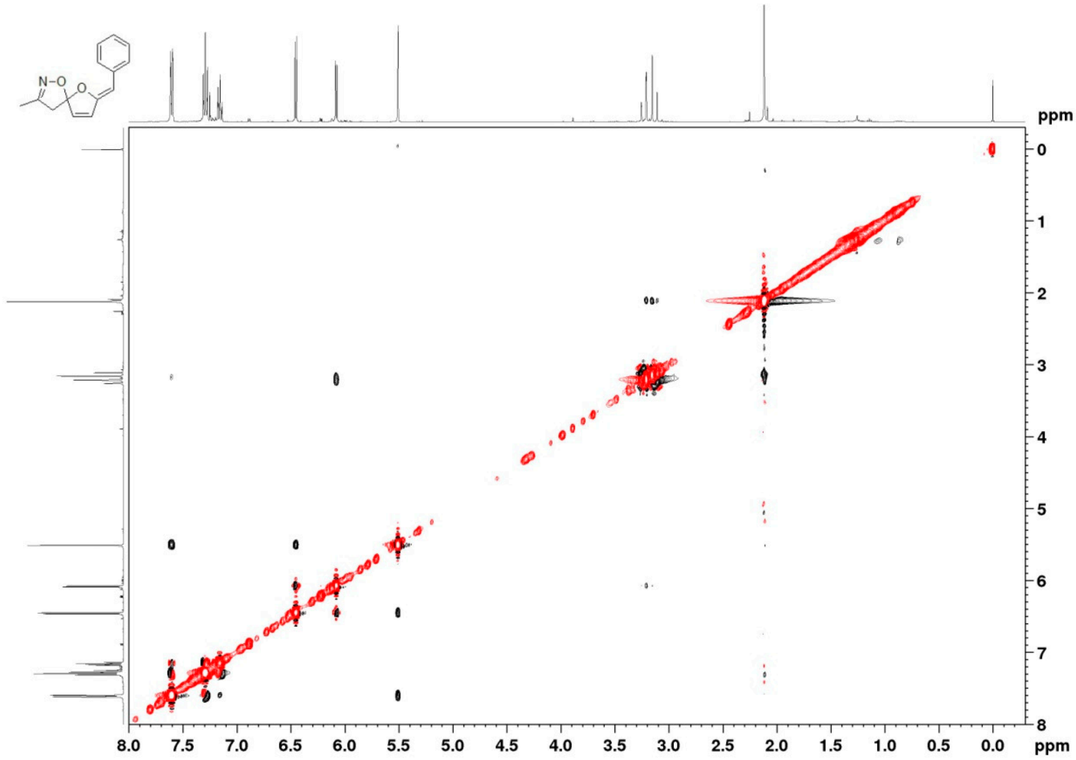

Figure S62: NOESY NMR spectrum ($CDCl_3$, 400 MHz) of 7-Benzylidene-3-methyl-1,6-dioxa-2-aza-spiro[4.4]nona-2,8-diene (6e)

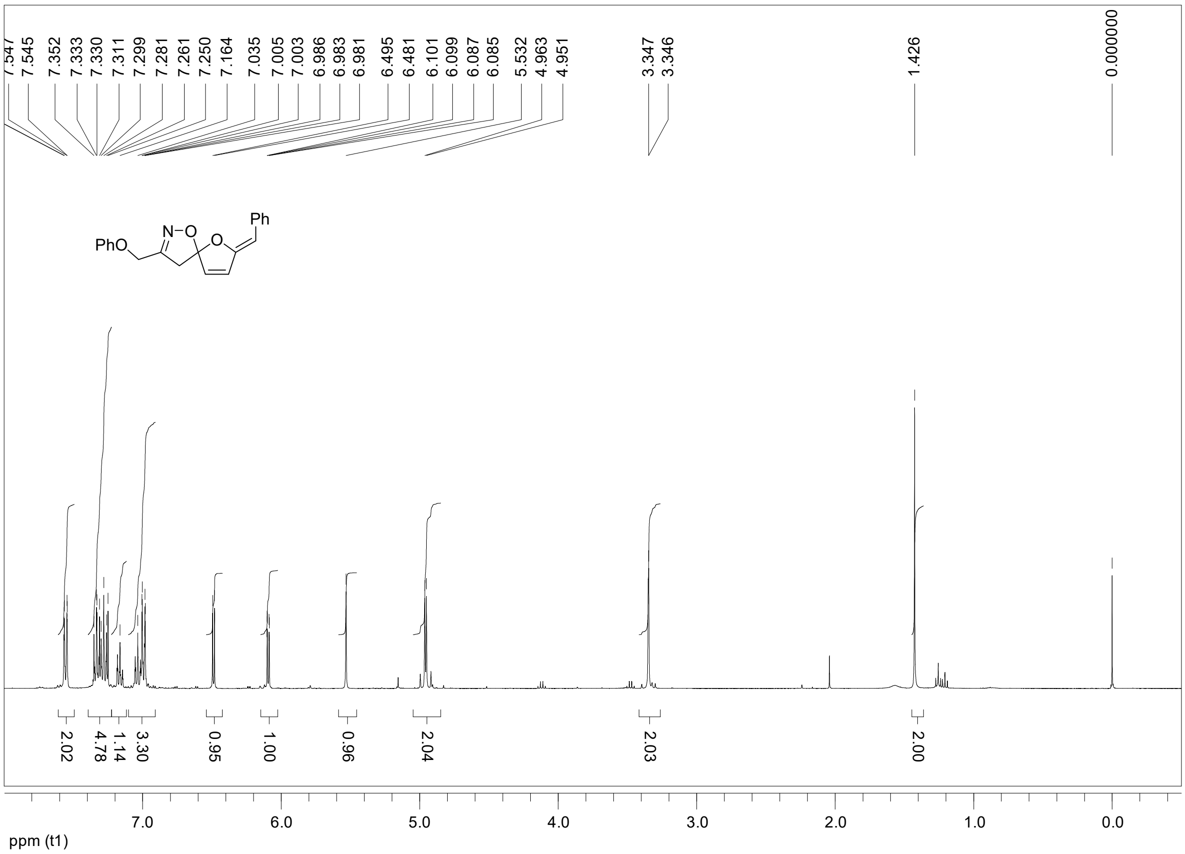

Figure S63: ^{1}H NMR spectrum ($CDCl_3$, 400 MHz) of 7-Benzylidene-3-phenoxymethyl-1,6-dioxa-2-aza-spiro[4.4]nona-2,8-diene (6f)

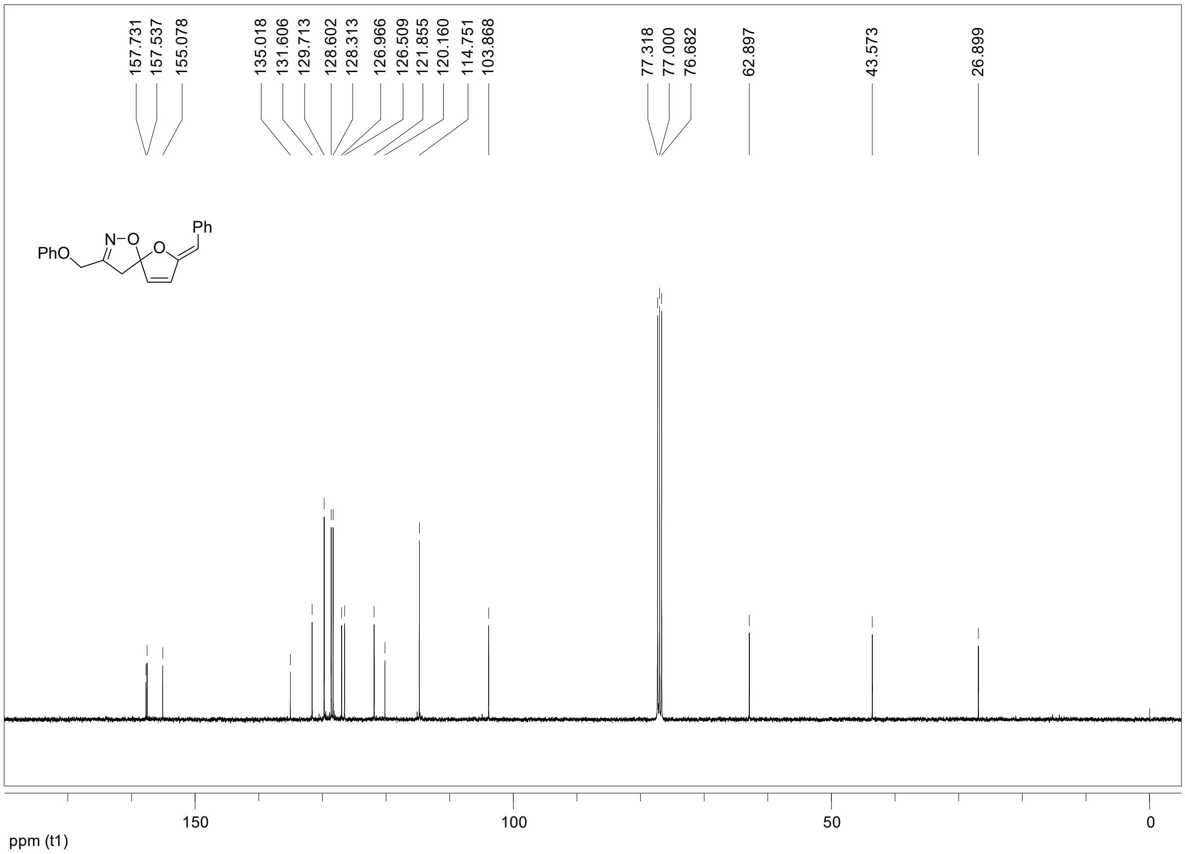

Figure S64: ^{13}C{^{1}H} NMR spectrum ($CDCl_3$, 100 MHz) of 7-Benzylidene-3-phenoxymethyl-1,6-dioxa-2-aza-spiro[4.4]nona-2,8-diene (6f)

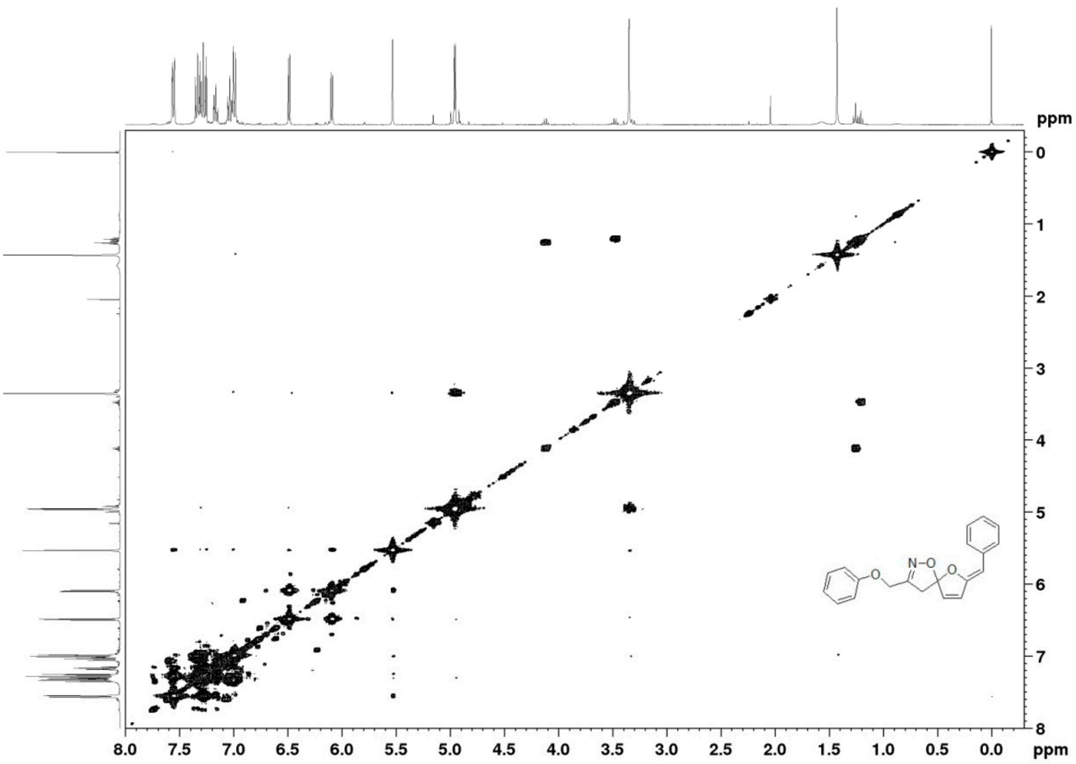

Figure S65: COSY NMR spectrum ($CDCl_3$, 400 MHz) of 7-Benzylidene-3-phenoxymethyl-1,6-dioxa-2-aza-spiro[4.4]nona-2,8-diene (6f)

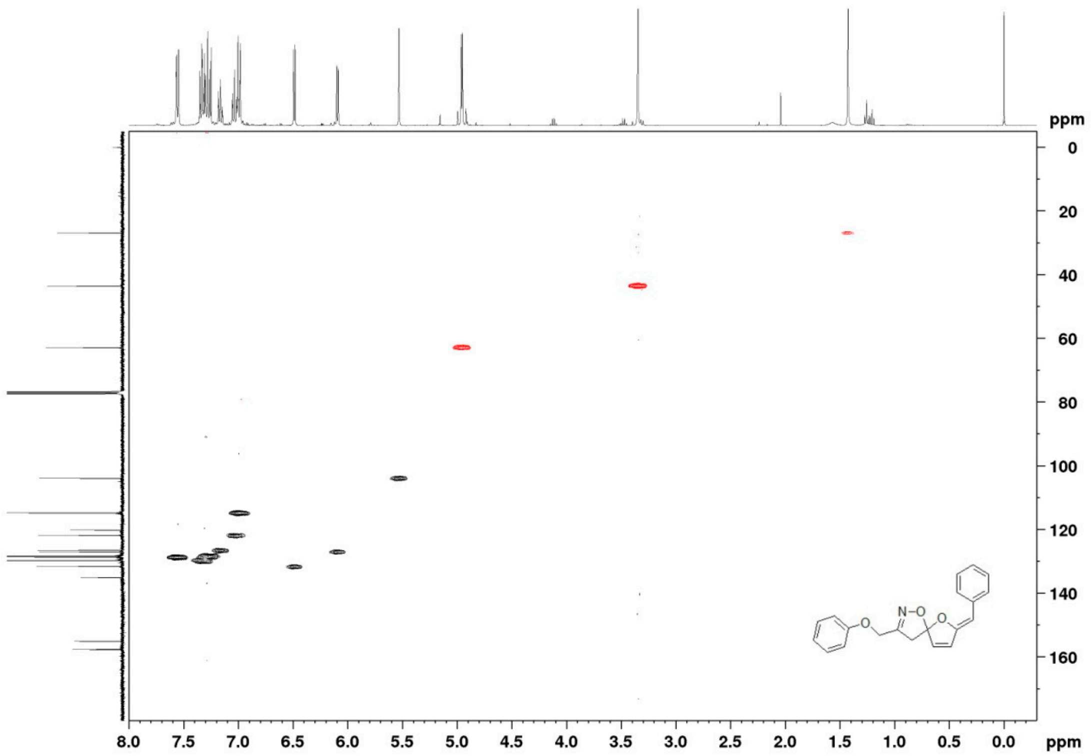

Figure S66: HSQC NMR spectrum ($CDCl_3$, 400 MHz) of 7-Benzylidene-3-phenoxymethyl-1,6-dioxa-2-aza-spiro[4.4]nona-2,8-diene (6f)

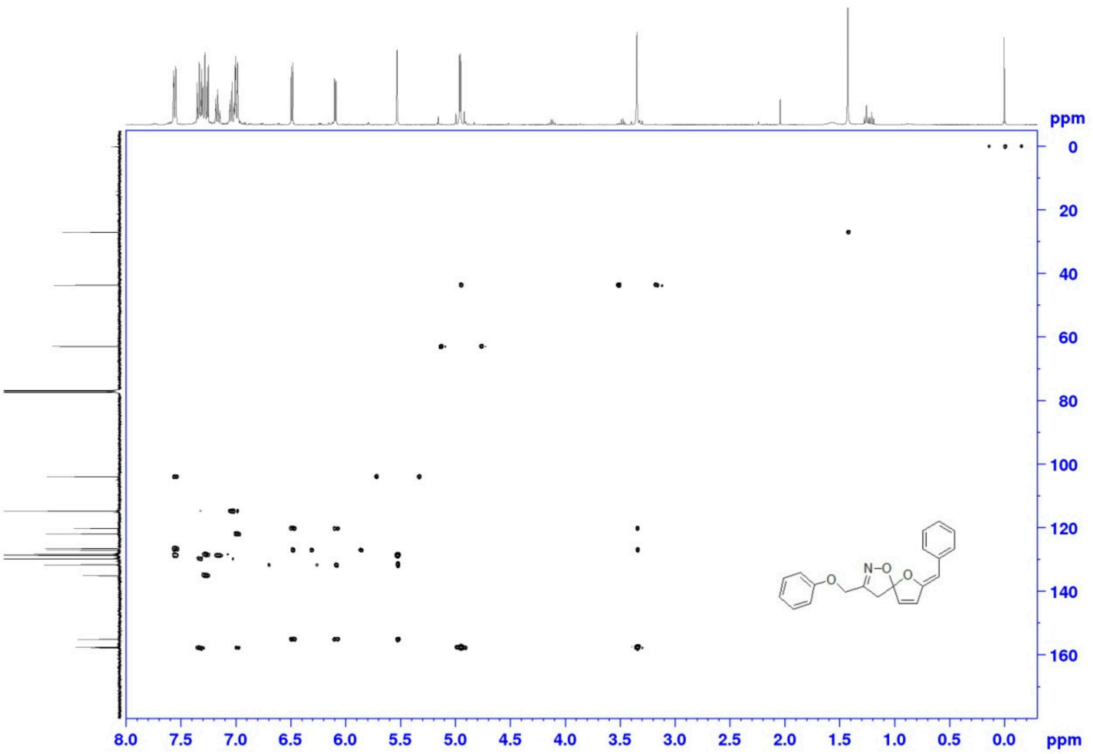

Figure S67: HMBC NMR spectrum ($CDCl_3$, 400 MHz) of 7-Benzylidene-3-phenoxymethyl-1,6-dioxa-2-aza-spiro[4.4]nona-2,8-diene (6f)
